# Combating New Delhi metallo-beta-lactamase-1 (NDM-1): an integrated review of inhibitor development and next-generation therapeutic platforms

**DOI:** 10.1039/d6ra04475a

**Published:** 2026-09-04

**Authors:** Akankhya Mohanty, Rajesh Kumar Sahoo, A. Swaroop Sanket, Ellojita Rout

**Affiliations:** a Centre for Biotechnology, Siksha ‘O’ Anusandhan (Deemed to be University) Kalinga Nagar, Ghatikia Bhubaneswar-751003 Odisha India rajeshkumarsahoo@soa.ac.in akankhyamohanty5@gmail.com swaroop.sanket@gmail.com +91-9861393365; b Directorate of Research, Malla Reddy Vishwavidyapeeth (Deemed to be University) Hyderabad 500055 India e28rout@gmail.com +91-7008829599

## Abstract

New Delhi metallo-β-lactamase-1 (NDM-1), which has emerged globally, exhibits resistance to almost all β-lactam antibiotics, including carbapenems, posing a major challenge to modern antibiotic therapy. Deep sequencing has revealed the evolution of several new NDM variants (some of the variants are more thermostable than NDM-1) due to mutations that increase their zinc-binding and catalytic efficiency and establish a selective pressure on the enzymes. In addition to enzymatic breakdown, NDM-mediated resistance is exorbitantly exhibited *via* porin loss, efflux pump activity, biofilm formation, and outer membrane vesicle-mediated dissemination. Despite the urgent need for effective countermeasures, no clinically approved inhibitor of NDM-1 has been developed. This review explores the structural and functional characteristics of NDM-1, together with the epidemiology of *bla*_NDM-1_-harbouring bacterial pathogens. It further elaborates the catalytic basis of the broad-spectrum β-lactam hydrolysis mechanism, attributed to the presence of dinuclear Zn(ii); the conserved αβ/βα metallo-β-lactamase fold, and flexible active-site loops that govern substrate recognition are also highlighted. In addition, this review provides details on the known inhibitor classes, including thiol-based compounds, hydroxamates, bicyclic boronates, repurposed drugs, and natural products, and discusses the challenges related to pharmacokinetics, toxicity, and variant-specific effectiveness. Other emerging approaches, such as CRISPR-Cas systems for targeted gene editing, nanoparticle-based delivery platforms, and artificial intelligence-driven drug discovery, are also explored as promising options for future therapy development.

## Abstract 2

Illustrative mechanistic elaboration of the NDM-1 protein and its plausible biophysical mitigation strategies (image partly generated by the Nano Banana 2 tool).

## Graphical abstract

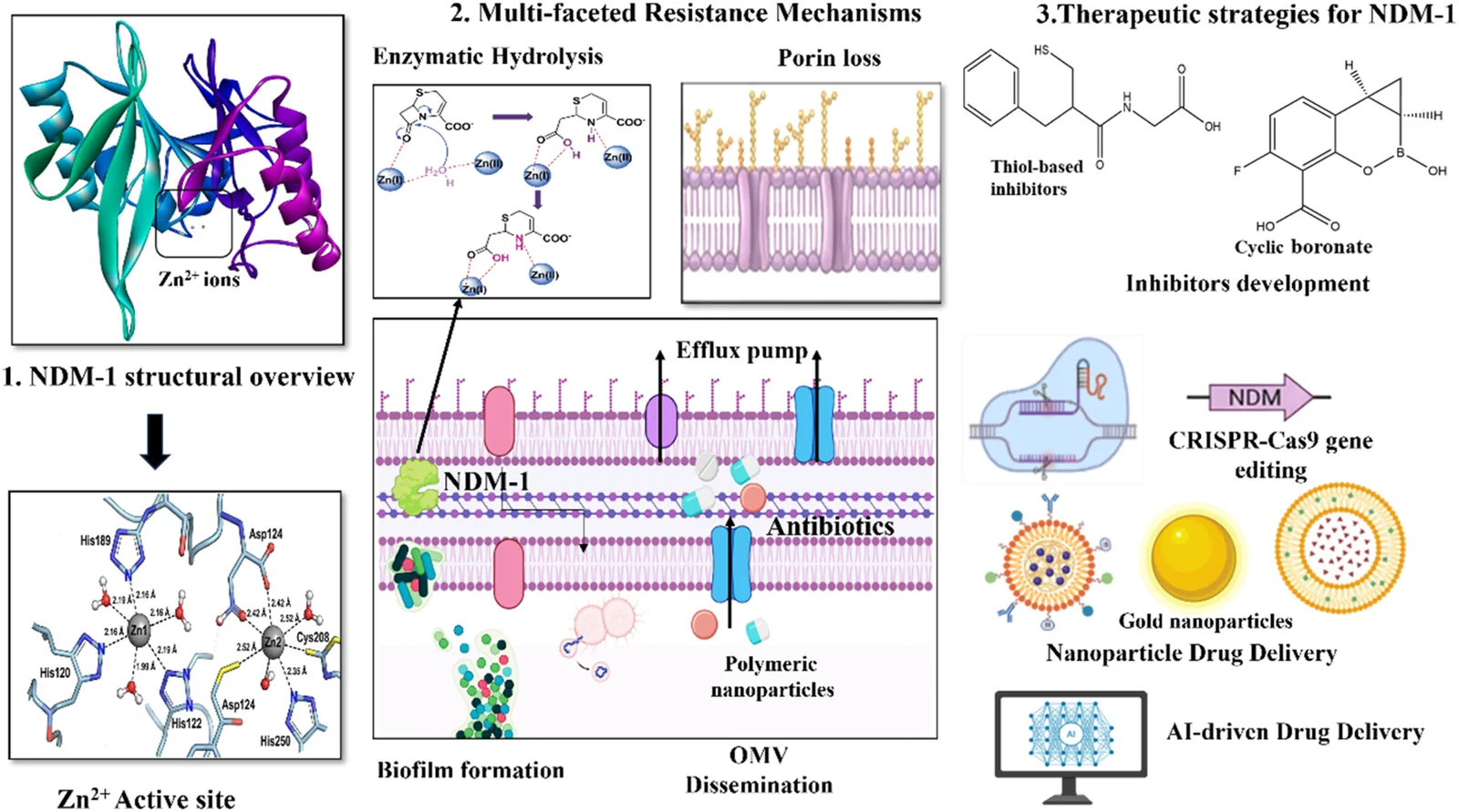

## 1. Introduction

The worldwide crisis of antibiotic resistance has accelerated in recent decades, posing a severe threat to public health, especially with the advent of multidrug-resistant (MDR) Gram-negative bacteria.^1^ The most alarming resistance mechanisms involve the expression of β-lactamase enzymes (both serine and zinc ions within the binding cavity).^2^ Zinc-containing enzymes are referred to as metallo-β-lactamases (MBLs). These enzymes confer resistance to a wide range of β-lactam antibiotics, including carbapenems.^3^ Other clinically significant MBLs, including Verona integron-encoded metallo-β-lactamase (VIM),^4^ imipenemase (IMP)^4^ and São Paulo metallo-β-lactamase (SPM),^5^ have also been widely reported. Among the MBLs, the New Delhi metallo-β-lactamases (NDMs) are widely disseminated in clinical settings, leading to a high rate of nosocomial infections. These enzymes employ an evolutionarily conserved catalytic mechanism that hydrolyzes the β-lactam ring using zinc, rendering antibiotics ineffective. VIM and IMP variants are widely distributed across Europe and Asia, and SPM-type enzymes are most commonly reported in South America, suggesting the global as well as regional clustering of MBLs. Nevertheless, NDM-1 has acquired a broad substrate specificity and extraordinary genetic mobility, often in combination with plasmids containing multiple resistance determinants, thereby facilitating rapid horizontal transmission across a wide range of bacterial species and environments. NDM-1 has spread at an alarming rate and has become a symbol of the new post-antibiotic times since its initial discovery in 2008 from a *Klebsiella pneumoniae* (*K. pneumoniae*) isolate from a Swedish patient who had travelled to India.^6^

NDM-1 is a class B β-lactamase in the Ambler classification and subclass B1 of MBLs, which has either one or two zinc ions at the active site required for its catalytic function.^7^ NDM-1 inactivates an array of β-lactams, such as penicillin, cephalosporins, and carbapenems, and is the last resort to combat MDR infections, except monobactams, such as aztreonam. However, the co-production of extended-spectrum β-lactamases (ESBLs) or ampicillin class C (AmpC) enzymes with other β-lactamases can make even monobactams, such as aztreonam, ineffective, even though they are naturally resistant to some β-lactamases. This enzymatic flexibility, combined with the genetic flexibility and mobilizable nature of the NDM gene, has facilitated rapid horizontal transmission across species and geographic regions.^8^

NDM-1-mediated resistance is particularly challenging because β-lactamase inhibitors such as clavulanic acid, tazobactam, and sulbactam cannot target MBLs, including NDM-1. In contrast to serine β-lactamases (SBLs), where the action depends on a catalytic serine residue, NDM-1 uses zinc-coordinated hydroxide ions for the cleavage of the β-lactam ring, thus making the process of inhibition more complicated. Therefore, clinically effective inhibitors of NDM-1 are still the most pressing challenge in antimicrobial drug research.^9^ The effect of NDM-1 is intensified by its association with multiple bacterial clones, including the ST258 *K. pneumoniae* lineage and ST131 *E. coli*.^10^ Such clones exhibited elevated levels of virulence, with enhanced biofilm production.^10^ The widespread detection of NDM-1 producing bacteria across different environments signifies the enzymes' stability and rate of dissemination.^11^ The efforts to discover countermeasures have fuelled research into development of various NDM inhibitor classes. These classes can be classified as thiol-based inhibitors, small-molecule chelators, broad-spectrum chelators, peptides, and protein-based inhibitors.^8^ Some of these inhibitors,^8^ including aspergillomarasmine A^12^ and captopril,^13^ have been vividly explored against NDM and have been proved to be efficient. Furthermore, the emergence of NDM-1 variants such as NDM-4, NDM-5, and NDM-7, each with subtle structural differences that affect activity and inhibitor binding, adds another level of complexity to inhibitor design.

The physiological properties of NDM-1 inhibitors have greatly hindered the process of developing effective therapeutic medications. Furthermore, there is still a significant challenge of achieving high specificity for the bacterial enzyme while remaining safe for humans. As a result, no approved inhibitor has been identified in clinical trials, and patients at risk of infection with NDM-1-producing bacteria have few treatment options. The implications of this crisis are wide-reaching. Low morbidity, mortality, and healthcare costs are associated with infections caused by NDM-1-producing bacteria in the absence of effective treatments. Moreover, the rapid spread of these resistant strains has been ongoing, as the clinical efficacy of currently available antibiotics, used over the years, has been undermined, and there is an urgent need to develop novel and effective therapeutic interventions.

Metallo-β-lactamase (MBL)-producing Gram-negative pathogens, especially carbapenem-resistant *K. pneumoniae*, *Escherichia coli* (*E. coli*), *Pseudomonas aeruginosa* (*P. aeruginosa*) and *Acinetobacter baumannii* (*A. baumannii*), are important causes of hospital-acquired bloodstream infections, pneumonia, urinary tract infections, surgical site infections and sepsis. Such infections are accompanied by prolonged hospitalization, increased healthcare costs, and high mortality due to limited therapeutic options. The clinical management is further complicated by the frequent co-existence of other resistance mechanisms, such as extended-spectrum beta-lactamases (ESBLs), porin loss, efflux pump overexpression and biofilm formation, which can threaten the efficacy of even last-line antibiotics. Although aztreonam-based combinations and the newly developed β-lactam/β-lactamase inhibitor therapies have shown promise, the treatment outcomes are still variable, and there is no clinically approved inhibitor that specifically targets NDM-type MBLs. Therefore, there is an urgent clinical need for novel MBL inhibitors and alternative therapeutic strategies that can restore antibiotic susceptibility and improve patient outcomes.

## 2. General architecture of NDM-1

**Fig. 1:**
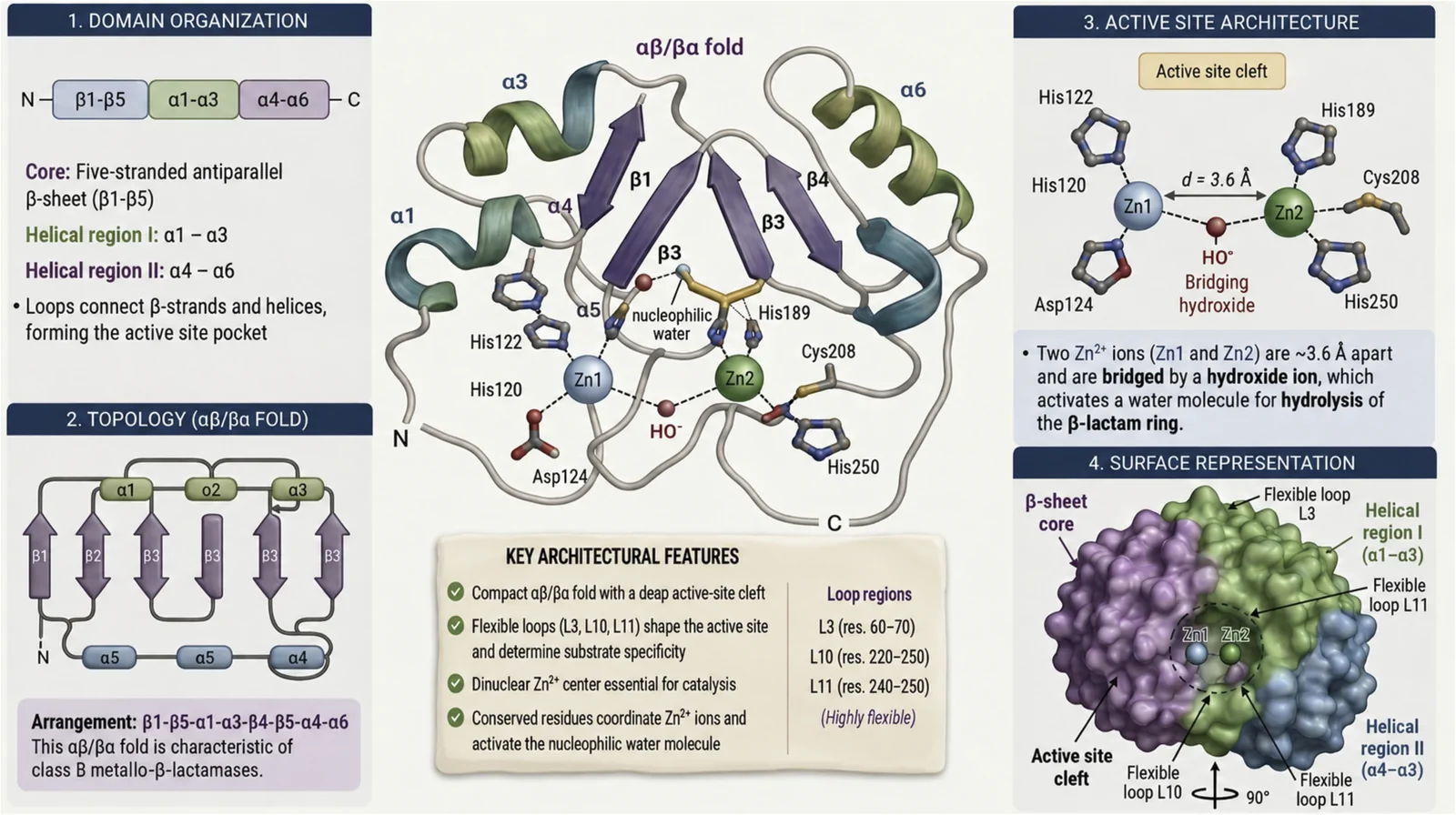
General architecture of NDM-1 with reference to its structural fold (1) domain organization (2) topology (3) active site (4) surface representation (image generated using Adobe Firefly Image v3).

NDM-1 is a 270-residue enzyme of approximately 27.5 kDa molecular mass, which is consistent with other B1 subclass of MBLs (Fig. 1). Architecturally, NDM-1 showcases a compact, approximately 50 Å × 40 Å × 40 Å globular fold in the form of a four-tiered β-sandwich (αβ/βα fold), a characteristic of the MBL superfamily, in which two antiparallel β-sheets constitute the core and are surrounded on their outer faces by α-helices.^14^ This structure is further divided into two major subdomains. The N-terminal subdomain includes two α-helices (α1 and α2) and seven antiparallel β-strands (β1 to β7), while the C-terminal subdomain consists of two α-helices (α4 and α5) and five antiparallel β-strands (β8 to β12). These units are linked by flexible loop regions that not only confer structural stability but also define the active-site structure, which is important for catalysis.^15^ NDM-1 is characterized by significantly longer N-terminal elements, including two additional β-strands, which may further aid substrate binding through enhanced hydrophobic interactions, suggesting a functionally refined nuance in loop construction.^16,17^ Additionally, NDM-1 typically exists as a monomer in solution, although it can assume transient dimers, possibly mediated by unique loop insertion involving residues Thr162 to Gly167, through hydrophobic and van der Waals interactions.^14^ The highly conserved αβ/βα core provides both structural stability and catalytic competence consistent with the enzyme's wide substrate range among B1 MBLs.

## 3. Variants of NDM and their global epidemiology

**Fig. 2:**
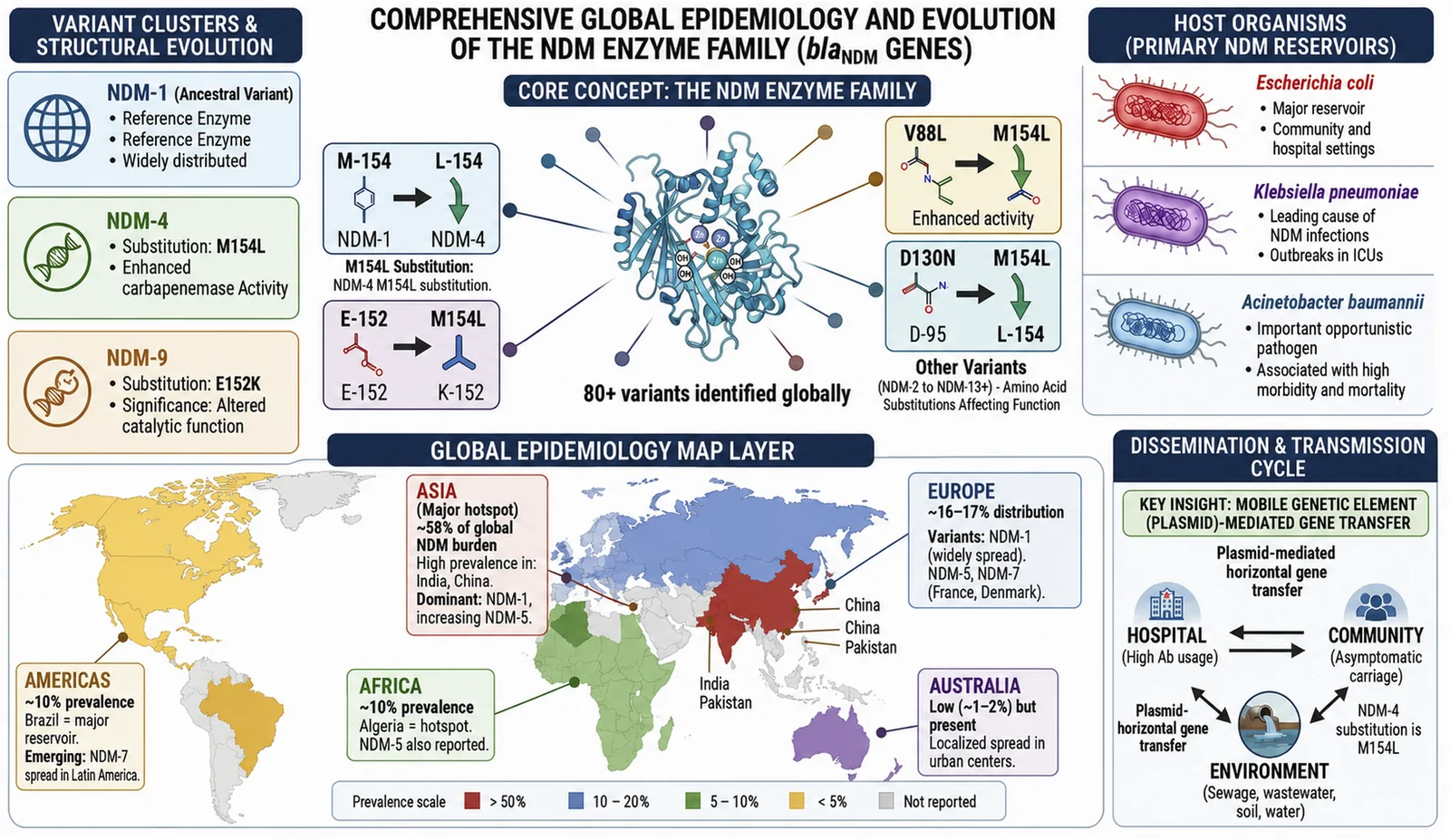
Variants of NDM since its discovery in 2008 (image generated using Adobe Firefly Image v3).

Since its initial report in 2008, NDM-1 has been responsible for a very large and growing family of sequence variants (Table S1, SI and Fig. 2). Over 40 different NDM alleles have been documented, and new isolates have even reported NDM-60, demonstrating ongoing diversification of NDM under worldwide selective pressures.^18,19^ These variants differ by one or several amino-acid substitutions that lie outside the catalytic zinc-binding residues but still modulate enzyme activity by influencing substrate selectivity, catalytic proficiency, thermostability, zinc affinity, and tolerance to host-imposed metal limitation.^20^

Systematic biochemical surveys reveal that most NDM variants have higher thermostability up to ∼10 °C enhanced *T*_m_ and higher Zn^2+^ affinity up to ∼10-fold lower *K*_d_ for Zn^2+^ than NDM-1, modifications that confer variants a selective advantage, especially when Zn is limiting the nutritional immunity instigated by the host.^20^ Functional studies also show that certain substitutions, such as Val88 → Leu and Met154 → Leu, enhance the carbapenem catalytic turnover or improve folding and refolding kinetics, which help explain the relative prevalence *in vitro* of variants such as NDM-5, which exhibit increased carbapenem hydrolytic activity and clinical prevalence.^21^ Genomic and epidemiological studies state that the diversification of NDM is closely linked to the horizontal gene transfer. Specifically, NDM-1 is mobilized across a wide spectrum of mobile elements, such as Tn125/ISAba125, IS26-linked composite transposons, integrons, and broad-host-range conjugative plasmids, particularly IncX3, IncF, and IncA/C. These mobile elements facilitate interspecies dissemination between human, animals, and environmental reservoirs.^22^ Epidemiological trends remain dynamic yet NDM-1 continues to be the most prevalent variant worldwide. Emerging variants such as NDM-4, NDM-5, and NDM-7 show increased frequency across diverse geographic regions. NDM-5 is frequently reported in *E. coli* lineages ST167 and ST410, driven by both clonal spread and plasmid-mediated transfer.^23^ Together, these molecular and epidemiological characteristics, mutational optimization of enzymatic activities, selection within zinc-restricted host habitats, and mobilization on versatile genetic platforms have enabled NDM to spread rapidly and become a worldwide, ubiquitous threat to β-lactam effectiveness.^20^

### 3.1. Functional consequences of NDM mutations

Typically, NDM mutations consist of one or several changes in the amino acid sequence that usually occur away from the universally conserved Zn^2+^ coordinating amino acids. These mutations impact on protein folding, structural stability, Zn^2+^ binding, and enzymatic activity.^20^ Biophysical and biochemical analyses have identified multiple mutational hotspots within the NDM family such as Val88, Asp130, Met145, Ala233, Glu170, and others (Pro28 and Asp95), At these positions, the amino acid substitutions frequently occur, leading to predictable alterations in the enzyme properties and functional behaviour.^24,25^ The triple mutant Val88Leu + Met154Leu + Ala223Val, as found in engineered or naturally evolved variants (*e.g.*, NDM-16), has been shown to increase the apparent melting temperature by almost 10 °C relative to NDM-1, suggesting a significant improvement in Zn^2+^ binding.^20^ The common Met154Leu mutation has been shown to increase Zn^2+^ affinity, and some evolved variants, such as NDM-15, have been reported to be mono-zinc enzymes that show significant activity even when only one metal ion is bound.^20^ Host innate immunity depletes the transition metals (nutritional immunity), so variants with increased Zn^2+^ affinity or that are mono-zinc enzymes are favored *in vivo*, as they can function with low levels of free Zn^2+^.^20^ Biochemical analysis of enzyme functions shows that some mutations directly affect their catalytic activity, for example, the Met154leu mutation, associated with enhanced carbapenem hydrolysis in NDM-4, and the additional Val88Leu mutation in NDM-5, which is a modifier of activity toward specific substrates, leading to variants with particularly altered *k*_cat_/*k*_m_ profiles.^20^ Epistatic effects of cooperative mutations, such as NDM-17 (Val88Leu, Met154Leu, and Glu170Lys), have been experimentally demonstrated to confer higher substrate affinities and catalytic efficiencies towards β-lactam antibiotics compared with double mutations, suggesting that such interactions can increase the enzyme's catalytic capacity.^25^ The clinical and ecological significance of molecular adaptation is evident in most recently described alleles. Notably, NDM-58 (Pro185Ser) variant in *P. aeruginosa* introduces a novel substitution, that has been correlated with resistance against several β-lactam antibiotics and the presence of a plasmid encoding other resistance determinants.^26^ These observations illustrate how single amino acid substitution can occur across different pathogens and promote multidrug resistance. Overall, these findings suggest an evolutionary selective pressure favouring NDM variants that exhibit enhanced structural integrity, optimized Zn^2+^ binding and long-term catalytic stability under host-imposed metal limitations, which make inhibitor development more difficult due to increased flexibility of the active site.

## 4. Mechanism of NDM-1-mediated resistance

### 4.1. Basic hydrolysis mechanism

**Fig. 3:**
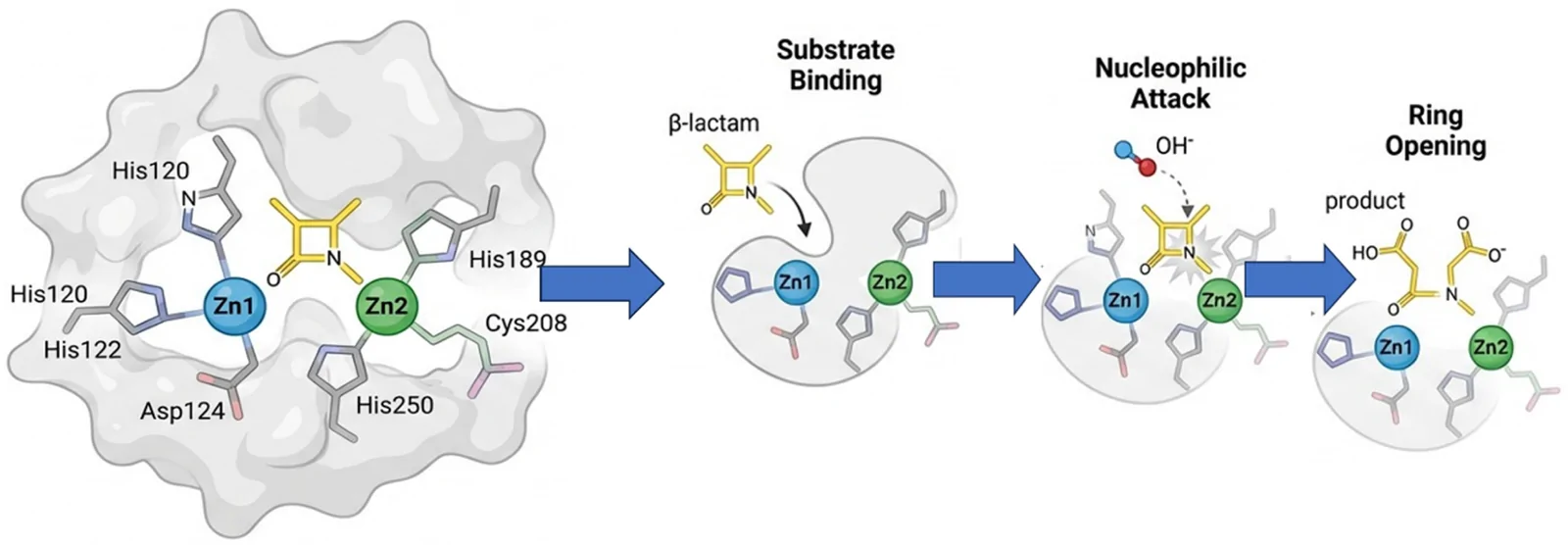
Schematic of the basic mechanism of NDM hydrolysis (image generated using Adobe Firefly Image v3).

The enzymatic process of NDM-1 involves an open and solvent-accessible active site, where two zinc atoms (Zn1 and Zn2) are coordinated by six conserved residues: His120, His122, His189 (Zn1), and Asp124, Cys208, His250 (Zn2)^27^ (Fig. 3). A hydroxide ion bridging two zinc sites acts as the nucleophile attacking the carbonyl carbon of the β-lactam ring. This attack produces a tetrahedral intermediate that collapses, breaking the C–N bond of the β-lactam ring and inactivating the antibiotic.^28^

After hydrolysis, the distance between Zn1 and Zn2 in the substrate structure increases, destabilizing the bond and allowing the hydrolysate to be released from the NDM-1 enzyme's activity site. Zn1 maintains the oxygen atom of the carboxyl group in a position where it can coordinate with the Zn2 ion, and the hydroxyl group is correctly positioned to attack the carbon atom of the carbonyl group on the β-lactam ring *via* nucleophilic attack.^29^ It also demonstrates how the hydroxide ion at the active site attacks the carbonyl carbon of the β-lactam to form an intermediate, which is then stabilized by zinc ions to create a transition state complex, eventually leading to the breaking of the C–N bond. Although the catalytic mechanism of NDM-1 remains unknown, it is believed to operate through the dual-zinc catalytic mechanism described above. A potential inhibitor could be developed as a future treatment to better understand the relationship between the active site and the hydrolysis process.^15^

### 4.2. Structural characteristics enabling general substrate specificity

NDM-1's very broad substrate profile is found in a synergy between an open, solvent-exposed active site and high loop flexibility, which together enable the enzyme to recognize structurally diverse β-lactams.^30^ Among these loops, specifically, Loop3 (residues 63–67) and Loop10 (residues 208–220) serve as dynamic “gates” that direct substrate recognition, positioning, and stabilization.^31,32^ Loop 3 is a hairpin that lines one edge of the active site cleft and is hydrophobic-rich (*e.g.*, Leu65, Met67, and Phe70).^33^ In particular, the apo structure of the flexible active site of loop L3 is longer and more random, but β-3 and β-4 assume an extended β-sheet conformation after ligand binding communication. The side chain of Met67 reorients by ∼4.9 Å away from the Zn center during this zippering process, displacing the L3 loop farther from the Zn center. This is probably achieved to accommodate the substrate by hydrophobically interacting with the R1 phenyl group of ampicillins. Conversely, when Leu65 is bound, it moves ∼2.1 Å towards the substrate and the Zn core. When combined, the residues Leu65 and the hydrophobic surface formed by Trp93 and Met67 bind strongly to the phenyl group of the substrate R1. The measured apoenzyme flexibility might be a mechanism by which MBL enzymes provide the L3 loop with the flexibility required to form substrate-specific hydrophobic interactions. Thus, the binding affinity of β-lactam antibiotics for MBLs could be altered if the hydrophobic nature of the R1 functional group is altered.^34^ Loop 10 is also important for substrate interaction following ligand binding. In particular, the Asn220 side chain nitrogen is extracted. In comparison to ampicillin-bound structures, apo structures are 1 Å nearer to the zinc centre. Such a shift surpasses the ∼0.284 Å RMS variations in superposition of the 232 shared Cα (C-alpha) atoms between the two models.^34^ The strongly conserved Asn220 residues are oriented for interaction with lactam carbonyl in the complex structure due to their translocation.^32^ The oxyanion hole formed by these conserved N220 residues and Zn1 helps polarize the lactam carbonyl upon binding and facilitates the nucleophilic attack to the neighbouring hydroxide. Together, the flexibility observed in the L3 and L10 loops allows NDM-1 to accommodate a range of substrates with diverse molecular frameworks.^34^

### 4.3. Hydrolysis of carbapenems: a unique pathway

NDM-1 hydrolyses carbapenems through a mechanism different from penicillin and cephalosporin. High-resolution crystal structures of NDM-1 complexed with hydrolyzed imipenem and meropenem, resolved by NMR for meropenem hydrolysis, suggested the presence of three catalytic species: two enzyme–intermediate states as EI_1_ and EI_2_ and the third one as enzyme–product state (EP). These analyses help to understand the transition from Δ^2^-to-Δ^1^ tautomerization of the pyrroline double bond in carbapenem, and formation of an exclusive β-diastereomeric product, and the absence of a µ-bridging water molecule between the catalytic Zn(ii) ions.^35^ These findings are consistent with the protonation of the β-face by a bulk water molecule entering the active site, and led the authors to believe that the mechanism of NDM-1-catalysed carbapenem hydrolysis is different from that of penicillin and cephalosporin hydrolysis.^35^ Wider mechanistic studies throughout MBL classes, specifically including bi-Zn(ii) NDM-1, suggest two kinetically linked anionic intermediates following nucleophilic attack on carbapenems. In this context, EI_1_ may be N-protonated, producing a Δ^2^ tautomer, whereas EI2 may be C-2 protonated to produce the Δ^1^, β-protonated product that builds up as a stable EP complex. The protonation of EI1 tends to be more rapid than the protonation of EI_2_ throughout MBLs. These explain why carbapenems exhibit a unique tautomer, unlike other β-lactams.^36^

### 4.4. NDM-1 localization and resistance ecology

NDM-1 is synthesized with an N-terminal signal peptide that targets it to the periplasm and has a lipobox. Following signal peptidase II processing, the conserved cysteine undergoes lipidation, fixing the mature enzyme to the outer membrane as a periplasmic lipoprotein. This outer membrane association has been confirmed experimentally by biochemical fractionation and fluorescence microscopy studies of *E. coli* and *Acinetobacter* species, and is unique to NDM-1 and most other β-lactamases, being soluble periplasmic enzymes.^37^ The same research revealed that membrane anchoring is adaptive in host-like environments, such as outer membrane-anchored NDM-1, which is more resistant to zinc deprivation and is catalytically active under stressors simulating nutritional immunity. Mechanistically, zinc sequestration by the host decreases the available Zn(ii) ions that many MBLs require; however, the membrane localization of NDM-1 helps retain the activity and survival during antibiotic exposure.^22,37^ A related aspect of this localization is the encapsulation of active, zinc-loaded NDM-1 into outer membrane vesicles (OMVs). OMVs released by NDM-1 producers harbor enzymatically active NDM-1 and can degrade β-lactams outside the cell, thereby protecting vulnerable bacteria. In a few instances, OMVs also include the NDM gene. This protection and gene transfer *via* OMVs have been shown *in vitro* and in infection models.^22,38^

Beyond protein localization, the genetic ecology of NDM is also important for understanding its spread. The gene is most commonly found in association with the composite transposons Tn125, bounded by two ISAba125, which is regarded as the native mobilization vehicle from *Acinetobacter* spp. to *Enterobacteriaceae* isolates.^39,40^ Subsequent research has described the involvement of IS26 and ISCR1 elements in remodelling the NDM genetic environment, generating variable plasmid topologies that enable worldwide dissemination.^22^ These mobile elements are typically found on broad-host-range plasmids, such as IncF, IncA/C, IncX3, and IncL/M, which transfer efficiently among bacterial species.^41,42^ Notably, these plasmids tend to harbour other resistance determinants that confer co-resistance to aminoglycosides, fluoroquinolones, sulphonamides, and macrolides, making the strains typically categorised as extensively drug-resistant (XDR) or even pan-drug-resistant (PDR).^43–45^

### 4.5. Resistance beyond enzymatic activity

Resistance in NDM-1-producing bacteria is multifactorial and far exceeds enzymatic hydrolysis by the metallo-β-lactamase itself, membrane impermeability, active efflux, and biofilm formation as a community behaviour, each of which provides layers of defense that collectively increase the effective resistance phenotype.^46^ Porins that decrease outer-membrane permeability, notably loss or alteration of the major porins OmpK35 and OmpK36 in *K. pneumoniae*, interact with NDM-1 to produce high carbapenem resistance levels. Reducing the influx of drugs restricts the entry of β-lactam antibiotics into the periplasmic space, and thus, reduces access to the enzyme and its target penicillin-binding proteins (PBPs).^47,48^ Certain deletions or inversions within Loop 3 (L3) of Ompk36 that cause pore constriction have been strongly linked to high carbapenem resistance in clinical NDM-producing isolates and underscore the importance of porin-mediated synergy in resistance.^49^ Efflux pumps also provide a further, frequently additive mechanism of resistance: chromosomally encoded and plasmid-mediated efflux systems reduce intracellular antibiotic concentrations and can cooperate with NDM-1 to diminish susceptibility, even though their action is generally modulatory rather than merely determinative.^50^ In some studies, the overexpression of efflux pumps or the acquisition of efflux determinants in NDM-positive strains indicates that efflux can be a complementary process to enzymatic inactivation, leading to higher levels of resistance.^46^

The development of biofilms further complicates the treatment of NDM-1-producing bacteria, as cells within an extracellular polymeric matrix are less susceptible to antibiotics and are subjected to heterogeneous microenvironments, such as oxygen and nutrient gradients. The conditions facilitate sluggish growth and changes in gene expression, such as stress response and resistance-related pathway suppression.^51^ Critically, biofilm matrices are densely packed with extracellular DNA, which not only provides the biofilm matrix but also enhances the opportunity for horizontal gene transfer, such as conjugation, transformation, and mobilization of mobile genetic elements, thus enhancing the local spread of NDM and co-located resistance genes in mixed communities.^52^ Empirical research and reviews agree that biofilms are hotspots for the exchange of antibiotic resistance genes (ARGs), and thus biofilm-mediated infections by NDM producers are particularly difficult to treat and are likely to disseminate resistance across clinical and environmental niches.^53^

Together, these interrelated resistance mechanisms undermine the effectiveness of existing antibiotic regimens, leading to chronic, difficult-to-treat infections. Resistance is further enhanced by the synergy between enzymatic hydrolysis and auxiliary defence mechanisms, underscoring the limitations of conventional therapies. This led to vigorous research on effective inhibitors against NDM-1 and the resistance pathways.

## 5. Inhibitors against NDM-1

The rapid emergence and global dissemination of NDM-1 have established an imperative for therapeutic approaches to counteract the loss of β-lactam utility against metallo-β-lactamase (MBL)-producing organisms.^54^ In contrast to serine β-lactamases (SBLs) to which approved inhibitors like clavulanate, sulbactam, tazobactam, and, more recently, diazabicyclooctanes are targeted, NDM-1 is a class B MBL dependent on Zn^2+^ catalysis, which is not the case with these SBL-targeting inhibitors.^55,56^ Novel inhibition strategies targeting the structural and mechanistic properties of NDM-1 are urgently needed. Table S2, SI enumerates the inhibitors reported against NDM-1.

### 5.1. Types of NDM-1 inhibitors

#### 5.1.1. Synthetic small molecules as NDM-1 inhibitors

Synthetic small molecules are still the most intensively explored class of NDM-1 inhibitors since medicinal chemistry provides the ability to precisely modulate scaffold geometry, metal binding motifs, and physicochemical characteristics to interact with the enzyme's unique active site. The active site of NDM-1 is solvent accessible and is constructed around a binuclear zinc centre, bridged by a conserved hydroxide or water, and is flanked by flexible loops, especially L3 and L10, and conserved residues such as Trp87, Phe61, and Asn233, which direct substrate binding and provide numerous opportunities for small-molecule interactions.^57^ There are several classes of synthetic small molecules that have been shown to inhibit NDM-1, including thiol-based captopril analogs, hydroxamate-based compounds, and dicarboxylic acids. Moreover, derivatives of boronic acids, and more recently engineered zinc-binding pharmacophores, have been shown to disrupt the binuclear metal centre, or to disrupt substrate coordination.

##### 5.1.1.1. Thiol-based inhibitors

**Fig. 4:**
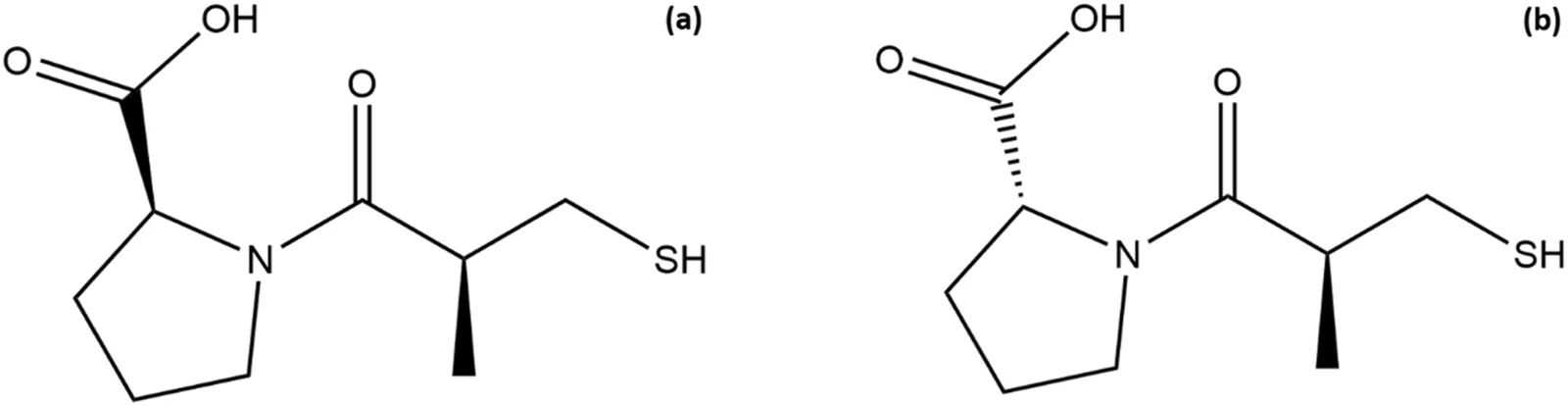
Structures of (a) l-captopril and (b) d-captopril.

Thiol-ligated compounds were among the earliest synthetic inhibitors of NDM-1, since thiols bind zinc tightly and are known as zinc chelators.^13,58^d-Captopril demonstrates a strong inhibitor of NDM-1 with a half maximal inhibitory concentration (IC_50_) value of 7.9 µM, while its stereoisomer l-captopril is much weaker with an approximately 25-fold lower potency and an IC_50_ value of 202.0 µM.^17^ Structural studies by mass spectrometry and X-ray crystallography have solved the NDM-1-l-captopril complex, revealing that the inhibitor's thiol group binds between Zn1 and Zn2 in the active site. This interaction disrupts the bridging water molecules, leading to the competitive inhibition of the enzymes.^59^ Additionally, residue Asn220, which stabilizes reaction intermediates and substrate binding, forms a hydrogen bond with the inhibitor's polar moiety. Meanwhile, the hydrophobic part of the inhibitor interacts with the flexible L3 loop, thereby enhancing the binding stability. The molecular action of captopril against MBLs arises from its essential thiol and carboxylate functional groups, which collectively allow the inhibitor to bind strongly with the zinc ions at the active site of the enzyme. Structural studies have repeatedly demonstrated that both l- and d-stereoisomers of captopril exploit this bimetallic coordination to exhibit extensive inhibitory activity against B1 MBL subclasses, including NDM-1, IMP-1, VIM-2, and BcII, and the findings were supported by several high-resolution crystal structures and biochemical analyses^57^ (Fig. 4).

**Fig. 5:**
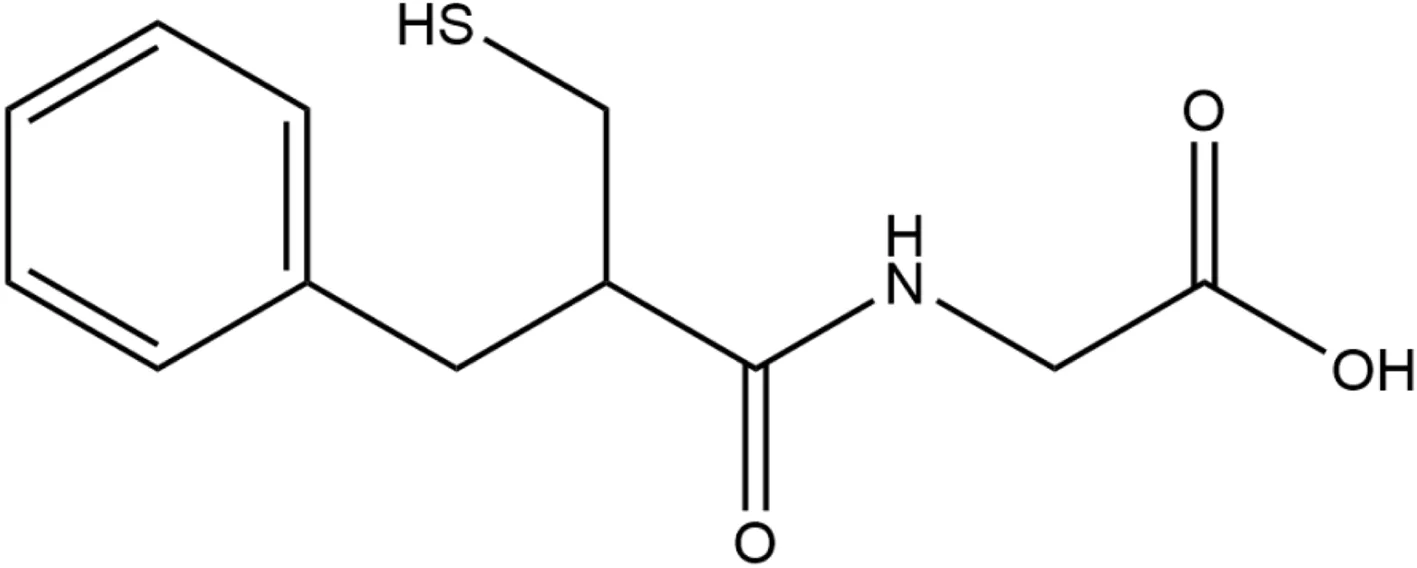
Chemical structure of thiorphan.

Captopril is a fundamental scaffold in the development of thiol- and carboxylate-based inhibitors of NDM-1. The molecule consists of two structural elements, a 3-mercapto-2-methylpropanoyl group and a proline unit, and plays an important role in determining the active pharmacophores for metallo-β-lactamase inhibition.^15^ Klingler and coauthors developed a rational method for the design of metallo-β-lactamase (MBL) inhibitors by synthesising a set of d-captopril analogues, resulting from modifications to proline residues and the 3-mercapto-2-methylpropanoyl fragment.^13^ This method not only furnished a rationale design strategy but also a platform for clinically used thiol-containing drugs. Three FDA-approved compounds with appreciable inhibitory activity in the low micromolar range were surprisingly found against NDM-1. Thiorphan, which was initially synthesized as an inhibitor of enkephalins, showed an IC_50_ of 1.8 µM,^60,61^ while dimercaprol, a classical antidote for heavy metal poisoning, was found to be the most effective with an IC_50_ value of 1.3 µM (Fig. 5).

**Fig. 6:**
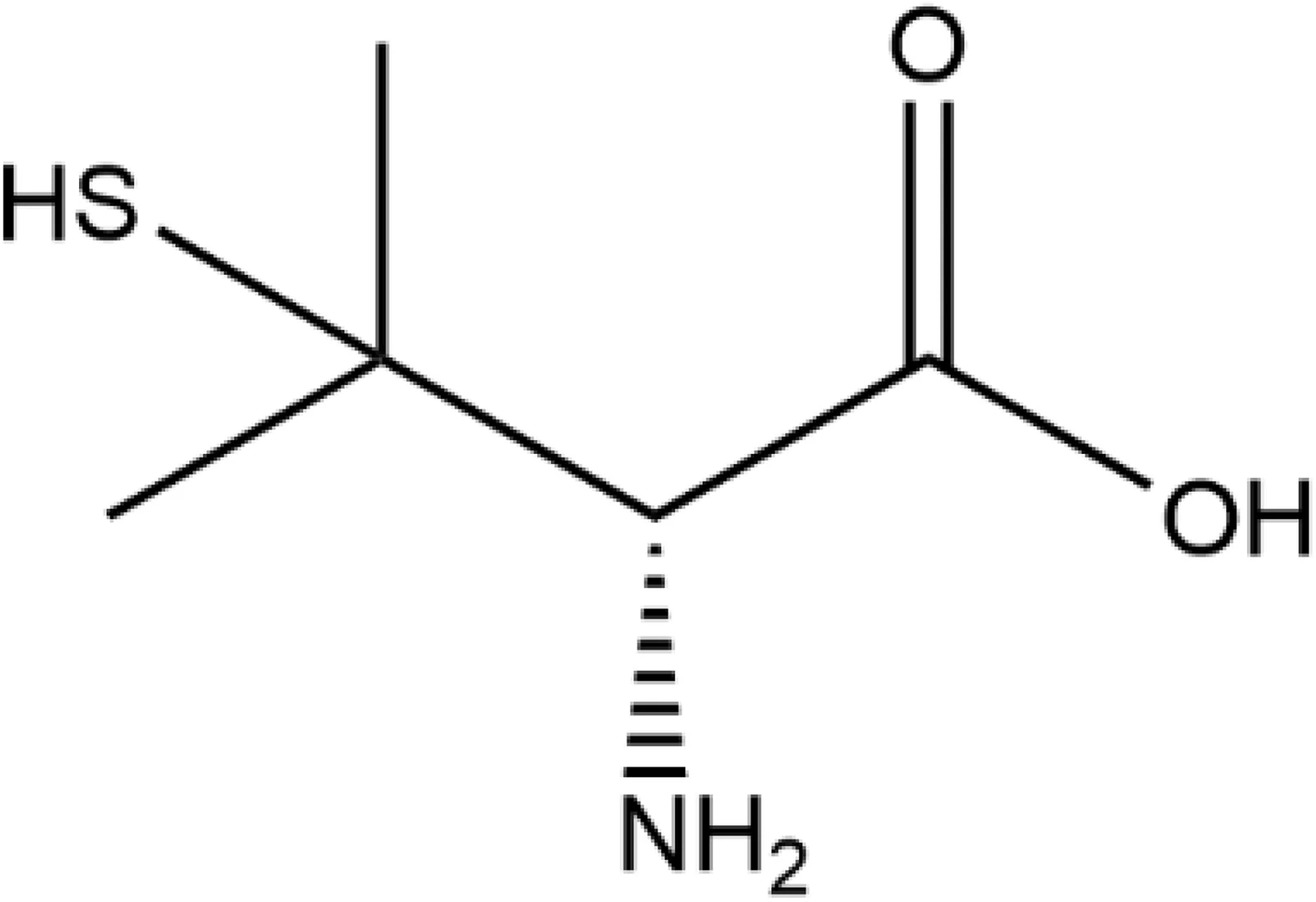
Chemical structure of penicillamine.

Penicillamine, a drug used for the treatment of Wilson's disease, also showed considerable inhibitory action. Among these, other thiol-based scaffolds, such as thiomandelic acid and 2-mercapto-3-phenyl propionic acid, were also found to effectively restore meropenem activity against NDM-1 producers, further emphasizing the clinical significance of thiol pharmacophores in overcoming resistance^62^ (Fig. 6).

Tiopronin showed poor inhibition (IC_50_ = 84 µM), while *N*-acetylcysteine was inactive, suggesting that effective NDM-1 inhibition requires specific structural features.^13^ Although thiol-based inhibitors have high biochemical potential, they face significant translational constraints. Their reactive nature tends to cause non-specific binding to off-target metalloproteins and intracellular thiols, such as glutathione, thereby decreasing selectivity. Moreover, the oxidation of thiol groups is prone to occur under physiological conditions, further reducing their stability and undermining their clinical development.^63^ These translational hurdles are closely linked to the cell's intrinsic redox biology. Eukaryotic cells experience a tightly controlled redox state vital to normal metabolism and signalling. Under physiological conditions, the cytosol is highly reducing due to elevated intracellular glutathione (GSH) levels and the presence of glutathione reductase and other redox enzymes that maintain GSH in its reduced form. This type of environment ensures that most protein cysteine thiols are reduced and active, but some non-exposed cysteine residues can stabilize *via* disulfide bonds. When oxidants increase moderately, such as in response to cytokines, growth factors, or thiol-disulfide exchange reactions, certain proteins become reversibly modified by thiol modifications. These modifications act as molecular switches, altering protein shape and function to control cell signalling. Thiols are first converted into sulfenic acids, which are intermediates that can further form disulfides or glutathionate with glutathione through a process termed glutathiolation. These alternations serve to regulate enzymes and structural proteins that participate in metabolism and signal transduction. However, if oxidative stress is excessive, thiol groups irreversibly oxidize, glutathione reserves are depleted, and proteins are inactivated. This damage inhibits normal cell function and initiates cell death.^64^

##### 5.1.1.2. Hydroxamates and spiro-scaffolds

Hydroxamates are among the most promising scaffolds for inhibiting NDM-1, due to their aggressive bidentate chelation with Zn(ii) ions at the active site. In contrast to monodentate thiols, hydroxamates coordinate with Zn1 and Zn2 in a bridging or chelating manner and thus potently interfere with catalysis. *In vitro* assays showed that typical hydroxamate derivatives reduced the MIC of meropenem by 4- to 16-fold against strains producing NDM-1, highlighting their potential to desensitize bacteria to carbapenems. Structural analysis also demonstrated that the hydroxamate group coordinates with Zn(ii) in a tetrahedral geometry, while the attached aromatic groups exploit hydrophobic pockets near dynamic loops (L3 and L10) to enhance the binding stability.^65^

**Fig. 7:**
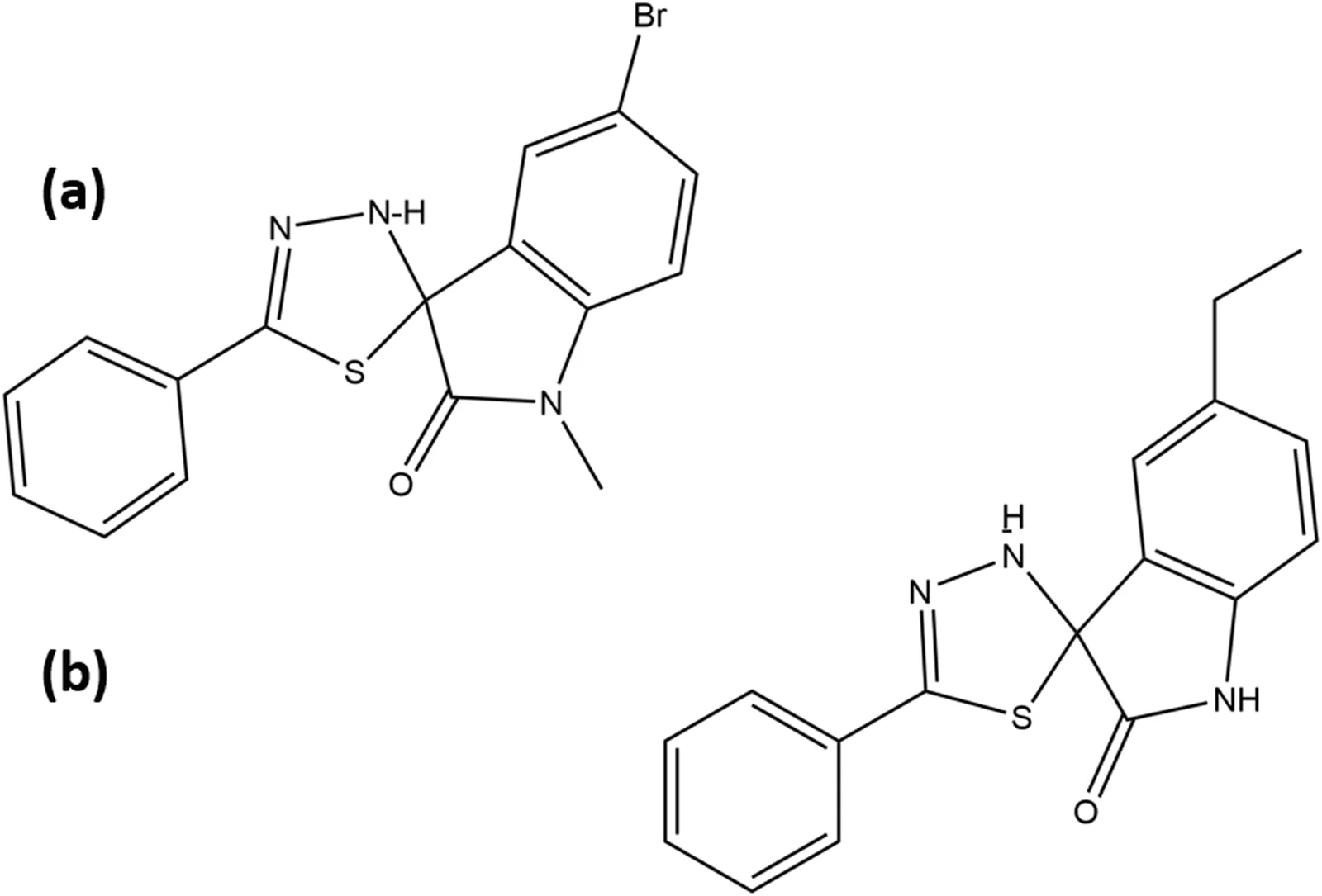
Structure of the thiadiazole derivatives (a) 5-bromo-1-methyl-5′-phenyl-3′*H*-spiro[indoline-3,2′-[1,3,4]thiadiazol]-2) (b) (5-ethyl-5′-phenyl-3′*H*-spiro[indoline-3,2′-[1,3,4]thiadiazol]-2 (adapted from Li *et al.*, 2021,^15^ licensed under CC BY 4.0).

In addition to hydroxamates, spiro-indoline-thiadiazole (SIT) scaffolds have proven effective as metal-chelating molecular scaffolds. This family was initially reported by Falconer *et al.*, highlighting that sulfur in the thiadiazole backbone was crucial for both zinc chelation and bacteriostatic action, leading to the conservation of this structural core across all analogues^66^ (Fig. 7).

Early derivatives of spiro-indoline-thiadiazole (SIT) like SIT-1, SIT-2, and SIT-3 demonstrated the ability to coordinate with zinc, while optimizing the R1 and R3 positions yielded more effective analogues such as SIT-Z1 and SIT-Z5. Among these, SIT-Z5 showed the greatest inhibitory profile with an IC_50_ value of 6.6 ± 1.3 µM, a minimum inhibitory concentration (MIC) of 4 µM and a high level of synergy with meropenem against clinical isolates of *K. pneumoniae* carrying NDM-1. It is important to note that SIT-Z5 decreased the MIC of meropenem from >2 µM to 0.25 µM and increased the activity of other carbapenems, such as doripenem and biapenem. Mechanistic studies found that SIT-Z5 cleaves the S–C spiro bond and opens the thiadiazole ring to form an imine-based ligand that exposes sulfur and nitrogen atoms for coordination to Zn(ii) in the active site, thereby inhibiting enzymatic activity.^66,67^ Although these results are encouraging, spiro-indoline-thiadiazoles have problems translating to the levels of other metal-chelating inhibitors. Their complexity can restrict their solubility and bioavailability, and the possible instability of the thiadiazole ring under physiological conditions can limit their systemic use. Moreover, non-selective binding to host metalloproteins is a concern with respect to off target toxicity. Thus, even though hydroxomates and spiro-scaffolds are promising targets against NDM-1, additional optimization of pharmacokinetic and ADME characteristics is necessary for clinical development.

##### 5.1.1.3. Boronic acids and bicyclic boronates

Boronic acid derivatives have become one of the clinically most developed classes of β-lactamase inhibitors due to their ability to effectively imitate the tetrahedral transition state of β-lactam hydrolysis. The boron atom in boronic acid is sp^3^-hybridized at physiological pH, enabling such compounds to mimic the high-energy tetrahedral intermediate that results during enzymatic cleavage of β-lactam antibiotics. Notably, cyclic boronates were the first dual-action inhibitors reported to inhibit both serine β-lactamases (SBLs) and zinc-dependent MBLs.

**Fig. 8:**
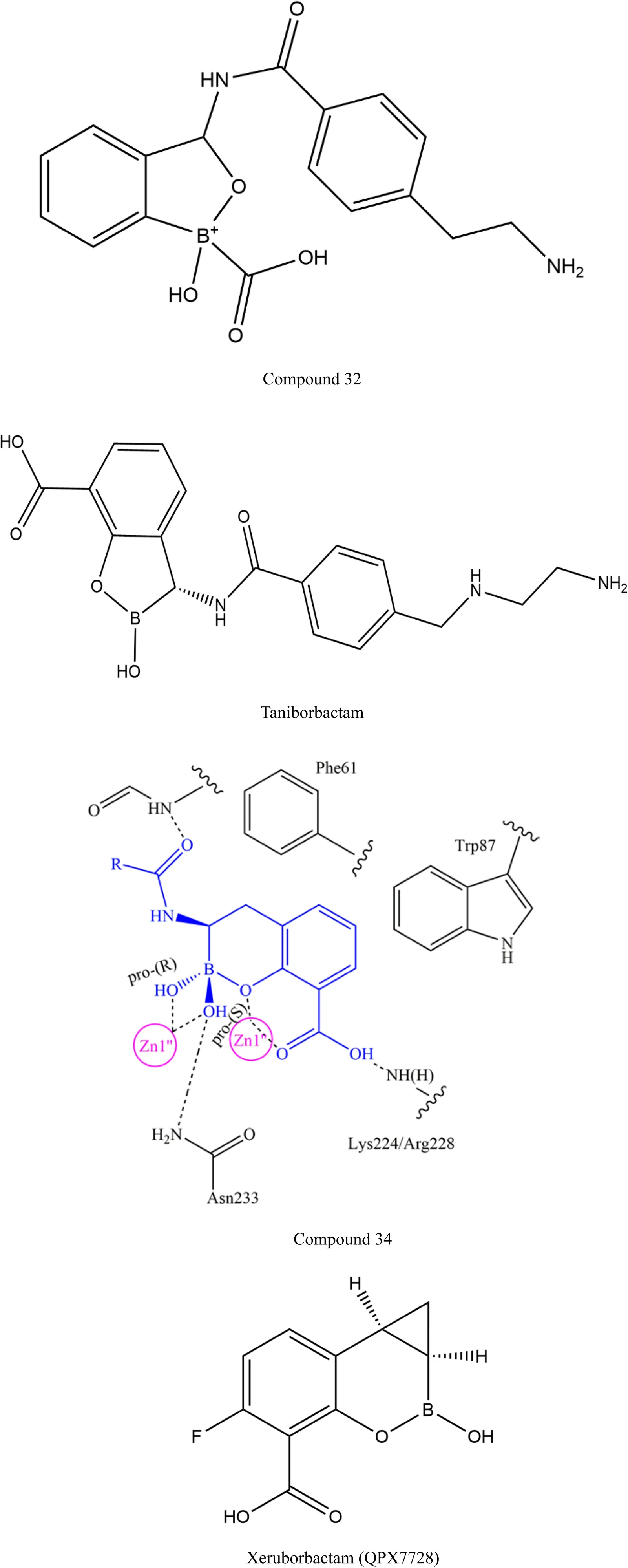
Depiction of some derivatives of boronic acid [compound 32, taniborbactam, compound 34, and xeruborbactam] (adapted from [Li *et al.*,^15^ 2021], licensed under CC BY 4.0).

Biochemical analysis showed that various bicyclic boronate derivatives have sub-micromolar to nanomolar inhibitory activity against NDM-1 and other MBLs (Fig. 8). Moreover, bicyclic boronate-based inhibitors, such as representative and clinically advanced agents like taniborbactam and compound “32”, have been shown to have strong synergistic activity in combination with meropenem, lowering the MICs of the NDM-1 producing strains by up to 64-fold, which supports the resensitizing potential of bicyclic boronate-based compounds.^68^ Structural analyses have provided insights into their mechanism of action. As an example, X-ray crystal structure of NDM-1 in complex with compound “34” demonstrated that the C-3 carboxylate group is bound by Zn^2+^ and interacts with the conserved residues Trp87 and Phe61 with the bicyclic phenyl-boronate core. Furthermore, the two exocyclic boron hydroxyl groups directly coordinate to Zn1 and hydrogen bonds to Asn233 and the NH of the acetylamino side chain. These bindings reflect the complex binding mode of bicyclic boronates, which exhibit strong synergistic metal coordination and stabilizing interactions with conserved residues in the active site.^68^ Among the most clinically promising inhibitors in this class is taniborbactam, a bicyclic boronate that exhibits strong inhibition of SBLs and MBLs, including NDM-1 and VIM-2. Taniborbactam reactivates cefepime and meropenem against multidrug-resistant *Enterobacterales* and *P. aeruginosa in vitro* and *in vivo*.^69^ Crystallographic studies established that taniborbactam occupies the NDM-1 active site by coordinating the two Zn^2+^ ions and interacting with conserved residues Trp87, Phe61, Lys224, and Asn233 to stabilize the tetrahedral boronate intermediates.^8^ Another remarkable cyclic boronate under development is xeruborbactam (QPX7728). Optimized through the scaffold design of the bicyclic structure, xeruborbactam displays ultrabroad-spectrum activity against NDM variants, with good Zn(ii) binding. In contrast to the previous boronate inhibitors, xeruborbactam has also been found to possess intrinsic antibacterial activity, at least in part due to its rigid, compact scaffold, allowing it to bind diverse β-lactamase active sites. Notably, it exhibits decreased susceptibility to efflux and porin mutations, which are prevalent resistance mechanisms among Gram-negative pathogens.^70^

**Fig. 9:**
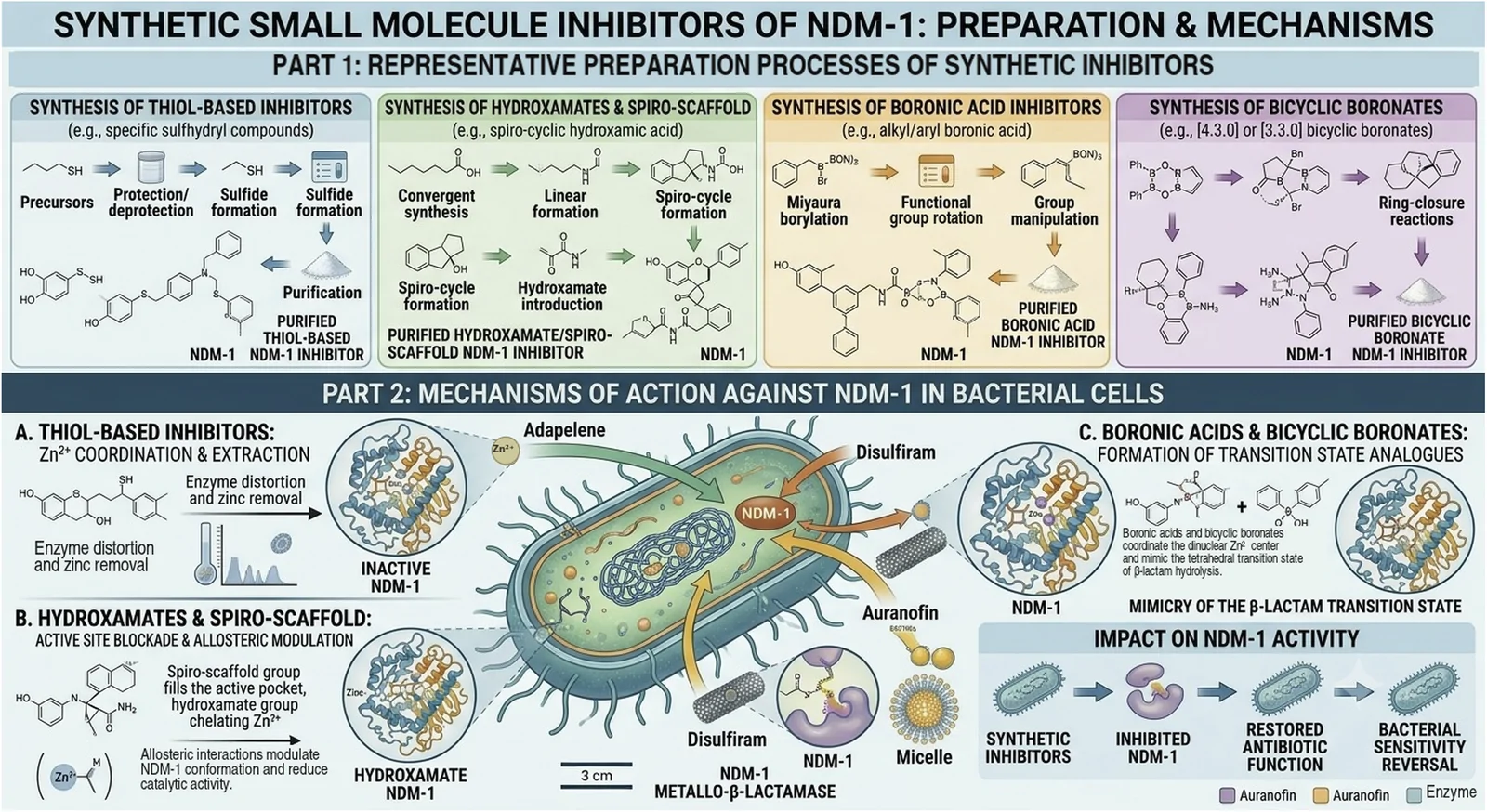
Detailed depiction of the preparation and mechanisms of the small molecule-based inhibitors of NDM-1: Part 1: representative preparation process of synthetic inhibitors; Part 2: mechanisms of action against NDM-1 in bacterial cells (A) thiol-based inhibitors (B) hydroxamates & spiro-scaffold, (C) boronic acida & bicyclic boronates (image generated using Adobe Firefly Image v3).

Recent research evaluating the efficacy of cefepime-taniborbactam against recombinant *Escherichia coli* (*E. coli*) strains expressing various NDM variants underscores the inhibitory problem caused by allelic diversity. While isolates expressing NDM-1, NDM-5, and NDM-7 were completely susceptible to the drug combination, the strain expressing NDM-9 was significantly resistant. This variant is distinguished from NDM-1 by a single point mutation, Glu152Lys, within the omega loop (between helix α4 and strand β8), an area important for determining the active-site conformation. Such a minor modification is sufficient to disrupt taniborbactam's inhibitory activity, highlighting how modest structural changes can undermine inhibitor binding and efficacy.^71^Fig. 9 illustrates the small molecule-based inhibitors of NDM-1: their preparation and mechanisms.

#### 5.1.2. Drug repurposing strategies for NDM-1 inhibitors

Repurposing drugs have been recognized as an economic and time-saving approach to discover new therapeutics against multidrug-resistant pathogens, especially those that produce New Delhi metallo-β-lactamase-1 (NDM-1). The urgent demand for effective NDM-1 inhibitors and the long timelines required for new compounds, repurposing FDA-approved compounds, or clinically assessed molecules with established safety and pharmacokinetic profiles presents a distinct advantage. This strategy enables researchers to avoid initial-stage toxicology assessments, accelerate clinical translation, and control structural homology between host metalloenzymes and bacterial MBLs. A number of drugs initially designed for other indications, *e.g.*, inflammation, alcoholism, or rheumatoid arthritis, have shown ineffective *in vitro* inhibition of NDM-1 and, in some instances, *in vivo* activity in infection models.

##### 5.1.2.1. Adapalene

Adapalene was discovered through a structure-based drug repurposing approach that included virtual screening, molecular docking, and molecular dynamics (MD) simulations, but its inhibitory activity against NDM-1 is not limited to this approach. The study by Shailaja *et al.*^72^ used several experimental methods to support the computational results and make the strongest possible case for the use of adapalene as an NDM-1 inhibitor, unlike most early computational screening studies based on predicted binding affinities. An integrated workflow included virtual screening of the FDA-approved drug library, molecular docking, molecular dynamics simulations, recombinant NDM-1 enzyme inhibition assays, and microbiological susceptibility testing of clinically relevant bacterial isolates harbouring NDM-1. This strategy of rational drug repurposing, using computational and experimental techniques, is a promising approach for identifying inhibitors that can rapidly restore carbapenem activity against multidrug-resistant pathogens.

Adapalene was one of the top compounds identified in the computational screening as inhibitors of the catalytic pocket of NDM-1. The molecular docking predicted a binding affinity of −9.21 kcal mol^−1^, which showed a favourable interaction with the enzyme. The detailed structural analysis revealed that the catalytic cavity has multiple hydrophobic and hydrogen-bond interactions with the catalytically important residues, such as His122, Asp124, His189, Cys208, and His250, which are crucial to the structural integrity and catalytic activity of the di-zinc active site. Perhaps, most importantly, the binding mode proposed that adapalene could sterically block the substrate-access pathway and also stabilize interactions within the active site. The authors then calculated the root mean square deviation (RMSD) and root mean square fluctuation (RMSF) for the protein–ligand complex to assess the interaction's stability under dynamic physiological conditions. Furthermore, 100 ns molecular dynamics simulations reveal little structural deviation in the protein–ligand complex. These results suggested that adapalene was well bound in the catalytic pocket throughout the simulation, and hence, the predicted docking pose is not a computational artifact but rather a biologically relevant binding mode.^72^

Most significantly, the computational predictions were validated by using purified recombinant NDM-1 protein in experiments. The ability to inhibit NDM-1 hydrolytic activity was confirmed using enzyme inhibition assays, in which adapalene demonstrated high inhibition. This enzymatic validation is important as there are no clear associations between favourable docking scores and functional inhibition, especially in this context. The study showed decreased catalytic activity of purified NDM-1 in the presence of adapalene, indicating that the compound not only has favorable theoretical binding characteristics but also affects the enzyme's catalytic activity. Therefore, adapalene should not be considered solely as a molecule derived from computer-aided design, but rather as a validated lead compound to be developed in pharmacology.

The microbiological studies were also consistent with the results of the adapalene's benefit. The checkerboard broth microdilution assays showed that adapalene significantly increased the antibacterial activity of meropenem against clinical isolates of NDM-1-producing *E. coli* and *K. pneumoniae*. This combination resulted in a synergistic effect with the carbapenem antibiotic, significantly lowering the MIC of meropenem compared with treatment with the antibiotic alone. However, it is important that adapalene itself has little antibacterial activity, meaning it is unlikely to kill bacterial cells; rather, its mode of action is the suppression of the resistance enzyme. This is therapeutically beneficial, since the β-lactamase inhibitors allow existing antibiotics to retain their activity while not exerting as much selective pressure for resistance development as conventional antimicrobial drugs.^72^

**Fig. 10:**
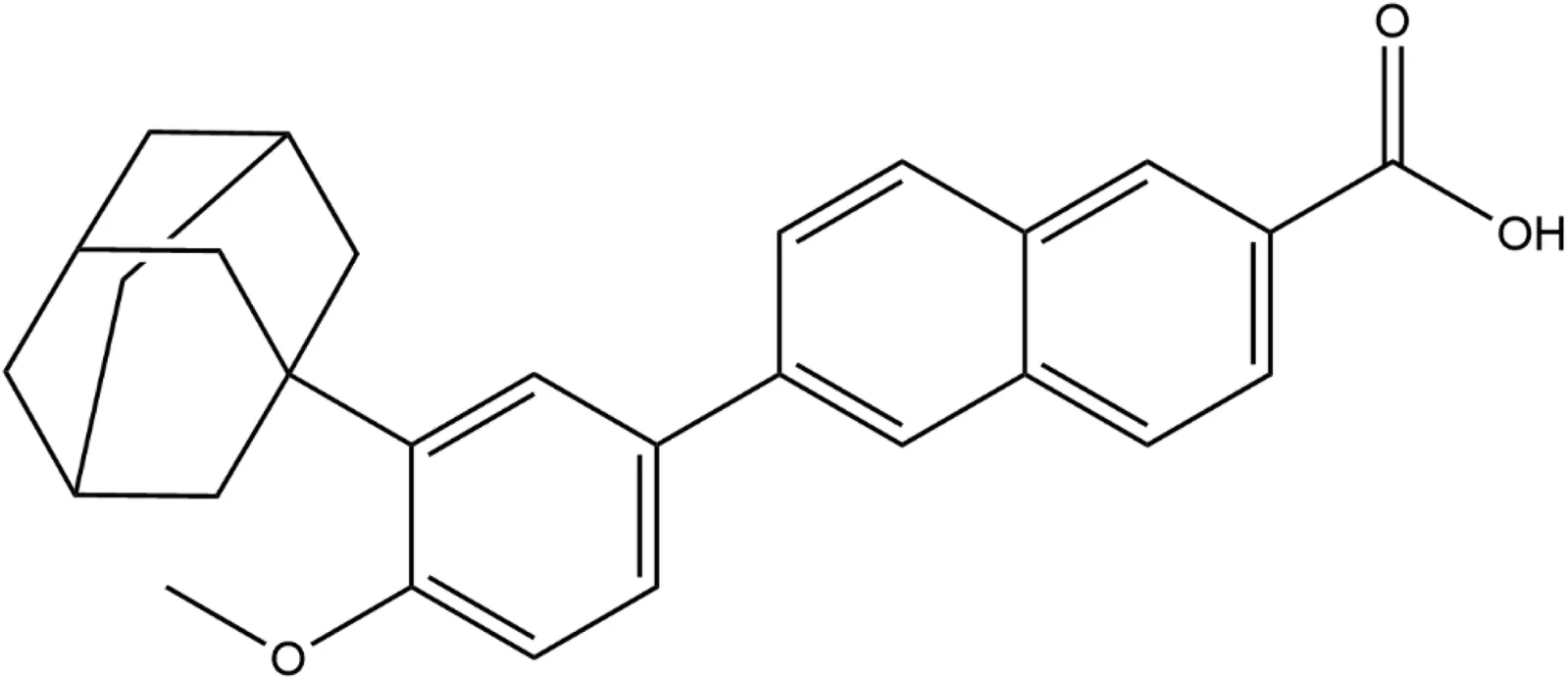
Structure of adapalene containing an adamantane ring, a methoxy-substituted phenyl ring and a naphthoic acid moiety.

Although these promising results have been achieved, to date there is only limited evidence of adapalene's efficacy, based on *in vitro* biochemical and microbiological studies. Adapalene has not been evaluated in extensive animal models of bacterial infection caused by NDM-1, although several β-lactamase inhibitors have reached advanced preclinical stages. It is therefore not certain that the beneficial enzyme inhibition and synergistic antimicrobial effect observed *in vitro* will be maintained *in vivo*, due to variable plasma protein binding, tissue distribution, metabolic stability, immune response, and bacterial fitness. In addition, to date, no pharmacokinetic or pharmacodynamic studies have been reported to assess systemic availability following administration, allowing determination of whether adequate systemic levels to inhibit NDM-1 can be safely achieved. Another factor is that adapalene is a third-generation topical retinoid initially developed for the treatment of acne vulgaris^73^ (Fig. 10). The clinical safety profile is well established and attractive for drug repurposing, but systemic administration may have pharmacologic and toxicologic properties different from those of topical application. Among the unknown parameters that impact antimicrobial therapy are the drug's oral bioavailability, metabolic stability, tissue penetration, elimination half-life, and potential off-target interactions. Therefore, further formulation optimization and/or structural modification may be needed before adapalene can be used as a clinically viable carbapenem adjuvant.

Overall, adapalene is a significant case study of the effective use of computational drug repurposing to find experimentally validated inhibitors of NDM-1. The study by Shailaja *et al.*^72^ is far more than *in silico* screening and includes enzyme inhibition and microbiological synergy experiments, thus providing proof of concept that adapalene can restore carbapenem activity against NDM-1-producing pathogens in the laboratory. However, there is no pharmacokinetic evaluation, toxicity studies, structural validation by crystallography, or *in vivo* efficacy studies, suggesting that, at the moment, adapalene cannot be considered a clinically validated NDM-1 inhibitor, but a promising preclinical lead. Future studies combining structural biology, medicinal chemistry, pharmacokinetic optimization, and animal infection models will be crucial to determine whether adapalene can be advanced into clinical development as a potential effective adjunct for combating carbapenem-resistant *Enterobacteriaceae* (CRE).

##### 5.1.2.2. Ebselen

**Fig. 11:**
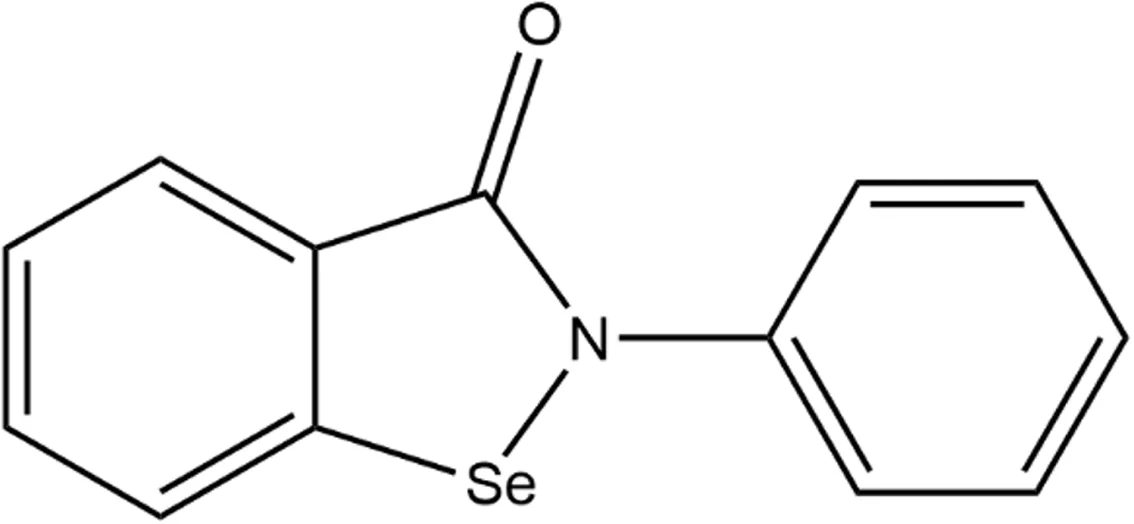
Structure of ebselen containing a benzisoselenazolone ring fused with a lactam moiety substituted with a phenyl ring.

Among repurposed candidates for NDM-1 inhibition, ebselen has emerged as a notable chemical entity owing to its dual mechanism of action and generally favourable safety profile (Fig. 11). Ebselen has reached human clinical trials stage as a therapeutic agent for diseases like cerebral ischemia and stroke. Preclinical experiments show it is well-tolerated, with low toxicity and good safety in animal models.^74^ Initially, the molecule ebselen was discovered for its antioxidant and anti-inflammatory effects; however, it was then shown to be inhibiting the NDM-1 enzyme by binding to the enzyme's binuclear zinc center. The selenium atom can be observed to be covalently bonded with the catalytic cysteine residues (Cys221), displacing one of the zinc ions. Mass spectrometry and kinetic assays have validated this distinctive mechanism, evidencing time- and concentration-dependent inhibition of NDM-1. Ebselen also reduces the MIC values of β-lactams against NDM-1-producing *E. coli* up to 128-fold for meropenem and 16-fold for ampicillin, thus reviving the activity of these antibiotics against resistant strains.^75^ The inherent reactivity of ebselen, however, limits its therapeutic use because the selenium group may covalently react with a wide range of thiol-containing biomolecules, such as glutathione and cysteine residues in host proteins, thereby disrupting the target specificity. Moreover, its redox activity can result in pleiotropic biological effects, making its development as a specific metallo-β-lactamase inhibitor difficult. Moreover, the presence of the selenium group poses potential toxicity issues that limit its potential for further drug development.^75,76^

##### 5.1.2.3. Disulfiram

**Fig. 12:**
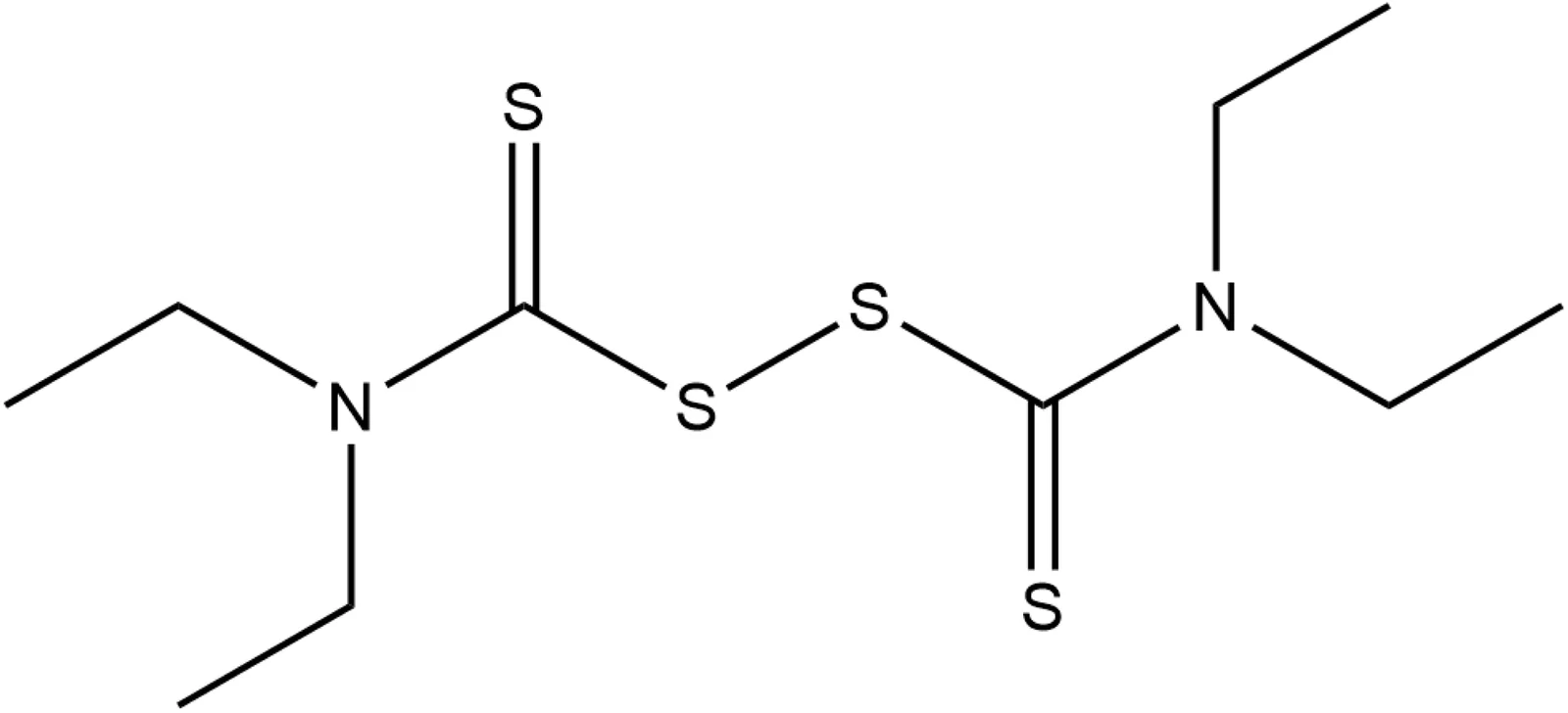
Disulfiram consisting of three major structural components: a central disulfide bond (–S–S–), thiocarbamoyl (thiuram) groups and diethylamino substituents.

Disulfiram, a well-established oral agent for alcoholism, is metabolized to derivatives such as diethyldithiocarbamate (DDTC) and diethylamine, which have been studied to inhibit aldehyde dehydrogenase and result in acetaldehyde accumulation during ethanol metabolism^77^ (Fig. 12). Beyond its traditional application, disulfiram has been investigated for pleiotropic pharmacological actions that may involve anti-obesity,^78^ anticancer,^79^ and antimicrobial properties.^80^

Disulfiram inhibits NDM-1 by establishing a covalent bond with the active-site catalytic residue Cys208, which also causes one Zn(ii) to be displaced from the active site of the enzyme. One of its principal *in vivo* metabolites, Cu (DTC)_2_, a diethyldithiocarbamate-derived copper complex, has been reported to exhibit strong, broad-spectrum inhibition of both the B1 and B2 subfamilies of metallo-β-lactamases. This reaction proceeds *via* a unique mechanism that involves the oxidation of the Zn(ii)-thiolate centre of the compound, thereby inactivating the enzymatic activity. Disulfiram analogues were shown to covalently inactivate NDM-1, presumably by forming a disulfide (S–S) linkage with the thiol group of Cys208, thereby facilitating the release of one Zn(ii) ion from the catalytic site.^80^ To verify this interaction, scientists built a Cys208Ala site-directed mutant of NDM-1 and compared its action with that of the wild-type enzyme. The displacement of Cys208 in biochemical assays resulted in a roughly 75% decrease in enzyme activity, indicating that the residue is essential for optimal catalytic activity. Additionally, when challenged with disulfiram (DSF) or Cu (DTC)_2_, its copper-containing metabolite at 10 µM, wild-type NDM-1's inhibitory effect was not affected in the NDM-1 Cys208Ala mutant or in the subclass B3 MBL, which naturally lacks a cysteine residue in its active site. These results give strong evidence that Cys208 is the major residue responsible for disulfiram-driven inhibition of NDM-1.^80^ Disulfiram has been shown to exhibit a direct antibacterial activity against *Staphylococcus aureus* (*S. aureus*), with minimum inhibitory concentrations (MICs) of 8–16 µg mL^−1^, an ability to inhibit biofilm growth, and efficacy against intracellular *S. aureus*.^81^ Krajaejun *et al.* demonstrated that disulfiram inhibits urease and aldehyde dehydrogenase and suppresses the growth of *Pythium insidiosum* (a pathogenic oomycete) *in vitro*.^82^

Mechanistic work has also determined that disulfiram's antibacterial activity is partly due to zinc-dependent reactive oxygen species (ROS) induction.^83^ In *E. coli*, treatments with disulfiram resulted in intracellular ROS accumulation in a zinc-dependent manner; supplementation with ROS scavengers or zinc chelators suppressed bacterial growth inhibition, verifying the interaction between metal binding and oxidative stress disruption.^83^

Disulfiram is a potential candidate for repurposing a clinically accepted drug to combat NDM-1-mediated resistance. Its potent synergistic action with carbapenems, shown in both *in vitro* and *in vivo* infection models, forms a strong platform for clinical translation. Nevertheless, more work needs to be done to overcome its drawbacks, such as the use of disulfiram, which has been associated with a broad range of side effects, from reasonably minor symptoms to severe, even life-threatening complications. More common but usually milder reactions consist of headache, tiredness, drowsiness, and impotence. These are often temporary and reversible, and rarely require medical attention. One of the main issues is when patients drink alcohol during disulfiram treatment. As the drug inhibits acetaldehyde dehydrogenase, acetaldehyde builds up in the blood, causing the disulfiram-ethanol reaction. This usually develops within 12 hours of dosing and is characterized by facial flushing, sweating, tachycardia, nausea, and vomiting. In more severe instances, the reaction could escalate to hypotension, respiratory depression, cardiac arrhythmias, seizures, cardiovascular collapse, or death.^84^

##### 5.1.2.4. Auranofin

**Fig. 13:**
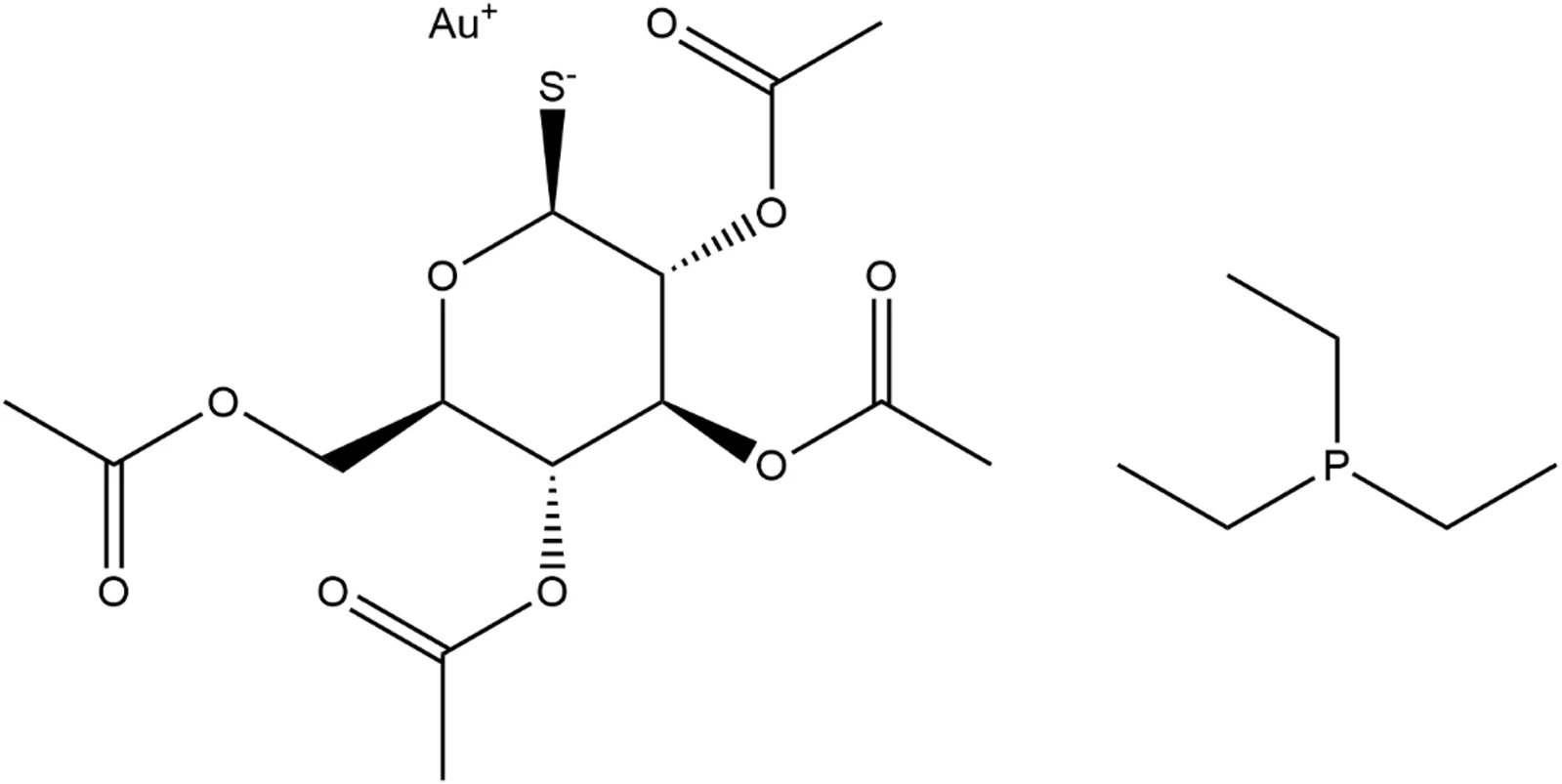
Structural depiction of auranofin showing the gold(i) centre coordinated to a triethylphosphine ligand and a tetra-acetylated thioglucose ligand.

Auranofin, a gold(i)-containing antirheumatic pharmaceutical initially approved by the US Food and Drug Administration (FDA) in 1985 for the treatment of rheumatoid arthritis, has recently surfaced as a potential repurposed agent to fight β-lactam resistance induced by New Delhi metallo-β-lactamase-1 (NDM-1) and similar enzymes^85^ (Fig. 13).

Auranofin, chemically known as 1-thio-β-d-glucopyranosatotriethylphosphine gold-2,3,4,6-tetraacetate, is a prodrug that releases the active [Au(PEt_3_)]^+^ cation upon cleavage of its thiosugar functional group in biological systems.^86^ The cation has a high affinity for thiol and selenium residues, enabling it to make covalent Au–S or Au–Se linkages in metalloproteins.^87^ The mechanistic use of this property has refocused attention on auranofin as a dual inhibitor of zinc-dependent resistance determinants, specifically MBLs, such as NDM-1, and mobilized colistin resistance enzymes (MCRs), both of which rely crucially on Zn(ii) cofactors for catalytic activity. NDM-1 has a di-zinc catalytic centre, with two Zn(ii) ions connected by a hydroxyl group that is critical for hydrolytic β-lactam ring cleavage. Crystallographic analyses have established that exposure to auranofin results in the removal of both Zn(ii) ions from the active site and their replacement with Au(i) ions, forming a stable Au-NDM-1 adduct.^87^ The resultant crystal structure (PDB ID: 6LHE), determined at 1.20 Å, showed two gold atoms, Au282 and Au283, occupying the conventional Zn1 and Zn2 sites. The ions have a quasi-tetrahedral coordination geometry and interact with the critical residues in a respective manner, including Cys208, Asp124, His122, His189, His250, and water molecules in the catalytic pocket. The Au–S and Au–N bond lengths (1.9–2.5 Å) are within the range of native Zn(ii) coordination, but the net metal–metal distance (∼3.8 Å) is less than the usual Zn–Zn separation (∼4.6 Å), indicating a more compact and electronically differentiated configuration.^87^

This pseudo-tetrahedral structure was further confirmed by density functional theory (DFT) calculations by Tolbatov and Marrone (2023), who put forward that the Au–Au bimetallic center may be in either Au(i)–Au(i) or Au(ii)–Au(ii) states based on the redox conditions.^85^ Their computational simulations suggested that the Au(i)–Au(i) system is most likely an early intermediate after Zn/Au exchange, which can be partially oxidized to give an Au(ii)–Au(ii) arrangement with the greatest structural resemblance to the experimental X-ray information. Such an electronic transition is likely to cause irreversible inhibition of NDM-1 activity, as the substitution interferes with the coordination of the nucleophilic hydroxide required for β-lactam hydrolysis. In addition, covalent interaction between [Au(PEt_3_)]^+^ and the thiol side chain of Cys208 is essential for stabilizing the Au-bound complex, as evidenced by mutagenesis studies, indicating that the substitution of Cys208 with alanine significantly impairs auranofin-mediated inhibition.^87^ One unresolved key issue is the Zn/Au exchange reaction, specifically the steps controlling the decomplexation of the well-bound triethylphosphine (Pet_3_) ligand from the active [AuPEt_3_]^+^ species. While the common assumption is that the activation of auranofin produces the cationic [AuPEt_3_]^+^ complex, the specific sequence of chemical events that lead to its interaction with metalloenzymes is not yet fully defined.^88^ One of the hypotheses accepted by most is that gold's high affinity for cysteine and selenocysteine residues makes ligand substitution possible, either by reacting with enzyme-active site thiols of proteins or free thiols found in the cytoplasm.^89^ This has given rise to an ongoing controversy over whether auranofin first undergoes ligand exchange with its molecular target or whether activation is only possible upon binding at the enzyme's active site. As such, experimentally defining the chemical events triggered by auranofin administration remains extremely problematic, primarily because of the significant chemical complexity introduced by this metallodrug during intracellular uptake and after binding to target sites.^86,90,91^ The molecular details of Zn/Au exchange in NDM-1 thus remain unresolved and are the subject of ongoing theoretical mechanistic research.

**Fig. 14:**
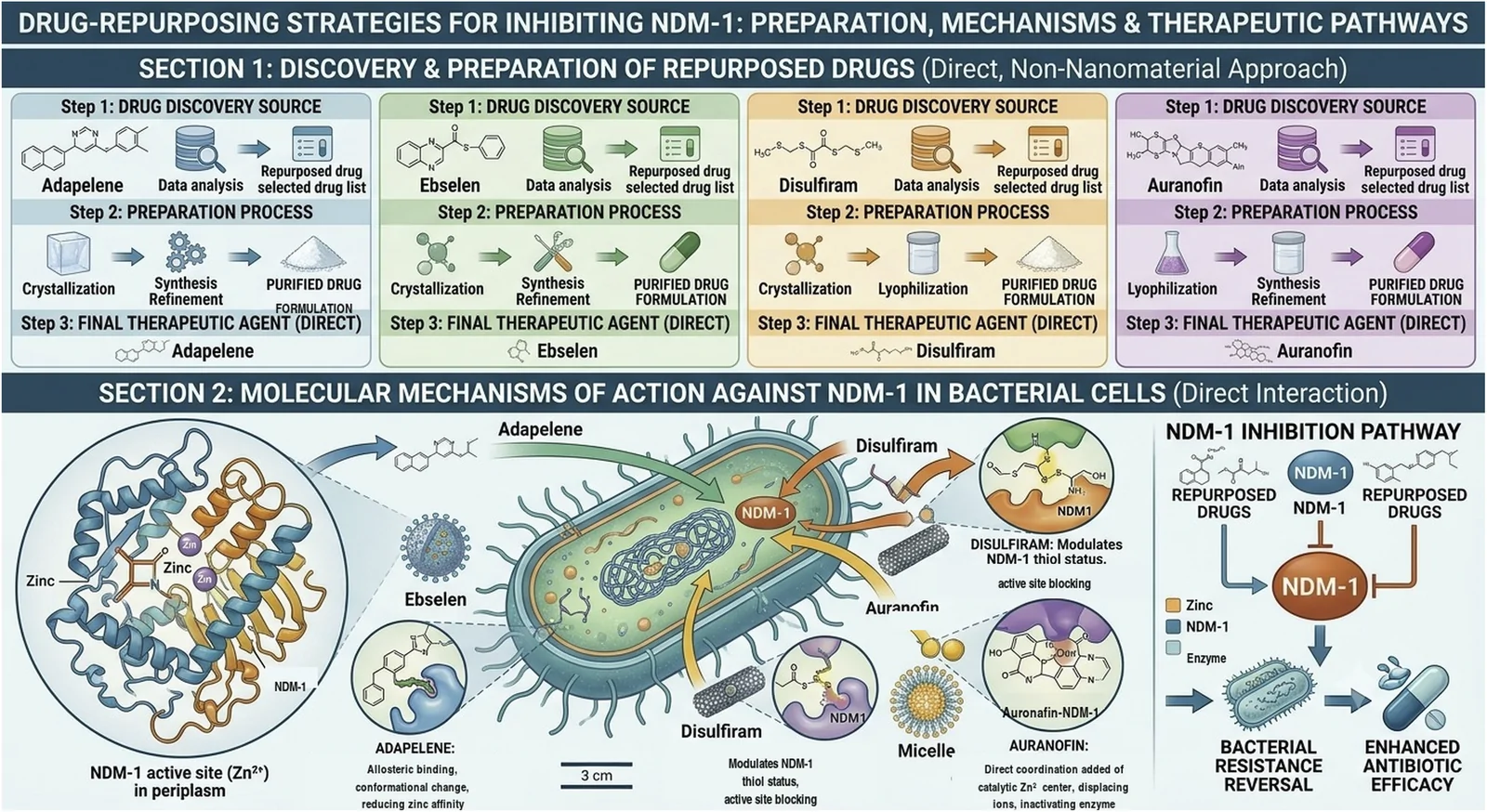
Schematic of drug repurposing approaches: Section 1: discovery & preparation of repurposed drugs; Section 2: molecular mechanisms of actions against NDM-1 in bacterial cells; (image generated using Adobe Firefly Image v3).

Synthetic small molecules and repurposed drugs have shown considerable promise against NDM-1, but many candidates still face challenges with toxicity, off-target metal chelation, poor pharmacokinetic properties, and limited efficacy against emerging NDM variants (Fig. 14). These limitations have encouraged researchers to look for other sources of inhibitors that have better selectivity and safety profiles. In this context, natural products have emerged as a popular source of structurally diverse bioactive compounds that can interact with NDM-1 through various mechanisms including direct active-site binding, allosteric modulation and disruption of catalytic function. Therefore, more focus has been given to plant- and microbe-derived metabolites as potential adjuvants for restoring the activity of beta-lactam antibiotics against NDM-producing pathogens.

#### 5.1.3. Natural products as inhibitors of NDM-1

**Fig. 15:**
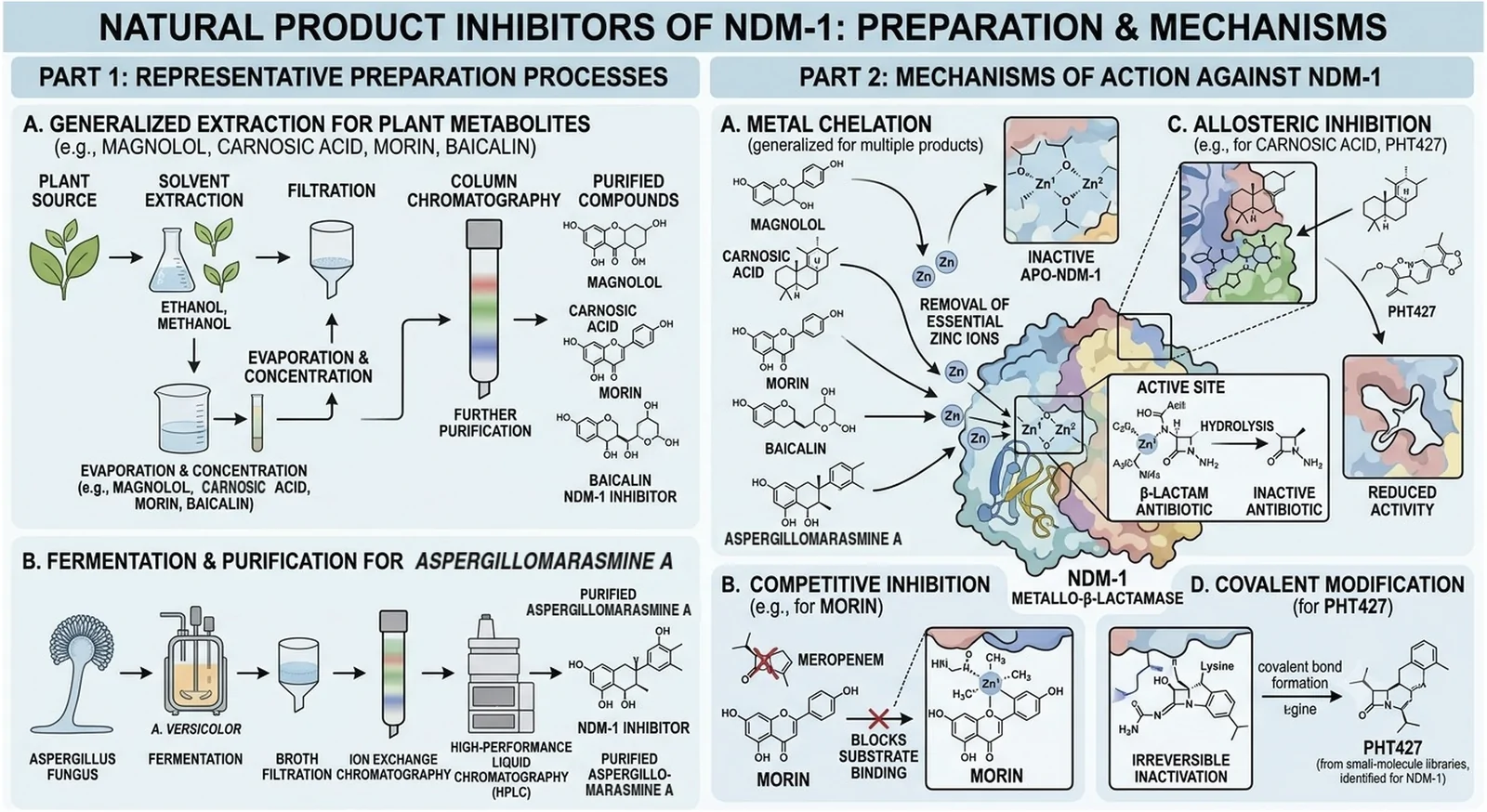
Mechanisms of natural products for the inhibition of NDM-1: Part 1: representative preparation process (A) generalized approach (B) fermentation and downstreaming; Part 2: mechanisms of action against NDM-1 (A) metal chelation (B) competitive inhibition (C) allosteric inhibition (D) covalent modification (image generated using Adobe Firefly Image v3).

Natural products have traditionally been the foundation of antimicrobial drug discovery, providing chemically diverse scaffolds with novel biological activities. Their potential to tackle antibiotic resistance, particularly through the inhibition of resistance enzymes such as New Delhi metallo-β-lactamase-1 (NDM-1), is now better appreciated. In contrast to synthetic compounds, natural products often contain complex, chiral structures that target multiple binding sites, enabling high specificity and lower off-target toxicity. This organizational complexity, often unavailable in standard synthetic chemistry, confers a special advantage for targeting the solvent-accessible, dynamic active site of NDM-1. Natural product inhibitors generally exploit mechanisms such as zinc chelation, active-site blockade, or disruption of structural motifs critical to enzymatic activity (Fig. 15).

##### 5.1.3.1. Magnolol

**Fig. 16:**
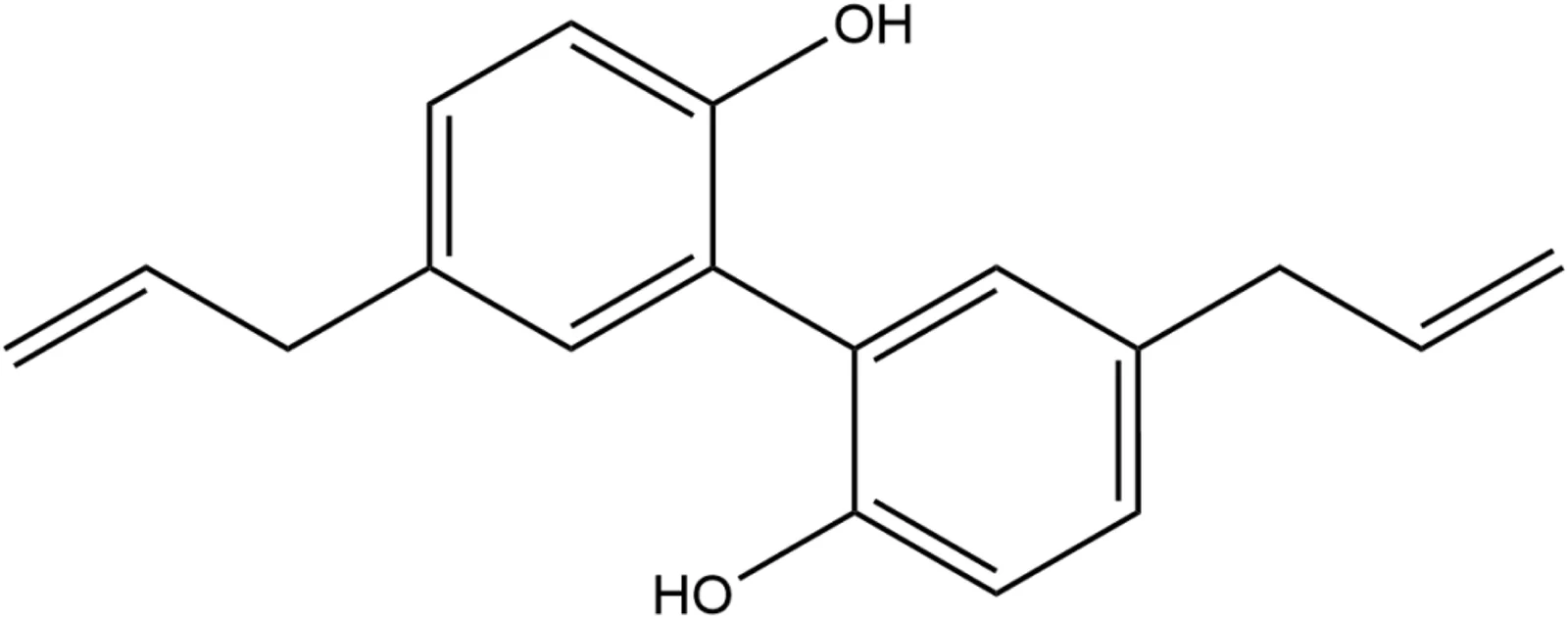
Magnolol having two biphenyl cores along with two allyl substituents and phenolic hydroxyl groups.

Magnolol (5,5′-diallyl-2,2′-dihydroxybiphenyl) is a naturally occurring polyphenolic compound with a binaphthalene framework that is a structural isomer of honokiol, isolated from the bark of *Magnolia officinalis*^92^ (Fig. 16). It has recently been in the spotlight as a natural inhibitor of New Delhi metallo-β-lactamase-1 (NDM-1), a zinc-dependent enzyme responsible for the widespread carbapenem resistance in Gram-negative pathogens.^92^ Magnolol, a compound traditionally used in Chinese medicine for its anti-inflammatory, antioxidant, and antimicrobial properties, emerged as a potent and specific inhibitor of NDM-1, devoid of direct antibacterial activity, highlighting its real potential as a promising therapeutic adjuvant in the fight against multidrug-resistant bacteria.^93^

The identification of magnolol's inhibitory properties against NDM-1 was initially reported by Liu *et al.* (2018) after the screening of seventy-five natural compounds for their inhibitory activities against the enzymatic activity of NDM-1.^93^

In enzyme inhibition assays, magnolol had an IC_50_ value of 6.47 µg mL^−1^ against NDM-1, reflecting its strong inhibitory potency. This was remarkable since only a few natural compounds tested under similar conditions exhibited a comparable level of inhibition.^94^ More importantly, the inhibitory effect was not antagonized by the addition of excess zinc ions, ruling out a simple zinc-chelation mechanism for magnolol's actions, unlike classical metallo-β-lactamase inhibitors such as aspergillomarasmine A.^12,92^ Instead, magnolol acts by directly engaging residues in the catalytic pocket of the enzyme, thus occluding substrate access and abrogating hydrolytic activity; hence, it is an effective inhibitor of the enzyme, a metalloenzyme probably classified in the matrix metalloproteinase (MMP) family or related classes like α-glucosidase or PTP1B.^95^ This distinct inhibitory mechanism not only enhances selectivity but also reduces the likelihood of cross-reactivity with other human metalloenzymes, a common issue with traditional chelators such as EDTA, which indiscriminately sequester metal ions.^93^ Molecular docking and MD simulations provide further insights into the magnolol-binding mode to NDM-1. Liu *et al.* (2018) demonstrated that magnolol bound to the enzyme's active site, with interactions involving residues between positions 110 and 200.^93^ The binding mode analysis showed that van der Waals interactions and hydrophobic interactions were the main forces that stabilized the NDM-1 magnolol complex, with Val73, Lys211, Leu218, Gly219, and His250 playing major roles. The stability of the complex was further enhanced by the formation of a hydrogen bond between magnolol and Ser217. The mutagenesis studies confirmed these predictions that alanine replacements of Lys211 and Gly219 significantly reduced the magnolol binding affinity and inhibitory potency. These findings indicate that magnolol binds to the active site of NDM-1 with high specificity and efficiency, preventing β-lactam substrates from binding to the active site and neutralizing the enzyme's hydrolytic activity against carbapenem antibiotics. This non-metal chelation, direct-binding mechanism is a significant step towards a safer, more specific way to blocking enzyme activity than the conventional metal-depleting strategy.^94^

These findings were also confirmed in *in vitro* antibacterial experiments, which showed that magnolol acts as a β-lactamase inhibitor but not as an antibiotic. Magnolol alone was not active against NDM-1-producing *E. coli* strains (MIC > 1024 µg mL^−1^).

In the presence of meropenem, magnolol restored the antibacterial activity of NDM-producing strains. The MIC of meropenem was reduced 4-fold in *E. coli* ZC-NY3, *E. coli* ZC-YN5, and *E. coli* ZC-YN7, which produce NDM-1, NDM-5, and NDM-9, respectively. The 0.218 FIC index indicated that magnolol and meropenem exerted a synergistic rather than an additive effect. Additionally, time-kill experiments showed that a combination of magnolol and meropenem completely eradicated bacterial populations after 3 hours, unlike meropenem alone. The results highlight the potential of magnolol as a resistance-modifying agent to re-sensitize current carbapenems to NDM-1-producing pathogens.^94^

While research is underway to address its drawbacks, low water solubility, low bioavailability, and a short half-life greatly hinder the clinical application of magnolol. The majority of the studies on the biological activity of magnolol have been carried out *in vitro* and refer to the parent molecule. However, recent research has shown that the parent form of magnolol is unlikely to be found in the systemic circulation due to its rapid metabolism. This has made it unclear whether the beneficial effects demonstrated in the laboratory are directly applicable to the *in vivo* environment. Moreover, there are no clinical trials or human studies to date confirming the effectiveness of magnolol. To confirm its therapeutic value, it will need to undergo full preclinical and clinical investigations. There are also some metabolites and analogues of magnolol with high biological activity. Thus, additional research on the *in vitro* and *in vivo* functions of these metabolites and analogues is needed to provide evidence for the design of new and more potent magnolol-derived drug candidates.^92^

##### 5.1.3.2. Carnosic acid

**Fig. 17:**
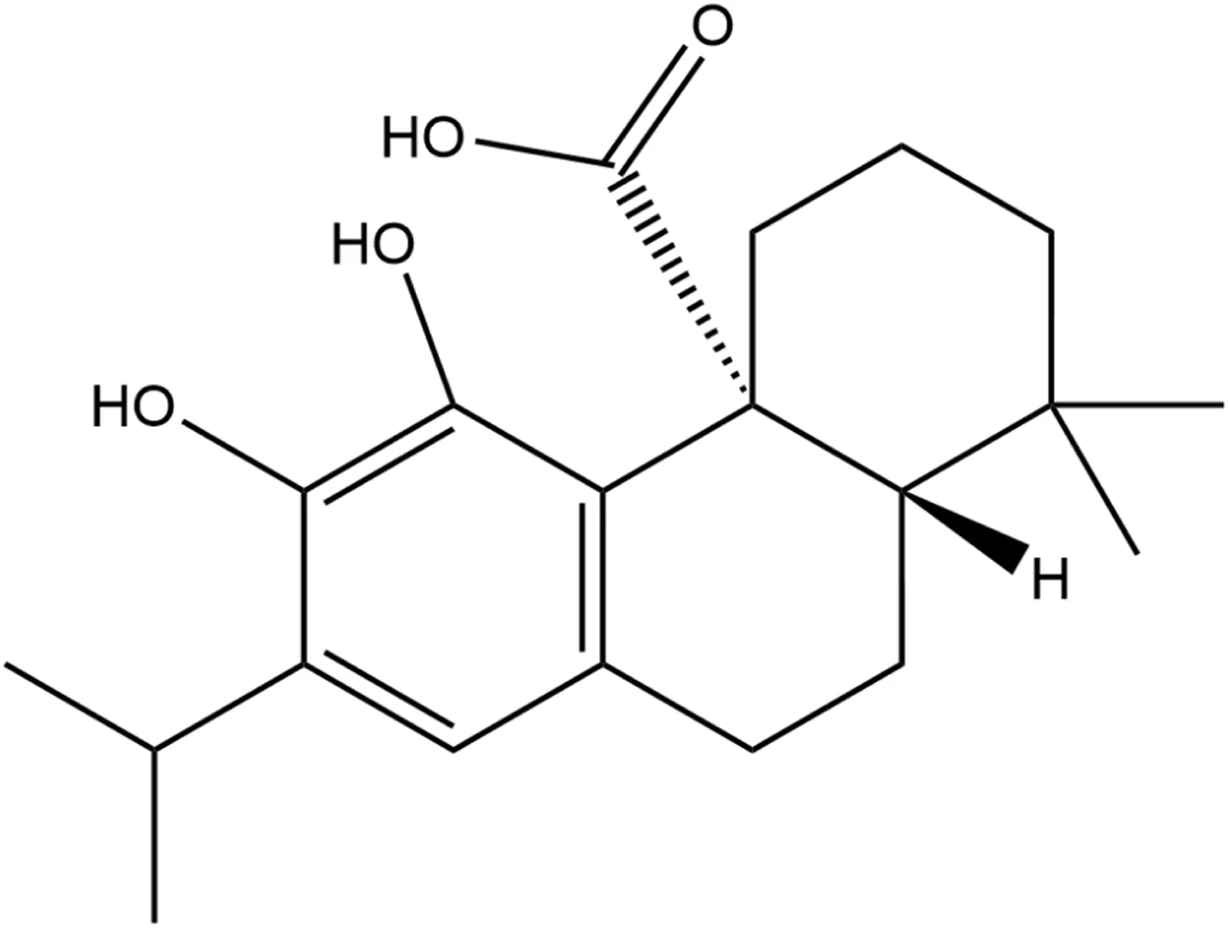
Structure of carnosic acid consisting of a phenanthrene diterpene core with two catechol (dihydroxyl) moieties and a terminal carboxylic acid group.

Carnosic acid is a labdane-type diterpene, naturally occurring in several members of the Lamiaceae family, most notably Rosemary (*Rosmarinus officinalis*) and sage (*Salvia officinalis*).^96^ The lipophilic compound is well known for its antioxidant properties, which have enabled its application in the food and beverage industry, cosmetics, nutritional supplements, and general health products.^96^ Most recently, carnosic acid has emerged as a new weapon against antibiotic-resistant bacteria (Fig. 17).

Yang *et al.* (2020), for example, found that carnosic acid has the strongest inhibition against NDM-1 (IC_50_ value; 27.07 µM) using structure-based virtual screening.^97^ Crucially, carnosic acid lacked direct antibacterial activity, suggesting that it is an enzyme inhibitor. However, when combined with meropenem, it increased the antibiotic susceptibility of *E. coli* ZC-YN3, which overexpresses NDM-1. The combination treatment showed a strong synergistic effect, significantly inhibiting bacterial growth even when treated with meropenem alone. Carnosic acid's action on metallo-β-lactamase is not *via* zinc sequestration. Structural studies revealed that carnosic acid inhibits NDM-1 through an allosteric mechanism. While most β-lactamase inhibitors bind to the active site that holds the catalytic zinc, carnosic acid binds to an allosteric site, which is not the site of catalysis and regulates the active site. Molecular docking and MD simulations showed that the binding site of carnosic acid was not in the active site; binding of carnosic acid changed the shape of the active site, increasing the distance between the active site and substrate-binding amino acids, leading to the loss of catalytic activity.^97^ In energy decomposition studies, the amino acids Phe46, Tyr64, Asp66, Leu65, and Thr94 were found to be important for van der Waals and electrostatic interactions between the ligand and the enzyme. These are the most significant amino acids in this complex, with their Δ*E*_vdw_ values less than −0.8 kcal mol^−1^, suggesting significant contributions of hydrophobic interactions to the binding energy. The authors also created site-directed mutants of NDM-1 (Phe46Ala, Leu65Ala, and Thr94Ala). These enzyme mutants were less susceptible to carnosic acid inhibition, highlighting the importance of interactions with these amino acids for inhibitor binding and subsequent enzyme inactivation. Kinetic measurements showed that the reaction rate for wild-type NDM-1 with carnosic acid was 0.0074, whereas for the mutant enzymes it was much higher (0.0216–0.0228), indicating the loss of inhibition. This experimental validation showed that the inhibitory function of carnosic acid depends heavily on its wild-type structure. The lack of inhibition when residues were substituted confirmed that the mechanism of action of carnosic acid is based on specific interactions rather than nonspecific binding. Unlike other inhibitors, molecular modelling revealed that carnosic acid binds to NDM-1 *via* strong interactions with amino acid residues Phe46, Tyr64, Leu65, Asp66, and Thr94. These amino acids are well removed from the active site and appear to represent allosteric sites. Structural changes at the active site occur following carnosic acid binding to these allosteric sites, causing loss or decrease in enzyme activity.^97^

##### 5.1.3.3. Morin

**Fig. 18:**
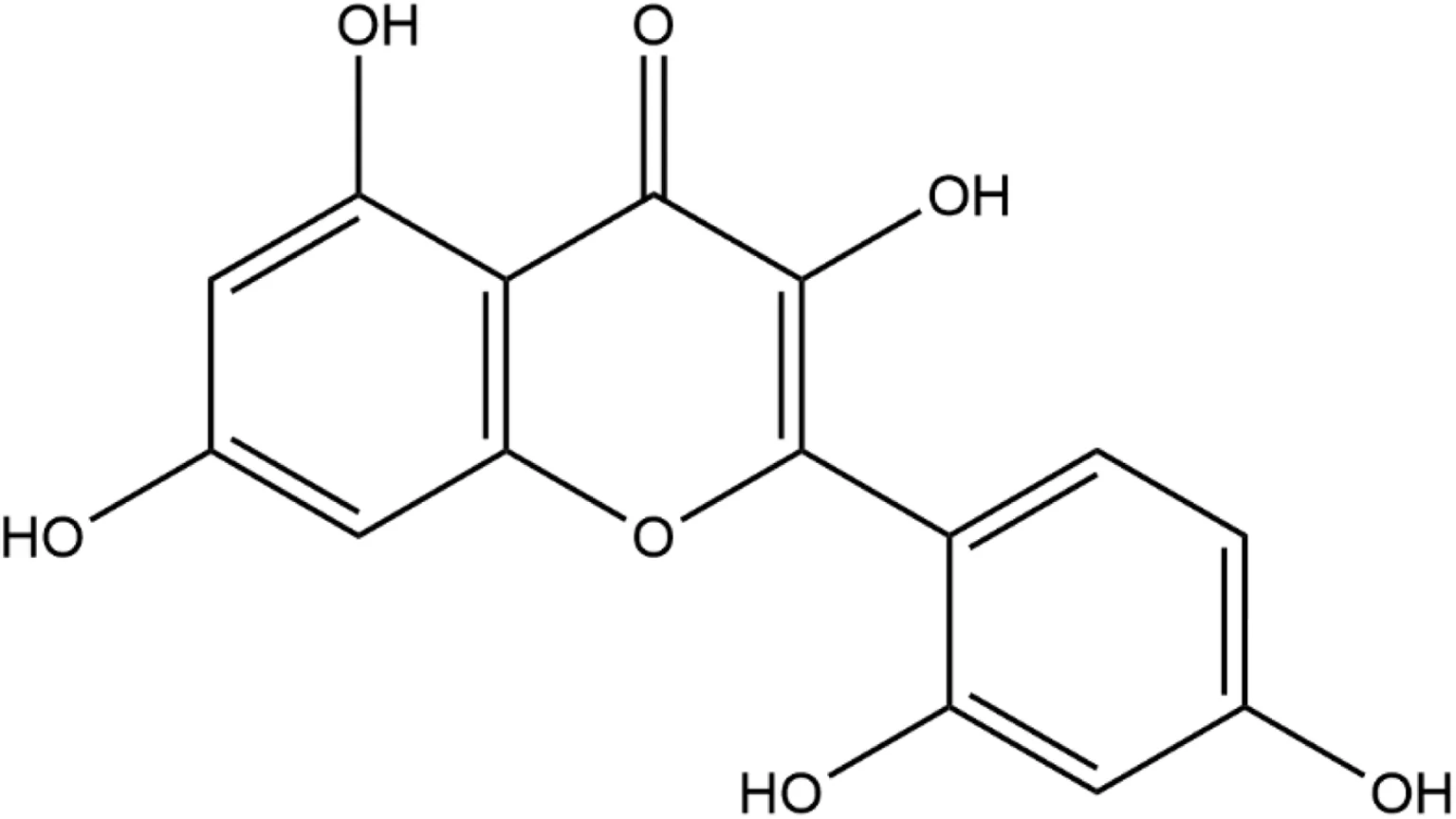
Structure of morin with a flavonol (chromen-4-one) core, polyphenolic hydroxyl groups and a phenyl (B) ring.

Morin (3,5,7,2′,4′-pentahydroxyflavone) is a flavonoid found in many plants, and has recently been shown to be a potent inhibitor of NDM-1 (Fig. 18).^98^ In the structure-based virtual screening of natural product libraries, Ren *et al.* (2025) identified morin as a promising candidate that could bind with the active sites of NDM-1.^98^ Molecular docking studies revealed that morin interacts with critical amino acid residues, including His93, His160, and His191, in the enzyme's zinc-coordinating catalytic region and may thus chelate the essential Zn^2+^ ions. Although morin alone has no significant antibacterial activity against NDM-1-producing *E. coli* (MIC = 512 µg mL^−1^), when combined with β-lactam antibiotics such as meropenem, it strongly potentiates their effectiveness. The checkerboard assay showed that morin synergistically reduced the MICs of these antibiotics (4- to 32-fold) *in vitro*.

Time-kill kinetics also confirmed this synergistic effect, with the culture of NDM-1-positive *E. coli* treated with morin + meropenem showing greater and more rapid bacterial kill than meropenem alone.

Moreover, in NDM-1-expressing *E. coli*-infected BALB/c mice, morin + meropenem caused inflammation and damage, relative to meropenem. In addition, this co-treatment decreased blood cell count, reduced levels of pro-inflammatory cytokines (TNF-α, IL-2, and IL-6) in the serum, and significantly lowered the bacterial load in the liver. Histopathological examination of liver, spleen, and intestinal tissues revealed that the co-treatment led to a more normal tissue architecture, with less necrosis, compared to infected and untreated mice. Additional MD simulations also revealed the stability of the morin-NDM-1 complex with an average free binding energy of −22.82 ± 4.96 kcal mol^−1^.

While these findings are exciting, the current study has limitations. While morin has been shown to have strong synergistic antibacterial and anti-inflammatory effects in mice, the evidence for its effectiveness in humans is lacking. In addition, the capacity of morin to synergise with other β-lactam antibiotics and against other MBLs (beyond NDM-1) has not been fully investigated. Perhaps, the most important limitation of the study is the absence of direct chemical measurements of morin's inhibition, such as an IC_50_ or *K*_i_ value. These values would be crucial for determining the kinetics of the interaction between morin and NDM-1 and for determining the true potency of the inhibitor.^98^

##### 5.1.3.4. Baicalin

**Fig. 19:**
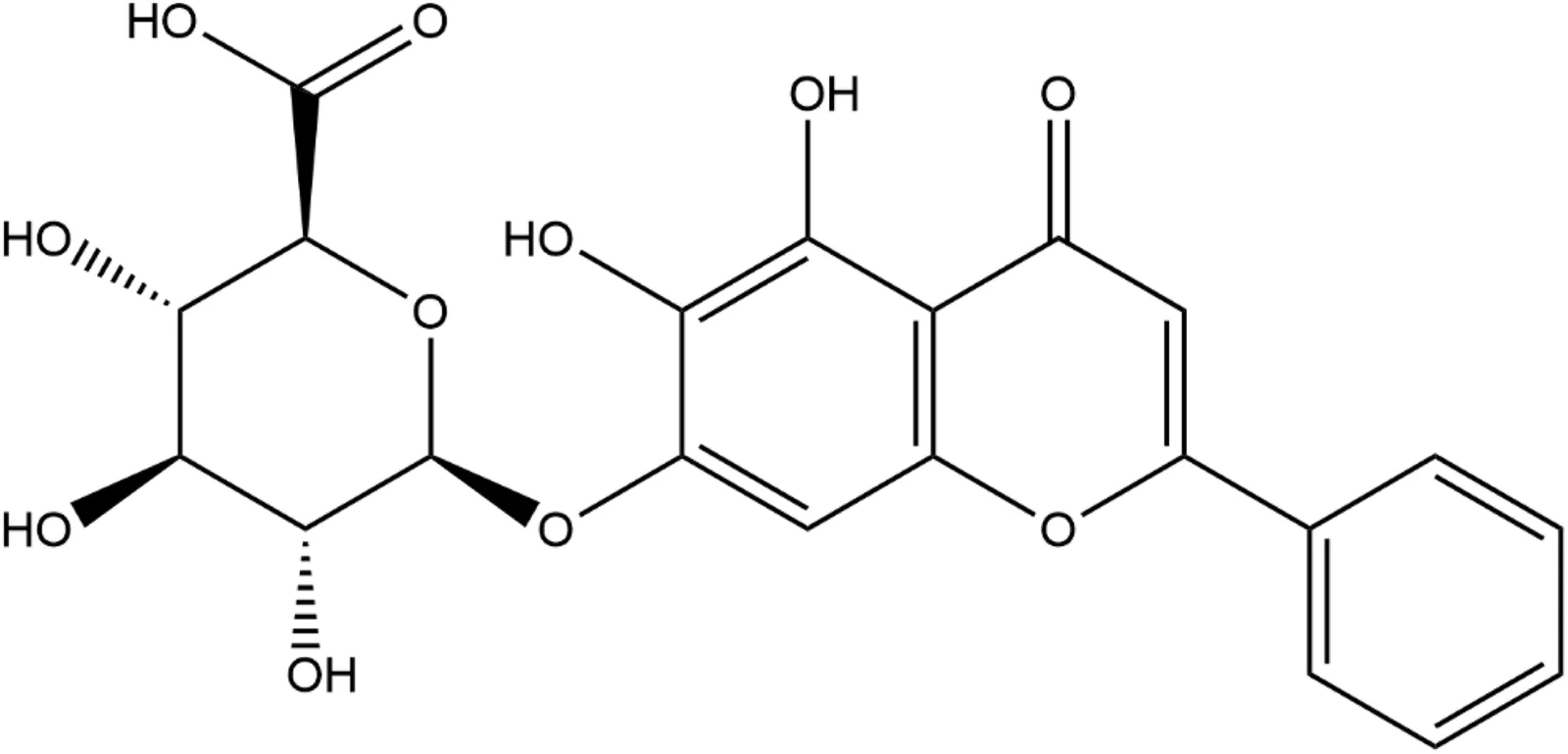
Structure of baicalin having a flavone (chromen-4-one) core with a glucuronic acid moiety and phenolic hydroxyl groups.

Baicalin (7-d-glucuronic acid-5,6-dihydroxyflavone) is the 7-*O*-glucuronide derivative of the flavone baicalein and is one of the principal bioactive constituents of *Scutellaria baicalensis*.^99^ It has recently emerged as a promising natural-product inhibitor of NDM-1 (Fig. 19). Indeed, structure-based virtual screening initially identified this natural product among several top-ranked candidates that bind within or near the NDM-1 catalytic pocket. A recent comprehensive study combining computational predictions with biochemical evaluation by Shi *et al.* (2019) has shown that baicalin inhibits purified NDM-1 with an IC_50_ value of 3.89 ± 1.1 µM, thereby categorizing it among the few natural compounds capable of low-micromolar inhibition of this highly resilient metalloenzyme. Notably, baicalin restored the susceptibility of an NDM-1-expressing *Escherichia coli* strain to β-lactam antibiotics, including clinically important carbapenems, with particular emphasis on its potential as an antibiotic adjuvant rather than a standalone antibacterial agent.^100^

Shi *et al.* (2019) conducted molecular docking and MD simulations to elucidate the molecular basis of this inhibition. The computational analysis indicated that baicalin forms a highly stable complex with NDM-1 *via* both metal-proximal interactions and residue-specific binding. Its carboxylate-containing glucuronide group points toward the binuclear Zn^2+^ catalytic centre, thereby enabling interactions that disrupt the metal-assisted hydrolytic mechanism of NDM-1. Furthermore, baicalin forms strong hydrogen bonds and hydrophobic contacts with important residues at Glu152, Gln123, Met67, Trp93, and Phe70, which together stabilize its position in the catalytic cleft. Per-residue free-energy decomposition analyses further underscored the contribution of these residues to the inhibitor's binding affinity. Taken together, this mechanistic insight suggests that baicalin may partially disturb the geometry of the catalytic site, thereby impairing its ability to coordinate and hydrolyze β-lactam substrates.^100^ Until now, several key factors have hindered the clinical advancement of baicalin, including the incomplete identification of its precise molecular targets and an inadequate understanding of its long-term biological effects. These knowledge gaps point to a cautious re-evaluation of available preclinical data and clarification of the exact mechanisms by which baicalin exerts activity. Furthermore, clinical investigations with rigid study designs are necessary to establish its therapeutic value, particularly with respect to its oral absorption, pharmacokinetic profile in humans, and overall long-term safety profile. Moreover, it should be noted that in most cases, a much higher dose must be administered *in vivo* to achieve *in vitro* phenotype expression due to poor oral bioavailability and extensive biotransformation.^101^

##### 5.1.3.5. PHT427

**Fig. 20:**
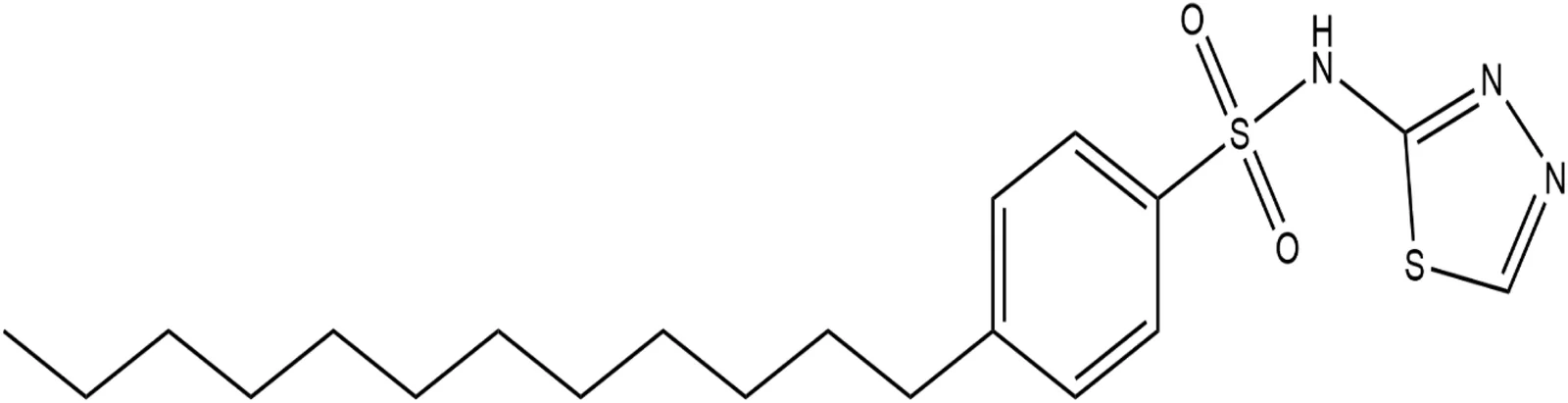
Structure of PHT427 consisting of a benzenesulfonamide core, 1,3,4-thiadiazole ring and dodecyl (C12 alkyl) chain.

PHT427, chemically 4-dodecyl-N-1,3,4-thiadiazol-2-yl-benzenesulfonamide, has been recently identified as a promising inhibitor of New Delhi metallo-β-lactamase-1 (NDM-1) with strong potential to restore carbapenem antibiotic efficacy^102^ (Fig. 20). Li *et al.* (2023) discovered, from high-throughput screening of small-molecule libraries, that PHT427 inhibited the catalytic activity of NDM-1 in a potent and dose-dependent manner, yielding an IC_50_ value of 1.42 µM.^102^ Mechanistic studies uncovered that PHT427 acts through zinc chelation at the NDM-1 active site and *via* interactions with specific, catalytically important amino acid residues. Conformational changes in the enzyme structure were indicated by fluorescence-quenching experiments, which showed that binding of PHT427 concentration-dependently quenched NDM-1's intrinsic tryptophan fluorescence.

Surface plasmon resonance (SPR) assays further confirmed that there was a moderate binding affinity between PHT427 and NDM-1, with a *K*_d_ value of approximately 6.28 × 10^−5^ M. Molecular docking studies provided further insights into the mode of interaction of PHT427 within the NDM-1 active site, the sulphonamide oxygen atom of PHT427 coordinates with both zinc ions (Zn1 and Zn2), while hydrogen bonds form between PHT427 and critical residues such as Asn220, Asp124 and Gln123. In support of these computational results, the authors created mutant versions of NDM-1, namely N220A and Q123A, demonstrating that these mutations strongly diminished PHT427 binding and thus confirmed the functional role of these residues in stabilizing the inhibitor-enzyme complex. Further support for the chelation-based mechanism came from a zinc supplementation assay: the inhibitory effect of PHT427 was partially reversed by excess Zn^2+^, suggesting that its binding and inhibition are at least partially dependent on zinc coordination.^102^ Importantly, PHT427's mode of action is apparently dual: by both chelating the catalytic zinc ions and engaging key active-site residues, it disrupts NDM-1 function more robustly than a simple metal chelator. Functionally, PHT427 demonstrated potent synergy with meropenem *in vitro* in bacterial assays. PHT427, when used in combination with meropenem, restored antibacterial activity, decreasing the MIC by up to 128-fold in NDM-1-expressing *Escherichia coli*. In line with this, there was a significant synergistic activity (FICI 0.5) against clinical isolates of *E. coli* and *K. pneumoniae* carrying NDM-1, including strains carrying KPC-2. Safety profiling suggested that PHT427 has a favourable cytotoxicity profile. The compound showed minimal toxicity in mammalian cell lines (Vero and HepG2 cells), with IC_50_ values well above the levels required for enzyme inhibition. The combination of PHT427 with current antibiotics can treat carbapenem-resistant bacterial infections *in vivo*, but this has not yet been determined. This important question remains to be addressed in future studies.^102^

##### 5.1.3.6. Aspergillomarasmine A

**Fig. 21:**
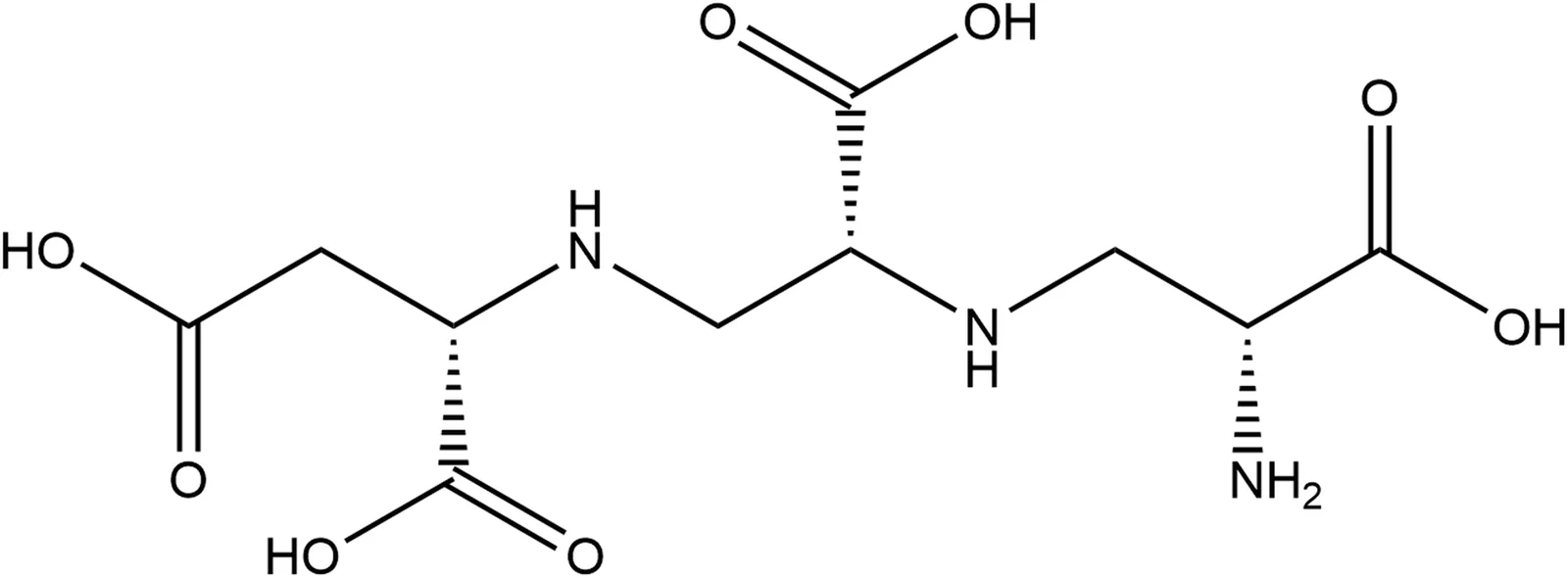
Structure of AMA highlighting its major structural features: amino acid backbone, multiple carboxylic acid groups, and amino functional groups.

Aspergillomarasmine A (AMA), a fungal aminopolycarboxylic natural product from *Aspergillus versicolor*, is one of the earliest and most significant findings of the search for natural inhibitors of NDM-1 (Fig. 21). Through a phenotypic screening, AMA restored carbapenem susceptibility against multiple MBL-producing Gram-negative pathogens, including *E. coli*, *K. pneumoniae*, *P. aeruginosa*, and *Acinetobacter* spp.^103^ At the same time, AMA has no intrinsic antibacterial activity. The inhibition of MBLs was strong in the presence of this compound, which targets the metal dependence rather than the protein framework of metallo-β-lactamases.

*In vitro* IC_50_ inhibition assays of AMA against NDM-1 determined an approximate value of 4.0 µM. AMA reduced meropenem MICs by up to 128-fold against NDM-1-producing clinical isolates, making it one of the most potent natural-product MBL inhibitors reported.^103^ Biochemical and structural analyses of AMA's mechanism of action have shown that AMA acts as a selective Zn^2+^ chelator, displacing or disturbing Zn^2+^ in the sub-nanomolar range, with a dissociation constant *K*_d_ ≈ 0.2 ± 0.04 nM and high selectivity towards zinc compared to other biologically important metals such as Mg^2+^, Ca^2+^, and Mn^2+^.^8^ This property can explain why AMA has a strong impact on MBLs but does not affect host metalloproteins. NDM-1 has a high-affinity (Zn1) and a low-affinity (Zn2) metal-binding site. AMA therefore selectively affects the Zn2 site, by trapping Zn^2+^ that transiently leaves the enzyme. Further mechanistic insights were obtained from maleimide labelling studies, which revealed that Cys208 was more exposed in the AMA-treated state, but is normally protected by Zn^2+^ in the Zn^2+^-bound state. This is consistent with the selective removal of Zn^2+^ and the consequent destabilization of the active site. Crucially, AMA inactivation is due to the low Zn^2+^ affinity of some NDM-1 variants; therefore, AMA is most effective against MBLs with low Zn^2+^ affinity. For example, NDM-6 and IMP-7, which display higher Zn^2+^ affinity and enhanced structural stability, show reduced susceptibility to AMA.^8^ The clinical relevance of AMA was also demonstrated in a mouse peritonitis model, in which the co-administration of AMA with meropenem significantly increased survival in animals infected with NDM-1-producing *E. coli*. Neither AMA nor meropenem alone conferred a survival benefit, but together they fully rescued β-lactam activity.^103^ There are inherent limitations to AMA's mechanism because bacteria can evolve MBLs that function more efficiently under low zinc availability.^104^ All allelic forms carry at least one mutation that increases either the zinc affinity or the structural stability of the enzyme. Consequently, these evolutionary changes diminish the susceptibility of NDM enzymes to zinc chelating inhibitors such as AMA.

#### 5.1.4. Metal chelators

**Fig. 22:**
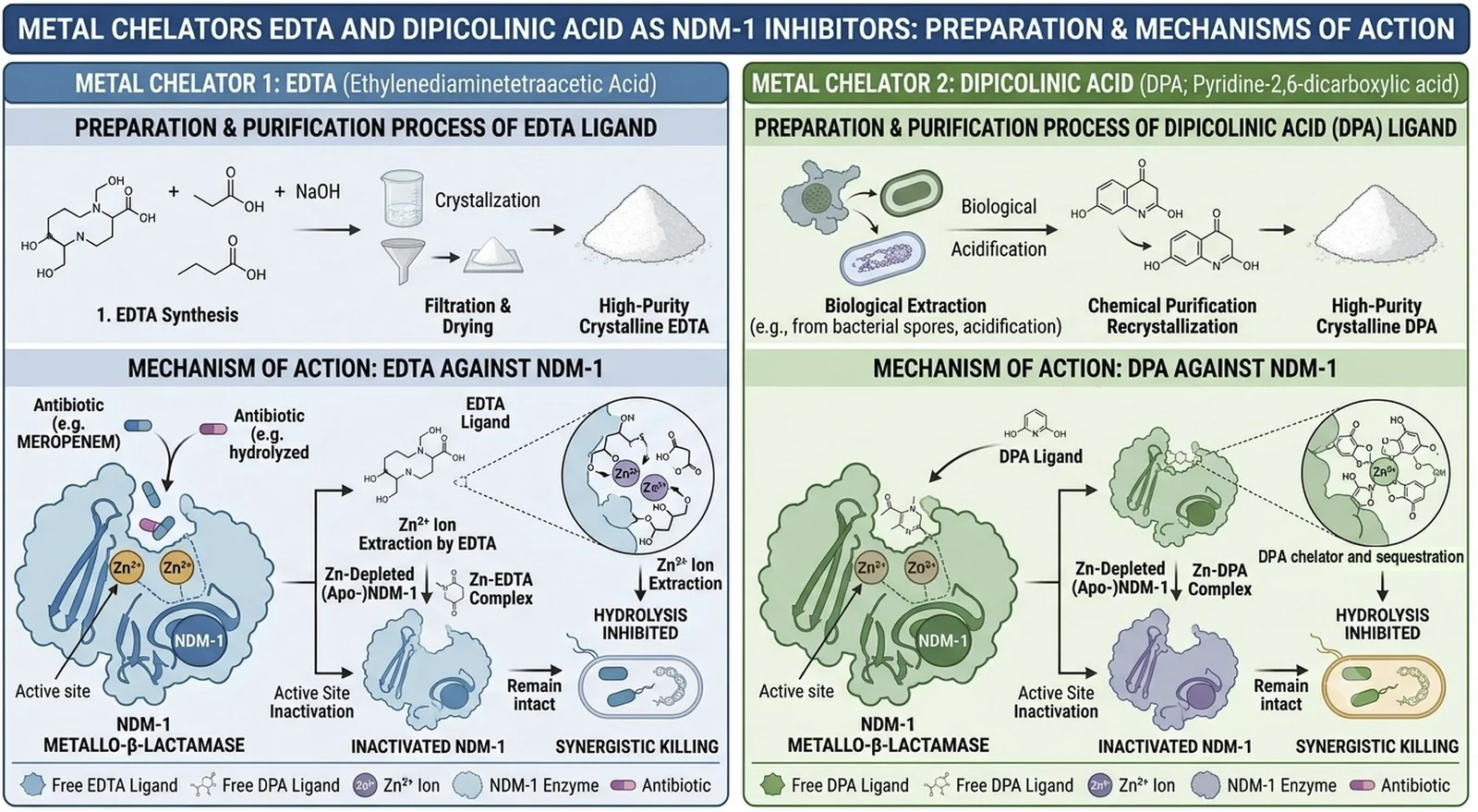
Schematic of metal chelators as the inhibitors of NDM-1: (1) EDTA (2) dipicolinic acid (image generated using Adobe Firefly Image v3).

NDM-1 strictly relies on Zn^2+^ ions in its active sites for its catalytic activity. These Zn^2+^ ions are coordinated to various highly conserved residues, such as histidine, cysteine, and aspartate, which polarize the β-lactam carbonyl and activate a water molecule. This water molecule is then used in nucleophilic attack on the β-lactam ring.^105,106^ Because the zinc ions are crucial for catalytic activity, one of the most direct and efficient ways to inhibit NDM-1 is through the deprivation of these essential metal cofactors by chelation (Fig. 22).

##### 5.1.4.1. Ethylenediaminetetraacetic acid

**Fig. 23:**
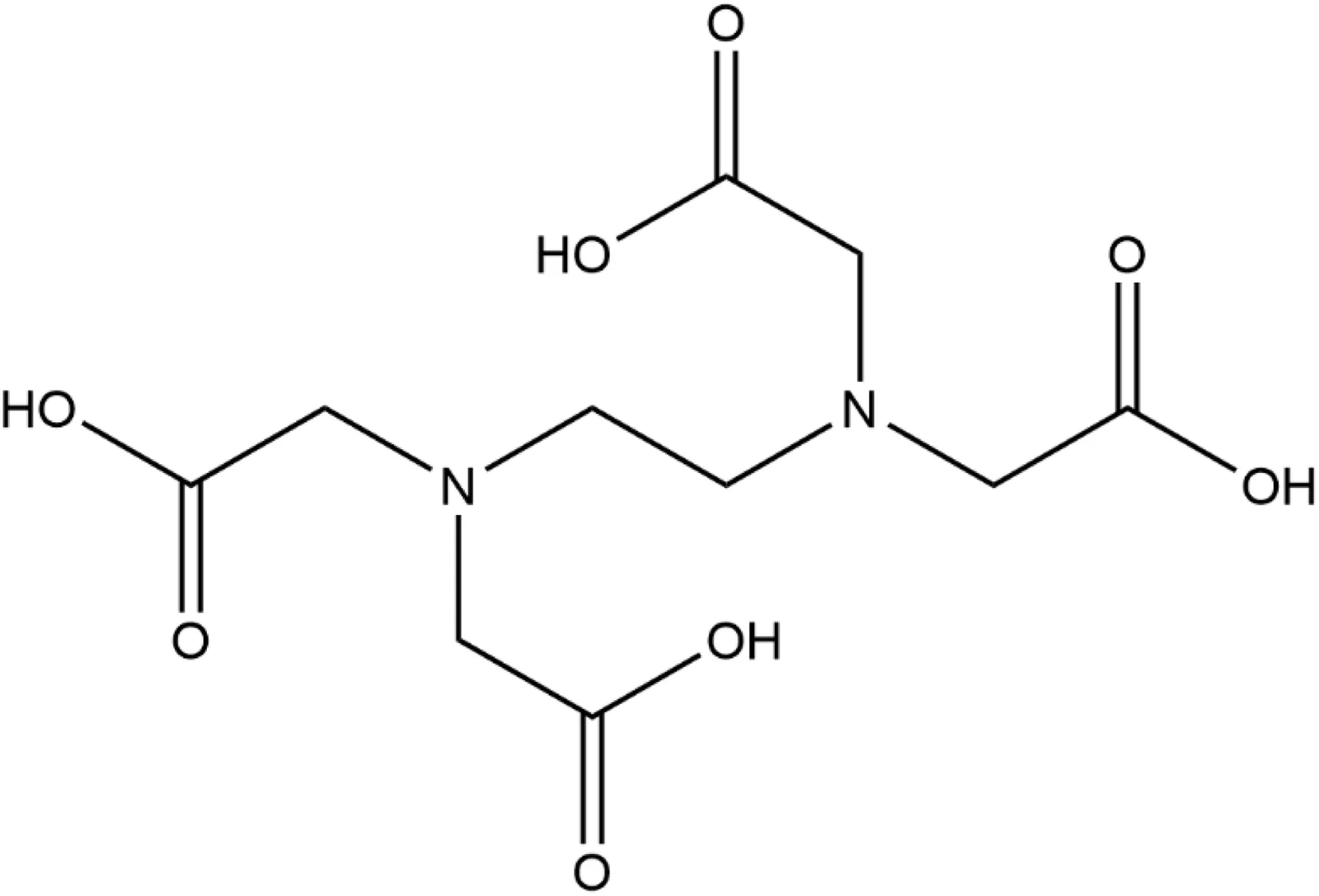
Structure of ethylenediaminetetraacetic acid.

Ethylenediaminetetraacetic acid (EDTA) is a well-characterized metal chelator widely used in biochemical and microbiological studies to inhibit MBLs (Fig. 23). EDTA has been shown to tightly bind Zn^2+^ ions in the active site of MBLs, such as NDM-1, thereby effectively stripping the enzyme's essential metal cofactor and inactivating its catalytic function.^107,108^*In vitro* synergy assays have demonstrated that combining EDTA with carbapenems, such as imipenem or meropenem, restores antibiotic activity against NDM-1-producing *E. coli*. For instance, checkerboard dilution studies yielded fractional inhibitory concentration (FIC) indices <0.5, demonstrating strong synergistic effects.^108^

In another kinetic study using purified NDM-1 enzyme, an inhibitory effect potentiated by EDTA was observed when it was combined with other inhibitors. When EDTA was mixed in a ratio of 1 : 2 with aspergillomarasmine A (AMA), the activity of NDM-1 was suppressed by more than 95% within minutes, which largely exceeded the activity of either compound alone.^107^

These results confirm that EDTA can restore the carbapenem efficacy against NDM-1-expressing strains, though its clinical utility is limited by its lack of specificity, possible toxicity, and high concentrations required for inhibition.^108^

##### 5.1.4.2. Dipicolinic acid (DPA) and derivatives

**Fig. 24:**
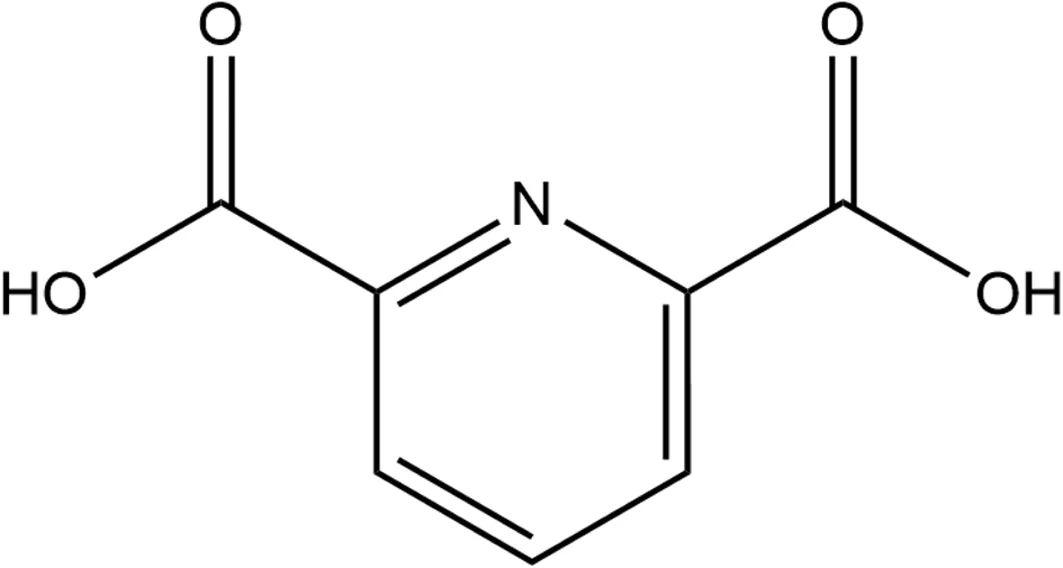
Structure of dipicolinic acid.

Dipicolinic acid (DPA), or pyridine-2,6-dicarboxylic acid, has emerged as a foundational scaffold for the development of targeted inhibitors of the NDM-1 enzyme^109^ (Fig. 24). Fragment-based screening of metal-binding pharmacophores identified DPA as a primary compound that inhibited NDM-1 at 520 nM, primarily by coordinating the enzyme's dinuclear Zn(ii) active site. Unlike broad-spectrum chelators like EDTA, the binding of DPA is more controlled; its spectroscopic studies have shown that DPA can certainly withdraw Zn(ii) from the active site but is much less aggressive than EDTA, and its inhibition involves both metal chelation and coordination at the catalytic center.^110^

Structural studies further suggest that DPA interacts with active-site residues, especially Gln123, Asn220, and Lys211, as well as the hydrophobic β-hairpin loop, thereby allowing the compound to mimic the carboxylate interactions of a hydrolyzed β-lactam product and to stabilize a metal-bound inhibitory complex.^110^ Given DPA's moderate potency, extensive structure–activity relationship studies have identified a series of optimized derivatives with significantly improved inhibitory activity. Derivatization at the 4-position of the pyridine ring generated a number of high-affinity inhibitors, most notably “compound 36” [4-(3-aminophenyl)pyridine-2,6-discarboxylic acid], with an IC_50_ value of 80 nM, a four-fold improvement over DPA. Unlike DPA, “compound 36” does not strip Zn(ii) from NDM-1; instead, it forms a stable ternary NDM-1:Zn(ii) inhibition complex, as established by NMR, UV-vis, EPR, and equilibrium dialysis experiments, thereby preventing the metal center while restricting the catalytic turnover. DPA-based inhibitors have demonstrated favorable microbiological activity. Microdilution assays of clinical isolates of *E. coli* and *K. pneumoniae* carrying NDM-1 revealed that the co-administration of compound 36 of imipenem decreased imipenem MICs of the compound to between 0.5 and 1 mg L^−1^, which was sufficient to restore susceptibility, but not to alter bacterial growth by itself. Importantly, compound 36 did not show cytotoxicity against HEK293 mammalian cells at relevant concentrations, supporting its favourable preliminary safety profile.^110^

The long-term prospects for the design of effective NDM-1 inhibitors based on pyridine-2,6-dicarboxylic acid remain unclear. Certain derivatives have been found experimentally to form zinc complexes readily but display reduced NDM-1 inhibitory activity. Such behaviour points to potentially higher off-target effects resulting from nonspecific metal chelation. Considering the above-mentioned limitations, evidence indicates that derivatives of DPA will not be productive, clinically viable scaffolds for the next generation of metallo-β-lactamase inhibitors.^109^

#### 5.1.5. Nanomaterial-based strategies for inhibiting NDM-1

**Fig. 25:**
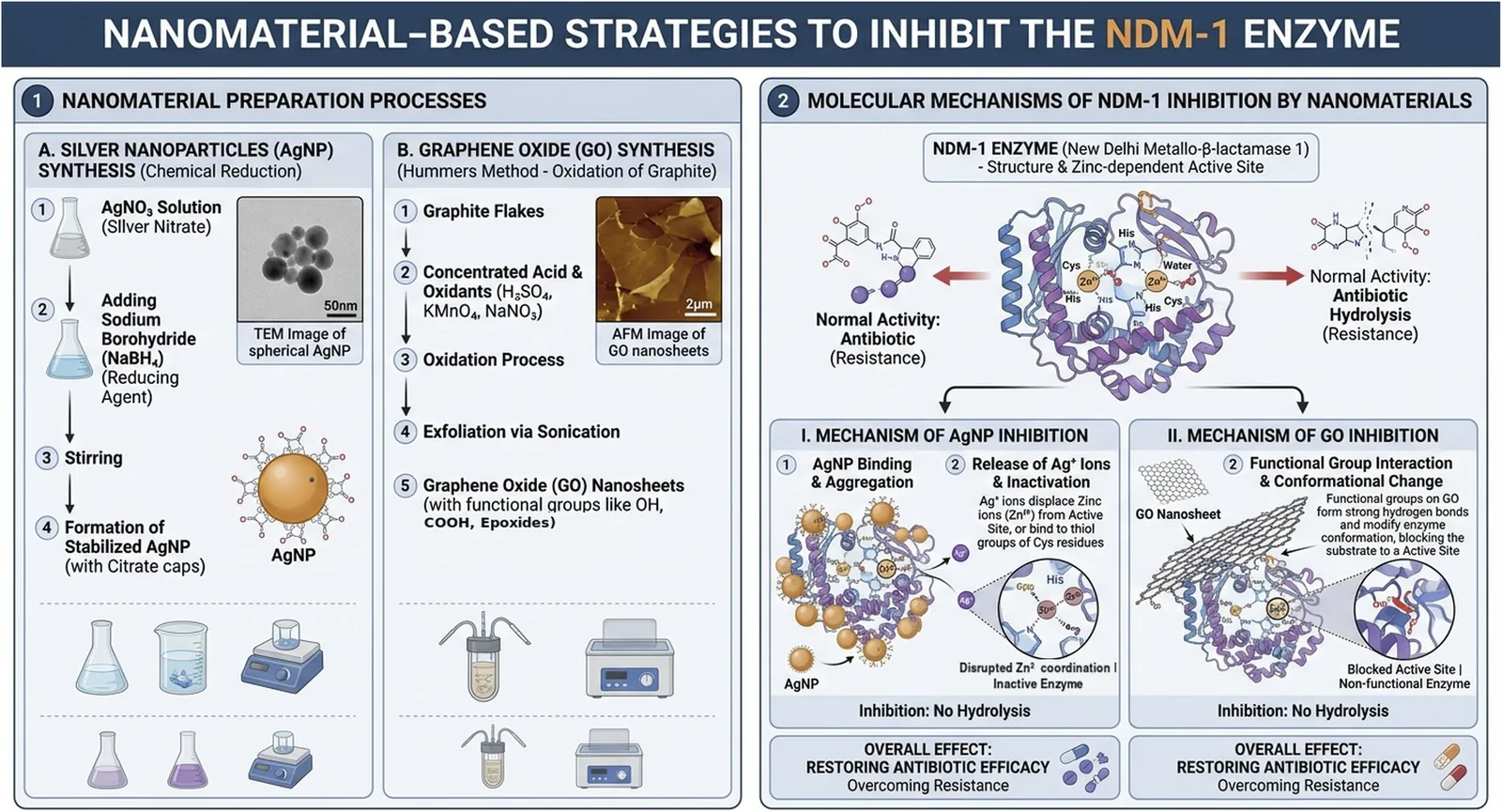
Schematic of the nanomaterial-based NDM-1 inhibitors: (1) nanomaterial preparation processes (A) silver nanoparticles synthesis (B) graphene oxide (2) molecular mechanisms of NDM-1 inhibition by nanomaterials (I) mechanisms of AgNP inhibition (II) mechanism of GO inhibition (image generated using Adobe Firefly Image v3).

The development of nanomaterials as antimicrobial platforms has provided new possibilities for fighting antibiotic resistance, particularly against pathogens producing New Delhi metallo-β-lactamase-1 (NDM-1) enzyme. Nanomaterials, offering tunable physicochemical properties, high surface area-to-volume ratio, and versatile surface functionalization, can enhance the delivery, stability and efficacy of the antimicrobial agents as well as allow targeted interactions with bacterial cells and resistance enzymes (Fig. 25). Metal and metal oxide nanoparticles, polymeric nanocarriers, lipid-based nanoparticles and hybrid nanocomposites have shown the ability to disrupt bacterial membrane, generate reactive oxygen species, interfere with biofilm development and increase intracellular drug accumulation. In the context of NDM-1-mediated resistance, nanomaterials offer an additional advantage as they can act as carriers for metallo-β-lactamase inhibitors or directly interact with the active site of the enzyme, thus restoring the activity of β-lactam antibiotics. The functionalization of nanomaterials with phytochemicals, antimicrobial peptides or small-molecule inhibitors has also been shown to have synergistic effects, which led to increased antibacterial potency with reduced toxicity and probability of resistance development. Thus, nanotechnology-based therapeutic strategies present a promising and novel approach to tackle NDM-1-associated antimicrobial resistance and to extend the clinical use of the existing antibiotics.

##### 5.1.5.1. Silver nanoparticles (AgNPs)

Among the most actively investigated nanomaterial platforms for combating MDR Gram-negative bacteria, including NDM-1-harboring strains, AgNPs exhibit broad-spectrum antimicrobial activity through multiple parallel mechanisms. These may include the controlled release of Ag^+^ ions, generation of ROS, disruption of membrane integrity, and interaction with intracellular targets. AgNPs exert potent bactericidal activity by binding to thiol-containing enzymes, leading to cellular protein or DNA damage and destabilization of the bacterial cytoplasmic membrane. Such a multifaceted attack makes it highly difficult for bacteria to acquire resistance. Therefore, AgNPs represent highly attractive candidates for adjuvant therapy in the era of carbapenem-resistant *Enterobacterales*.^111^

The pharmacodynamic studies show that AgNPs strongly potentiate β-lactam antibiotics such as meropenem and imipenem, with checkerboard assays yielding FIC indices of 0.5, indicating strong synergy, and reductions in MICs of 4- to 32-fold across *E. coli*, *K. pneumoniae*, and *P. aeruginosa* clinical isolates. Time-kill curves show rapid bactericidal effects within 2–4 hours when AgNPs are combined with carbapenem, significantly outperforming monotherapy.^112^ AgNPs exhibited potent intrinsic antibacterial activity against clinically relevant Gram-negative pathogens, including *P. aeruginosa*, with MIC values of 1 µg mL^−1^. Cellular assays indicated that AgNP exposure resulted in severe morphological damage, membrane collapse, cytoplasmic condensation, and disrupted cell division. Biochemical analyses showed that AgNPs induce oxidative stress and protein denaturation, which contribute significantly to rapid bacterial death. Moreover, the combination of AgNPs with β-lactam antibiotics at sub-MIC significantly enhanced their activity, reinforcing their role as efficient adjuvants in antimicrobial therapy and confirming that even low doses can produce clinically meaningful potentiation of antibiotic activity.^113^

AgNPs can be modified not only for enhanced antimicrobial activity, but also for greater biological compatibility and delivery. Stabilizing ligands, such as polysaccharides, proteins, or polymers, functionalize AgNPs to improve pharmacokinetics and lower mammalian cell toxicity, a key issue to address for clinical applications. Haji *et al.* (2022) and Muddasir *et al.* (2022) noted that the biological effects of AgNPs depend on their physicochemical properties, particularly surface chemistry and dose. Unmodified or high concentrations of AgNPs can induce oxidative stress and mitochondrial damage in mammalian cells, but surface-functionalized AgNPs retain anti-microbial activity and offer biocompatibility. Further, the penetration of AgNPs into and disruption of bacterial biofilms and synergism with antibiotics highlight their value for treating NDM-1-producing bacteria that are often predisposed.^112,113^ The authors show that cytotoxicity, oxidative stress, and environmental accumulation are important considerations that could pose limitations to the use of unmodified AgNPs. The toxicity of AgNPs is particle size-, concentration-, and surface-chemistry-dependent and requires control of nano-formulation. In addition, concerns about the persistence of nanoparticles in tissues and the environment call for rigorous long-term safety studies. Innovations such as surface-functionalized AgNPs, low-dose synergistic regimens, and controlled release formulations may mitigate such risks and accelerate translation towards use in clinical purposes.^111,112^

##### 5.1.5.2. Graphene oxide (GO) as an inhibitor

Graphene oxide (GO) has recently emerged as a powerful, broad-spectrum inhibitor of β-lactamases, displaying activity against both SBLs and MBLs. According to Piccirilli *et al.* (2025), GO is made up of highly oxidised, water-dispersible, two-dimensional sheets enriched with hydroxyl, epoxy, and carboxyl functional groups that allow strong interactions with proteins *via* π–π interactions, hydrogen bonding, electrostatic forces, and covalent coupling.^114^ Kinetic inhibition assays revealed that GO displays formidable inhibition towards all SBLs tested, including SHV-1, CTX-M-15, GES-1, KPC-3, and OXA-23, even at the lowest used concentration of 0.5 mg mL^−1^, with residual activity after 60 min of pre-incubation below 10% of the control. GO also inhibited the monomeric MBLs NDM-1, VIM-1, and CphA, producing ∼70–80% loss of enzymatic activity within the first 10 min, while the tetrameric MBL L1 remained markedly less susceptible. Combinative mechanistic studies based on enzyme kinetics, X-ray photoelectron spectroscopy (XPS) and density functional theory (DFT) indicate that GO suppresses NDM-1 *via* a non-metal-chelating pathway. The fact that the changes in Zn 2p_3/2_ binding energies do not occur suggests that the coordination of Zn^2+^ is not significantly affected. Alternatively, GO selectively binds positively charged surface residues, like lysine and arginine. Additional XPS C 1s and N 1s indicate that covalent or quasi-covalent interactions were formed between lysine amine groups and oxidized GO functionalities, which may have occurred through the opening of epoxide rings, to contribute to enzyme inactivation. This interaction immobilizes the enzyme on the GO basal plane, perturbs its tertiary structure, and leads to non-competitive inhibition of catalysis. Further DFT and XPS analyses indicated the possibility of chemisorption of the β-lactam substrate nitrocefin by GO *via* the oxidation of the sulphur atom, thus explaining the reduced inhibitory activity observed upon pre-incubation of GO with the substrate. Therefore, taken together, these observations establish that GO is a universal inhibitor of monomeric β-lactamases, acting *via* structural distortion rather than direct active-site blockage. Moreover, it demonstrates the potential of GO as a nanomaterial-based anti-resistance strategy.^114^ Although graphene oxide has shown numerous promising applications across various fields, concerns about its toxicity have hindered further development. To date, studies have suggested that the toxic effects of GO may depend on factors such as particle size, synthesis method, delivery route, and exposure duration. Most existing studies focus primarily on short-term toxicity, with very limited data on long-term health impacts. In addition, the method of GO administration, its dosage, and its physical and chemical properties are key factors in determining the overall toxicity.^115^

## 6. Emerging approaches: CRISPR-Cas, nanoparticles, and AI-driven drug discovery

**Fig. 26:**
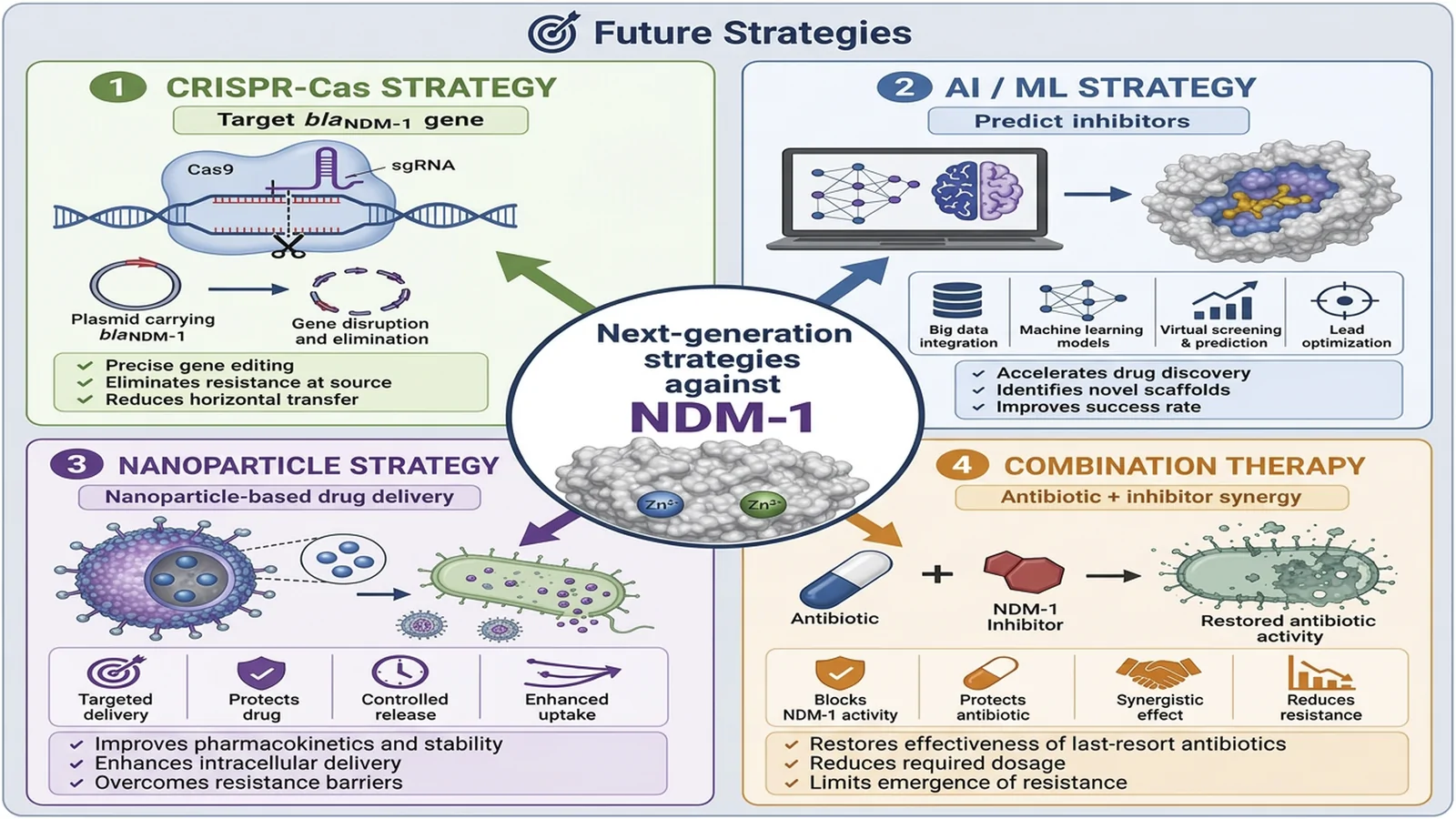
Overview of the new generation of therapeutic approaches as the countermeasures of NDM-1: (1) CRISPR-Cas strategy (2) AI/ML strategy (3) nanoparticle strategy (4) combination therapy (image generated using Adobe Firefly Image v3).

CRISPR-Cas systems allow for precise, programmable genome editing for target validation and potential curative interventions, supporting allele-specific editing, base and prime editing, and epigenetic modulation. In addition, engineered nanoparticles allow for tunable delivery and controlled release with multi-functionalization (targeting ligands, stimuli-responsive triggers, co-delivery of nucleic acids or small molecules) to overcome biological barriers and improve pharmacokinetics. Further the AI-driven drug discovery accelerates target identification, *de novo* design of molecules, virtual screening, and optimization of formulation and delivery by integrating multi-omics, structural and phenotypic data with deep learning and generative models. Together these approaches reduce discovery times and improve therapeutic precision but bring challenges in safety, delivery, interpretability and translation to the clinic (Fig. 26).

### 6.1. CRISPR-Cas

The CRISPR-Cas system has rapidly evolved from molecular biology tools into a new class of precision antimicrobials capable of selectively recognizing and cleaving defined DNA sequences, an attribute that has been exploited to date to target plasmid-encoded antibiotic resistance determinants, including carbapenemases. Proof-of-concept studies have demonstrated that CRISPR-Cas modules can be engineered to excise or disrupt resistance genes on mobile plasmids, thereby resensitizing bacteria to antibiotics. Hao *et al.* (2020) developed a plasmid-borne CRISPR system (pCasCure) that, when introduced into diverse clinical carbapenem-resistant *Enterobacteriaceae*, achieved >94% curing efficiency of plasmids carrying blaKPC, blaNDM, and blaOXA-48 and produced more than an 8-fold reduction in carbapenem MICs in treated isolates, demonstrating that sequence-specific cleavage of plasmid targets can restore antibiotic susceptibility.^116^

Early and related efforts demonstrated that delivery of CRISPR components *via* bacteriophage can selectively deplete resistance plasmids from bacterial populations. Citorik *et al.* (2014) packaged CRISPR arrays into phagemids or lysogenic phages and achieved potent depletions of targeted resistance genes in mixed culture, producing large drops in viable counts of resistant cells and blocking the horizontal transfer of plasmids.^117^ Conjugative delivery systems provide an alternative practical route for the *in situ* dissemination of CRISPR antimicrobials, *e.g.* pheromone-responsive conjugative plasmids encoding CRISPR-Cas modules successfully transferred between *Enterococcus* strains *in vitro* and reduced the abundance of specific resistance determinants in the murine intestine, illustrating that self-propagating CRISPR constructs can be used to edit microbial communities.^118^

This approach is by no means restricted to carbapenemases, as other studies, based on conjugative or host-independent vectors, have successfully ablated mcr-1 and other clinically relevant resistance genes, thereby restoring colistin or other antibiotic susceptibility in recipient strains.^119^ These systems mechanistically exert their effects either by cleaving chromosomal resistance loci (lethality due to unrepaired double-strand breaks) or by cleaving plasmid sequences (plasmid loss and consequent loss of resistance), and can be multiplexed to target several genes or essential plasmid maintenance functions simultaneously. However, significant translational challenges persist, including the difficulty of efficient, species-wide delivery into clinical Gram-negative pathogens, and the spread *via* lysogeny or conjugation raises biosafety and ecological concerns about the horizontal dissemination of CRISPR constructs. Some plasmids evade cleavage *via* recombination or through high copy number, and *in vivo* efficacy, immunogenicity, and potential off-target effects demand detailed consideration. Although CRISPR antimicrobials have indeed achieved powerful sequence-specific plasmid curing and desensitization in the laboratory and animal models, much effort will be required to develop safe, targeted delivery systems and containment strategies before clinical use can be considered.^119^

### 6.2. Metal–organic frameworks (MOFs)

Metal–Organic Frameworks (MOFs) are crystalline porous materials in which metal ions or clusters are coordinated to organic linkers, yielding highly ordered three-dimensional structures with tunable porosity, high surface area, and versatile chemical functionality. These features have brought their unique physicochemical properties to the fore in biomedical applications, especially antimicrobial therapy and drug delivery. Among the various members of the MOF family, Ag-MOFs prevent the growth of *K. pneumoniae*, with a MIC value of 10 µg mL^−1^, with much higher efficiency than silver nanoparticles. The porous scaffold in MOFs stabilizes silver ions, allowing for sustained antibacterial activity with low rates of resistance development.^120^

Cyclodextrin MOFs have been used as biocompatible drug carriers for antibiotics. Moreover, CD-MOFs were non-toxic to mammalian cells both *in vitro* and *in vivo*.^121^ On the other hand, ZIF-8 has been employed as a carrier of ceftazidime, which was released in a sustained manner up to 7 days and was able to enter cells, particularly for pulmonary and intracellular infections.^122^ Notably, ZIF-8 particles were internalized by the macrophages and lung epithelial cells but with no lethal cytotoxicity, indicating great promise for systemic therapy against microbes.^122^ Other zinc-based MOFs, such as MOF-5, IRMOF-3, and Zn-BTC, have also been explored for antimicrobial applications. These frameworks, when used alone or in combination with antibiotics such as ampicillin and kanamycin, exerted a synergistic or additive antibacterial effect against Gram-positive and Gram-negative bacteria, significantly reducing MIC values compared with the drugs alone.^123^ The incorporation of MOFs into biodegradable polymers, such as polycaprolactone (PCL) and poly(lactic-*co*-glycolic) acid (PLGA), further enhances stability and enables sustained drug release. Cephalexin and metronidazole-loaded MOF-5 composites embedded with PCL or PLGA matrices have been reported to show controlled release and exhibit strong antibacterial activity against *S. aureus* and *Staphylococcus epidermis*, *E. coli*, and *Acinetobacter baumannii*, thus underlining the utility of polymer-MOF composites in the fight against antimicrobial resistance.^124^

### 6.3. Artificial intelligence and machine learning in the discovery of NDM-1 inhibitors

The contribution of artificial intelligence (AI) and machine learning (ML) is not just limited to the rapid identification of potential NDM-1 inhibitors, but it can also help identify specific chemical features of inhibitors. Several molecular descriptors have been identified in the analysis of compounds prioritized by AI, which are important for inhibiting NDM-1. These include the presence of zinc-binding functional groups, including thiols, hydroxamates, carboxylates, boronates, and sulfur-containing heterocycles, which enable interaction with the binuclear Zn(ii) catalytic center. Similarly, descriptors like molecular weight, topological polar surface area (tPSA), lipophilicity (log *P*), hydrogen bond donor and acceptor counts, and molecular flexibility are often used to discriminate between active inhibitors and inactive compounds in predictive models.^125^ Several compounds with a balance of hydrophobic and polar properties have been shown to bind well in the NDM-1 active site, with good physicochemical properties. AI-powdered screening campaigns have repeatedly enriched scaffolds with bicyclic boronates, hydroxamate derivatives, thiol-containing compounds, polyphenolic frameworks, and heterocyclic sulfur-containing compounds, from a chemotype standpoint.^126^ They are also quite appealing for their ability to participate in the reactions with catalytic residues, such as His120, His122, Asp124, His189, Cys208, and His250, as well as for forming interactions with adjacent hydrophobic pockets or zinc ions. Thus, AI methods are now playing an increasingly important role in predicting structure–activity relationships (SARs) to rationalize the design of the next generation of NDM-1 inhibitors, in addition to their use as screening tools.^127^ Most importantly, computational predictions generated by these AI/ML frameworks must be experimentally validated before they can be advanced as drug candidates. In recent NDM-1 inhibitor discovery studies, the molecules prioritized by AI have generally undergone a multi-step validation process that includes biochemical enzyme inhibition assays, minimum inhibitory concentration (MIC) reduction assays, checkerboard synergy testing with carbapenems, molecular docking and molecular dynamics studies, and cytotoxicity assays.^72^ Several compounds identified computationally have been shown to have low micromolar activity against purified NDM-1 and to render NDM-1-containing *Enterobacterales* susceptible to meropenem. For instance, AI-powdered virtual screening methods were able to discover compounds like adapalene and carnosic acid that were subsequently confirmed by enzymatic tests and combination susceptibility testing. Furthermore, machine learning-enabled prioritization has helped identify compounds with desirable binding features to the catalytic zinc center and active-site residues that cause measurable decreases in the carbapenem-resistant phenotype. The results underscore the need to supplement computational predictions with biochemical and microbiological validation, not only for theoretical binding affinity but also for the anti-NDM-1 activity of AI-generated hits. In recent years, artificial intelligence approaches to virtual screening and structure-based drug design have emerged as a practical force multiplier against challenging targets. Convolutional neural networks (CNNs) and three-dimensional deep learning architectures have been used to identify binding pockets and to score ligand protein interactions from structural data. Jiménez *et al.* (2017) and colleagues developed Deepsite, a 3D-CNN method trained on thousands of annotated protein binding sites that learned volumetric spatial patterns predictive of ligand binding pockets and outperformed several classical pocket-detection algorithms on benchmark sets. This class of methods is routinely used as a first pass to prioritize likely binding regions on proteins before docking or further modelling.^128^

Graph neural networks (GNNs) and other related graph-based deep models extend these capabilities by representing molecules and even protein sites as graphs, allowing end-to-end prediction of protein-ligand interactions and binding affinity directly from structural or graph representations. The latest architectures of GNNs capture local chemical environments, atom-level connectivity, and higher-order interaction patterns and have been shown in benchmark studies to improve ranking affinity compared to many classical scoring functions, particularly useful when the active site contains metal ions or requires explicit modelling of coordination geometry.^129,130^ These machine-learning scoring functions are increasingly embedded in massive *in silico* screens. A prominent example is deep docking, which applies deep QSAR models trained on subsets of docking results to predict the remaining outcomes in very large libraries, iteratively removing low-probability compounds and thereby enabling the practical screening of billions of entries. For example, those from zinc and enamine-type collections, with orders-of-magnitude lower computational cost than brute-force docking. Deep docking thus converts docking from a bottleneck into a tractable, high-throughput enrichment step that feeds follow-up physics-based refinement and experimental testing.^131^

Generative AI complements screening by proposing novel chemical matter optimized for multi-objective criteria. Docking-aware generative models or reinforcement-learning-guided molecule generators bias generation toward molecules with favourable docking poses, metal-binding motifs, and desirable ADMET properties. Often, the reward function uses either docking scores or learned affinity predictors, so the generator repetition improves candidate scaffolds rather than simply sampling from the existing library chemotypes. On the whole, the docking-based generative systems help in investigating the chemical space outside conventional databases while preserving structural compatibility with a specified protein pocket.^132^

A representative example is the discovery of adapalene, a third-generation retinoid approved for dermatological use. Adapalene was identified as a potential NDM-1 inhibitor through virtual screening of FDA-approved drugs, molecular docking, and molecular dynamics simulations, using an integrated computational drug repurposing strategy. Docking studies showed that the 40 residues around the binuclear zinc catalytic center, including Asp124, His122, His189, His250, and Cys208, which are catalytically important, provide stable interactions with the ligand, and 100 ns MD simulations confirmed the stability of the protein–ligand complex. Importantly, these computational predictions were subsequently confirmed in experiments using purified NDM-1 enzyme assays and checkerboard antimicrobial susceptibility testing. Adapalene activity strongly inhibited NDM-1 and restored the antibacterial activity of meropenem against clinical NDM-1-producing isolates of *E. coli* and *K. pneumoniae*, demonstrating that computational prioritization can significantly accelerate experimental inhibitor discovery.^72^ AI-driven computational screening workflows have also helped natural-product discovery. Liu *et al.* used virtual screening based on structure, followed by molecular docking and molecular dynamics simulation, to identify magnolol as a selective NDM-1 inhibitor. Site-directed mutagenesis studies and enzyme inhibition were performed to confirm the predictions of this computational analysis, which suggested that Lys211, Gly219, Leu218, His250, and Ser217 are involved in stable hydrophobic interactions. The IC50 of magnolol is 6.47 µg mL^−1^, and it directly binds to the catalytic pocket without displacing the catalytic zinc ions, whereas it does not restore meropenem susceptibility in any of the tested *E. coli* strains. The IC50 value of magnolol is 6.47 µg mL^−1^ and did not restore meropenem susceptibility in any of the tested *E. coli* strains, whereas it directly bound to the catalytic pocket without displacing any catalytic zinc ion. The very good agreement between computational predictions and biochemical validation highlights the utility of natural-product screening based on computation.^93^ Likewise, Yang *et al.* used structure-based virtual screening, molecular docking, molecular dynamics simulations, and experimental testing to identify carnosic acid. Computational modeling showed that carnosic acid binds to an allosteric site rather than directly coordinating the catalytic zinc ions, leading to conformational changes that decrease catalytic activity. Site-directed mutagenesis of Phe46, Leu65, and Thr94 revealed the predicted binding mode, and microbiological assays confirmed the synergistic restoration of meropenem activity against NDM-1-producing bacteria. This is an example of how computational methods can be used to identify active-site and allosteric inhibitors that would otherwise go undetected with traditional screening strategies.^97^ Similar computational–experimental approaches have also identified baicalin, PHT427, and morin as potential inhibitors of NDM-1. Baicalin was identified *via* structure-based virtual screening and subsequently validated for low micromolar activity against the purified enzyme and for restoring carbapenem susceptibility in carbapenem-resistant *E. coli*. PHT427 was discovered through computational high-throughput screening, molecular docking, surface plasmon resonance, fluorescence quenching, zinc supplementation, and mutagenesis experiments, all of which demonstrated its dual mode of action: zinc coordination and interactions with active-site residues. In recent years, virtual screening along with molecular docking and molecular dynamics (MD) simulations led to the identification of morin as an inhibitor candidate whose activity was confirmed by enzyme inhibition assays, antimicrobial synergy studies, and *in vivo* murine infection models, in which the combination therapy with meropenem was found to greatly reduce bacterial burden and inflammatory responses. While these promising breakthroughs are significant, it is crucial to note that the discovery of NDM-1 inhibitors using AI remains a developing process. The majority of published studies do not use full autonomy deep learning or generative AI platforms, but rather computational prioritization, molecular docking, and molecular dynamics simulations. Moreover, the number of compounds predicted computationally that have advanced beyond biochemical characterization and animal infection models is relatively small, and none have been evaluated for clinical use as NDM-1 inhibitors. For now, AI should be considered a hypothesis-generation and lead-prioritization tool alongside traditional drug discovery methods in experimental microbiology, medicinal chemistry, and structural biology. The prospects of integrating large experimental datasets, explainable machine-learning algorithms, generative molecular design, and automated experimental validation are likely to significantly expedite the clinical development of NDM-1 inhibitors.

### 6.4. Pharmacophore modelling, predictive ADMET, and *de novo* molecule design

Pharmacophore modelling remains a strong technique for identifying candidate inhibitors of NDM-1. In one recent approach, a 3D pharmacophore model was generated from the known binding geometry of hydrolysed β-lactam substrates to NDM-1 and was used to screen large chemical libraries. The workflow highlighted candidate molecules such as ZINC29142850, ZINC78607001, and ZINC94303138 that match essential features, including metal-coordinating moieties and hydrogen-bond acceptor/donor placement, and that exhibited favourable docking scores and binding modes in molecular-dynamics simulations.^133^ This type of pharmacophore-based screening helps to enrich for plausible zinc-binding inhibitors and filter out compounds unlikely to coordinate properly with the active-site zinc ions.

Because many putative NDM-1 inhibitors rely on metal chelation, there is a high risk of off-target metal binding or poor drug-like properties, particularly in terms of solubility, permeability, and toxicity. To this end, modern drug-discovery pipelines incorporate early predictive ADMET screening. Recently, graph neural network-based predictors or deep learning-based property estimators have become popular for determining physicochemical and pharmacokinetic properties. These predictive models, in conjunction with pharmacophore and docking filters, enable multi-parameter optimization and increase the likelihood that computational hits are both chemically tractable and biologically tolerable in downstream assays.^134^ Beyond the filtering of existing libraries, *de novo* molecule generation using generative AI has become a powerful method for exploring chemical space far beyond that catalogued in conventional databases. For example, the generative tensorial reinforcement learning model GENTRL demonstrated the practical feasibility of AI-driven *de novo* design, generating novel molecules in ∼21 days, with several showing potent biochemical activity against a kinase target upon experimental validation.^135^ Similarly, recent open-source tools like REINVENT 4 allow for scaffold-hopping, linker optimization, and generation of entirely new chemical cores, guided by reinforcement learning, while enabling constraints on properties such as synthetic feasibility, drug-likeness, and physicochemical parameters.^136^

Such a combination of pharmacophore modelling, ML-based ADMET prediction, and generative design holds considerable promise for NDM-1. The AI-enhanced design strategy can expand the candidate inhibitor chemical space beyond existing databases while increasing the likelihood that hit compounds are viable as real drug leads.

### 6.5. Challenges in clinical translation and the potential emergence of resistance to NDM-1 inhibitors

In spite of numerous NDM-1 inhibitors being discovered in the literature, there is still no approved drug that targets NDM enzymes in particular. These drawbacks are explained by several factors that are related to the basic science and translational aspects of the problem. In contrast to serine β-lactamases, which have a conserved catalytic serine residue that is an easy target for irreversible inhibition through covalent reaction, NDM-1 uses a dinuclear Zn(ii)-mediated catalysis inside an extremely solvent-exposed and flexible active site. Consequently, many inhibitors employ metal chelation that is non-selective and can bind to host metalloenzymes.

The different types of inhibitors that have been studied to date possess varying levels of strengths and weaknesses. Thio-based inhibitors have shown strong enzymatic inhibition properties because of the ability to coordinate with zinc, but are often unstable metabolically, oxidized physiologically, and indiscriminate in interacting with other cellular thiols. On the other hand, hydroxamates and similar chelators have good metal-binding properties but can potentially inhibit metalloenzymes in the host. Repurposed drugs like ebselen, disulfiram, and auranofin have known safety records, but their reactivity and mechanism of action can limit their clinical use.

The natural compounds usually show good selectivity and greater diversity in terms of their structure, but they have some disadvantages including low solubility, poor bioavailability, high rate of metabolism, and lack of *in vivo* validation. From all the existing methods, bicyclic boronates like taniborbactam and xeruborbactam can be considered the most advanced since they are characterized by effective enzyme inhibition and better pharmacokinetics properties.

Resistance against inhibitors targeting NDM enzymes can also be an issue in the future. It should be noted that the great diversity of NDM mutants proves that this enzyme has high evolutionary potential. Changes in enzyme structure, which include single mutations of amino acids outside the active zinc sites, were demonstrated to affect enzyme stability, binding of zinc ions, and substrates. Specifically, the NDM-9 mutant was found to have lower susceptibility to taniborbactam compared to NDM-1, having one different amino acid in its structure. These facts indicate that new resistant mutants can arise due to the selective pressure of future inhibitors.

Moreover, NDM-based resistance does not usually occur in a single fashion; often, its presence is accompanied by that of reduced porins, efflux pump overexpression, biofilm production, and the presence of other β-lactamases, limiting the efficacy of highly active NDM inhibitors. Hence, the future success of such drugs would need to be demonstrated by inhibitors active against multiple variants of NDM, good pharmacokinetics and safety characteristics, and their administration as part of combination therapies that counteract alternative resistance methods. Such factors explain why, despite considerable scientific efforts and several promising discoveries, a specific NDM inhibitor still remains an elusive goal in antimicrobial drug discovery.

## 7. Conclusion

The rise of the NDM-1 has become synonymous with the antibiotic resistance crisis. NDM-1 has spread across the globe since its discovery and has rendered almost all β-lactam antibiotics, including carbapenems, ineffective, presenting a serious health risk. Its 3D structure, which comprises a conserved αβ/βα fold and highly dynamic loop regions, allows a broad substrate specificity and efficient catalytic activity. The bifunctional zinc hydrolytic mechanism along with loop flexibility allows for effective hydrolysis of various β-lactams, and it also presents a challenge to the development of NDM-1 inhibitors.

NDM-expressing bacteria are resistant due to β-lactam hydrolysis, porin loss, extrusion through efflux pumps, and biofilm formation, all of which contribute to conferring high-level resistance, reducing antibiotic penetration and the formation of environments conductive to genetic transfer. Specifically, biofilms act as reservoirs for the horizontal dissemination of NDM and other resistance determinants, in both clinical and environmental settings. However, despite the difficulties, promising leads for inhibitors have emerged. New technologies signify or promise new hope. The CRISPR-Cas systems are being explored as highly specific tools for removing resistance genes, such as NDM, from bacterial genomes, which could reduce the population of resistant bacteria. Nanotechnology approaches such as silver and zinc oxide nanoparticles or polymeric nanoparticles offer new avenues for the delivery of antibiotics or inhibitors directly to bacteria, increasing their penetration into biofilms and facilitating controlled release at infection sites. Machine learning and artificial intelligence are transforming the drug discovery process by facilitating high-velocity virtual screening, docking, and synthesis of new scaffolds supported by Zn coordination and loop dynamics. Such interdisciplinary solutions, from molecular biology to nanotechnology and artificial intelligence, are the future of NDM-1 resistance. Finally, NDM-1 illustrates the adaptive resistance, combining with structural flexibility, mutational evolution, and environmental adaptability in a single protein to render β-lactams ineffective. Only with concerted global efforts for research, innovation, and public health approaches can we hope for a return to efficacy of β-lactams and prevent the spectre of multidrug-resistant superbugs.

## Author contributions

Akankhya Mohanty: conceptualisation and design of the article, collection and compilation of relevant literature, figure and table design, drafting, review and editing. Rajesh Kumar Sahoo: conceptualisation and design of the article, review and editing. A. Swaroop Sanket: review and editing. Ellojita Rout: review and editing.

## Conflicts of interest

The authors declare that there are no conflicts of interest.

## Supplementary Material

RA-016-D6RA04475A-s001

## Data Availability

No primary research results, software or code have been included, and no new data were generated or analysed as part of this review.
