# Supplementary material for: Combating New Delhi metallo-beta-lactamase-1 (NDM-1): an integrated review of inhibitor development and next-generation therapeutic platforms
Source: RSC Adv. 2026 Sep 4;16(42):49922–53. doi: 10.1039/d6ra04475a (PMC13544147; doi:10.1039/d6ra04475a)
Supplement: Supplementary file 1 [file RA-016-D6RA04475A-s001.pdf]

This journal is © The Royal Society of Chemistry 2026

Combating New Delhi Metallo-beta-lactamase (NDM-1): An Integrated Review of Inhibitors Development and Next Generation Therapeutic Platform

Supplementary Tables:

Table S1: List of NDM variants reported in the literature

Variant	Amino acid Substitution	Location	Source Organisms	References
NDM-1	None	Features a standard αβ/βα sandwich fold with an active site containing two Zn(II) ions. It is a lipoprotein anchored to the bacterial outer membrane.	*Klebsiella pneumoniae and Escherichia coli*	1
NDM-2	Pro28Ala	Between the lipidation box and β1 strand	*Acinetobacter baumannii*	2
NDM-3	Asp95Asn	α1 helix	*Escherichia coli*	3
NDM-4	Met154Leu	Between turn 2 and β10	*Escherichia coli*	4
NDM-5	Val88Leu, Met154Leu	β6 strand; between turn 2 and β10	*Escherichia coli*	4, 5
NDM-6	Ala233Val	α8 helix	*Escherichia coli, Acinetobacter baumannii*	6, 7
NDM-7	Asp130Asn, Met154Leu	α3 helix; region between turn 2 and β10	*Escherichia coli*	8, 9
NDM-8	Asp130Gly, Met154Leu	α3 helix; region between turn 2 and β10	*Escherichia coli*	10
NDM-9	Glu152Lys	Turn 2	*Klebsiella pneumoniae*	11
NDM-10	Arg32Ser, Gly36Asp, Gly69Ser, Ala74Thr, Gly200Arg	Between the lipidation box and β1; β1; turn 1; β5; between β14 and β15	*Klebsiella pneumoniae*	12
NDM-11	Met154Val	Region between turn 2 and β10	*Escherichia coli*	13

NDM-12	Met154Leu, Gly222Asp	Region between turn 2 and β10; α7 helix	*Escherichia coli*	14
NDM-13	Asp95Asn, Met154Leu	α1 helix; region between turn 2 and β10	*Escherichia coli*	14
NDM-14	Asp130Gly	α3 helix	*Acinetobacter lwoffii*	15
NDM-15	Met154Leu, Ala233Val	Region between turn 2 and β10; α8 helix	*Escherichia coli*	16
NDM-16	Arg264His	α9 helix	*Klebsiella pneumoniae*	17
NDM-17	Val88Leu, Met154Leu, Glu170Lys	β6 strand; region between turn 2 and β10; between β11 and α5 helix	*Escherichia coli*	18
NDM-18	The tandem repeat of QRFGD at positions 44 to 48	β2; between β2 and β3	*Escherichia coli*	19
NDM-19	Asp130Asn, Met154Leu, Ala233Val	α3 helix; region between turn 2 and β10; α8 helix	*Escherichia coli*	20
NDM-20	Val88Leu, Met154Leu, Arg270His	β6 strand; region between turn 2 and β10	*Escherichia coli*	21
NDM-21	Gly69Ser, Val88Leu, Met154Leu	Turn 1; β6 strand; between turn 2 and β10	*Escherichia coli*	22
NDM-22	Met248Leu	Between β16 and β17	*Enterobacter cloacae*	23
NDM-23	Ile101Leu	α1 helix	*Klebsiella pneumoniae*	24
NDM-24	Val88Leu	β6 strand	*Providencia stuartii*	25

NDM-25	Ala55Ser	β3 strand	*Klebsiella pneumoniae*	26
NDM-26	Val88Leu, Met154Leu, Gly222Ser	β6 strand; region between turn 2 and β10; α7 helix	*Escherichia coli*	27
NDM-27	Asp95Asn, Ala233Val	α1 helix; α8 helix	*Escherichia coli and Klebsiella pneumoniae*	28
NDM-28	Ala266Val	α9 helix	*Klebsiella pneumoniae*	29
NDM-29	Asp130Asn	α3 helix	*Escherichia coli*	30
NDM-30	Asp223Tyr	α7 helix	*Klebsiella oxytoca*	31
NDM-31	Pro171Thr	α5 helix	*Citrobacter werkmanii*	31
NDM-33	Ala72Thr, Val88Leu, Met154Leu	β6 strand; between turn 2 and β10	*Escherichia coli*	32
NDM-34	Leu49Met, Leu54Ile, Val88Leu	Region between β2 and β3; β3; β6	*Vibrio parahaemolyticus*	33
NDM-35	Gly84Asp, Val88Leu, Met154Leu	Region between β5 and β6; β6; between turn 2 and β10	*Escherichia coli*	34
NDM-36	Val88Leu, Met154Leu, Ala233Val, His261Tyr	β6 strand; region between turn 2 and β10; α8 helix; α9 helix	*Escherichia coli*	35
NDM-37	Val88Leu, Met154Leu, His261Tyr	β6 strand; region between turn 2 and β10; α9 helix	*Escherichia coli*	35
NDM-38	Met27Val	Between the lipidation box and β1	*Providencia rettgeri*	36

NDM-39	Pro4Leu, Val88Leu, Met154Leu	Signal peptide; β6; between turn 2 and β10	*Klebsiella pneumoniae*	37
NDM-40	Pro4Arg	Signal peptide	*Acinetobacter baumannii*	38
NDM-41	Leu13Tyr, Val88Leu, Met154Leu	Signal peptide; β6; between turn 2 and β10	*Klebsiella pneumoniae*	39
NDM-58	Pro185Ser	located close to the zinc-binding residue at position H190	*Pseudomonas aeruginosa*	40
NDM-63	Gly69Ser, Phe70 deletion, Ala72His	L3 loop	*Klebsiella pneumoniae*	41

Note:

Table S1 provides a non-exhaustive list of officially designated NDM variants identified through our predefined literature and database search. Variants were included when an official NDM designation and information on amino-acid substitutions and source organism were available in peer-reviewed publications or curated NCBI/GenBank records. The search covered information available up to 15th May 2026; therefore, variants reported or deposited after this date are not included.

Table S2: Descriptive list of the reported inhibitors of NDM-1

**Type of Inhibitors**	**Strategy**	**Mechanism of Inhibition**	**Advantages**	**Limitations**	**Key References**
Thiol-based inhibitors (captopril, thiorphan, dimercaprol)	Zn^{2+} chelation	Thiol coordinates Zn1/Zn2, disrupts catalytic hydroxide	Strong inhibition (low µM IC_{50}), established scaffold	Oxidation, off-target thiol binding, low selectivity	[42]
Hydroxamates	Bidentate Zn chelation	Coordinate both Zn ions, inhibit catalysis	Effective MIC reduction, strong binding	Toxicity, off-target effects	[43]
Spiro-indoline-thiadiazoles (SITs)	Metal chelation + scaffold rearrangement	Ring opening exposes S/N → Zn binding	Synergy with carbapenems	Poor solubility, stability issues	[44]
Boronic acids / Bicyclic boronates (taniborbactam, xeruborbactam)	Transition-state mimicry	Mimic tetrahedral intermediate, coordinate Zn ions	Dual SBL/MBL inhibition, clinical potential	Resistance mutations (e.g., NDM-9)	[45], [46]
Adapalene (Repurposed-drug)	Active-site binding	Interacts with His/Asp residues, stabilizes inactive complex	FDA-approved, synergistic	Poor PK, limited in vivo data	[47]

Ebselen (Repurposed-drug)	Covalent + Zn displacement	Binds Zn and Cys residues	Strong MIC reduction	Non-specific reactivity, toxicity	48,49
Disulfiram (Repurposed-drug)	Covalent modification	Forms S–S bond with Cys208, releases Zn^{2+}	Strong synergy, known drug	Severe side effects	50
Auranofin (Repurposed-drug)	Metal exchange (Zn→Au)	Replaces Zn with Au, disrupts catalytic center	Dual inhibition, irreversible action	Toxicity, unclear mechanism	51
Magnolol (Natural products)	Active-site blockade	Hydrophobic + H-bond interactions block substrate access	High specificity, non-chelation	Poor bioavailability	52
Carnosic acid (Natural products)	Allosteric inhibition	Binds non-active site → conformational change	Novel mechanism	Moderate potency	53
Morin (Natural products)	Zn interaction + residue binding	Interacts with catalytic residues and Zn^{2+}	Strong synergy, in vivo efficacy	Lack of IC_{50} data	54
Baicalin (Natural products)	Active-site interaction	Disrupts Zn coordination and catalytic geometry	Low µM IC_{50}, synergistic	Poor PK, limited clinical data	55

PHT427 (Synthetic small molecule)	Zn^{2+} interaction + active-site residue binding	Interacts with Zn^{2+} and active-site residues; Asn220 and Gln123 contribute to binding; zinc-supplementation supports Zn-dependent inhibition	IC_{50} 1.42 µM; restored meropenem susceptibility in vitro	Limited in vivo and clinical validation; lead requires further optimization	56
Aspergillomarasmine A (AMA) (Natural products)	Zn stripping	Removes Zn^{2+} (especially Zn2 site)	Very potent, in vivo validated	Resistance via Zn affinity mutations	57
EDTA (Metal Chelators)	Broad Zn chelation	Strips Zn ions from the active site	Strong in vitro synergy	Non-specific, toxic	58
Dipicolinic acid (DPA) (Metal Chelators)	Controlled Zn coordination	Forms enzyme–Zn–inhibitor complex	More selective than EDTA	Off-target chelation	59
Silver nanoparticles (AgNPs) (Nanomaterials)	Multi-target antibacterial	Multi-target antibacterial activity; not proven to be direct purified NDM-1 inhibition	Broad-spectrum, strong synergy	Activity against resistant/MBL-producing bacteria	60
Graphene oxide (GO) (Nanomaterials)	Structural disruption	β-lactamase interaction/structural disruption; direct NDM-1 specificity not established	Broad β-lactamase inhibition	Broad β-lactamase interaction reported	61

References:

1 D. Yong, M. A. Toleman, C. G. Giske, H. S. Cho, K. Sundman, K. Lee and T. R. Walsh, Characterization of a New Metallo-β-Lactamase Gene, $bla_{NDM\text{-}1}$, and a Novel Erythromycin Esterase Gene Carried on a Unique Genetic Structure in *Klebsiella pneumoniae* Sequence Type 14 from India, *Antimicrob Agents Chemother*, 2009, **53**, 5046–5054.

2 M. Kaase, P. Nordmann, T. A. Wichelhaus, S. G. Gatermann, R. A. Bonnin and L. Poirel, NDM-2 carbapenemase in *Acinetobacter baumannii* from Egypt, *Journal of Antimicrobial Chemotherapy*, 2011, **66**, 1260–1262.

3 T. Tada, T. Miyoshi-Akiyama, K. Shimada and T. Kirikae, Biochemical Analysis of Metallo-β-Lactamase NDM-3 from a Multidrug-Resistant *Escherichia coli* Strain Isolated in Japan, *Antimicrob Agents Chemother*, 2014, **58**, 3538–3540.

4 L. Dortet, L. Poirel and P. Nordmann, Worldwide Dissemination of the NDM-Type Carbapenemases in Gram-Negative Bacteria, *BioMed Research International*, **2014**, 1–12.

5 A. Sassi, L. Loucif, S. K. Gupta, M. Dekhil, H. Chettibi and J.-M. Rolain, NDM-5 Carbapenemase-Encoding Gene in Multidrug-Resistant Clinical Isolates of *Escherichia coli* from Algeria, *Antimicrob Agents Chemother*, 2014, **58**, 5606–5608.

6 D. A. Williamson, H. E. Sidjabat, J. T. Freeman, S. A. Roberts, A. Silvey, R. Woodhouse, E. Mowat, K. Dyet, D. L. Paterson, T. Blackmore, A. Burns and H. Heffernan, Identification and molecular characterisation of New Delhi metallo-β-lactamase-1 (NDM-1)- and NDM-6-producing *Enterobacteriaceae* from New Zealand hospitals, *International Journal of Antimicrobial Agents*, 2012, **39**, 529–533.

7 C. Kigen, V. Natasha, J. Wachira, E. Odoyo, A. Wataka, S. Ondolo, L. Preston, T. Luo, M. J. Martin, L. P. Sang and L. Musila, First report of New Delhi metallo-β-lactamase-6

(blaNDM-6) gene in Africa identified in a carbapenem-resistant Acinetobacter baumannii clinical isolate, *International Journal of Infectious Diseases*, 2026, **162**, 108176.

8 J. Xu and F. He, Characterization of a NDM-7 carbapenemase-producing *Escherichia coli* ST410 clinical strain isolated from a urinary tract infection in China, *IDR*, 2019, **12**, 1555–1564.

9 S. Gottig, A. G. Hamprecht, S. Christ, V. A. J. Kempf and T. A. Wichelhaus, Detection of NDM-7 in Germany, a new variant of the New Delhi metallo-β-lactamase with increased carbapenemase activity, *Journal of Antimicrobial Chemotherapy*, 2013, **68**, 1737–1740.

10 A. Makena, J. Brem, I. Pfeffer, R. E. J. Geffen, S. E. Wilkins, H. Tarhonskaya, E. Flashman, L. M. Phee, D. W. Wareham and C. J. Schofield, Biochemical characterization of New Delhi metallo-β-lactamase variants reveals differences in protein stability, *Journal of Antimicrobial Chemotherapy*, 2015, **70**, 463–469.

11 M. Falcone, C. Giordano, S. Barnini, G. Tiseo, A. Leonildi, P. Malacarne, F. Menichetti and A. Carattoli, Extremely drug-resistant NDM-9-producing ST147 *Klebsiella pneumoniae* causing infections in Italy, May 2020, *Eurosurveillance*, DOI:10.2807/1560-7917.ES.2020.25.48.2001779.

12 A. Khajuria, A. K. Praharaj, M. Kumar and N. Grover, Presence of a novel variant NDM-10, of the New Delhi metallo-beta-lactamase in a *Klebsiella pneumoniae* isolate, *Indian Journal of Medical Microbiology*, 2016, **34**, 121–123.

13 M. Rahman, C. Mukhopadhyay, R. P. Rai, S. Singh, S. Gupta, A. Singh, A. Pathak and K. N. Prasad, Novel variant NDM-11 and other NDM-1 variants in multidrug-resistant *Escherichia coli* from South India, *Journal of Global Antimicrobial Resistance*, 2018, **14**, 154–157.

14 T. Tada, B. Shrestha, T. Miyoshi-Akiyama, K. Shimada, H. Ohara, T. Kirikae and B. M. Pokhrel, NDM-12, a Novel New Delhi Metallo-β-Lactamase Variant from a Carbapenem-Resistant Escherichia coli Clinical Isolate in Nepal, *Antimicrob Agents Chemother*, 2014, **58**, 6302–6305.

15 D. Zou, Y. Huang, X. Zhao, W. Liu, D. Dong, H. Li, X. Wang, S. Huang, X. Wei, X. Yan, Z. Yang, Y. Tong, L. Huang and J. Yuan, A Novel New Delhi Metallo-β-Lactamase Variant, NDM-14, Isolated in a Chinese Hospital Possesses Increased Enzymatic Activity against Carbapenems, *Antimicrob Agents Chemother*, 2015, **59**, 2450–2453.

16 G. Bahr, L. Vitor-Horen, C. R. Bethel, R. A. Bonomo, L. J. González and A. J. Vila, Clinical Evolution of New Delhi Metallo-β-Lactamase (NDM) Optimizes Resistance under Zn(II) Deprivation, *Antimicrobial Agents and Chemotherapy*, DOI:10.1128/AAC.01849-17.

17 K. M. Kazmierczak, S. Rabine, M. Hackel, R. E. McLaughlin, D. J. Biedenbach, S. K. Bouchillon, D. F. Sahm and P. A. Bradford, Multiyear, Multinational Survey of the Incidence and Global Distribution of Metallo-β-Lactamase-Producing *Enterobacteriaceae* and *Pseudomonas aeruginosa*, *Antimicrob Agents Chemother*, 2016, **60**, 1067–1078.

18 Z. Liu, Y. Wang, T. R. Walsh, D. Liu, Z. Shen, R. Zhang, W. Yin, H. Yao, J. Li and J. Shen, Plasmid-Mediated Novel bla_{NDM-17} Gene Encoding a Carbapenemase with Enhanced Activity in a Sequence Type 48 *Escherichia coli* Strain, *Antimicrob Agents Chemother*, 2017, **61**, e02233-16.

19 W. Wu, Y. Feng, G. Tang, F. Qiao, A. McNally and Z. Zong, NDM Metallo-β-Lactamases and Their Bacterial Producers in Health Care Settings, *Clin Microbiol Rev*, 2019, **32**, e00115-18.

20 S. Mancini, P. M. Keller, M. Greiner, V. Bruderer and F. Imkamp, Detection of NDM-19, a novel variant of the New Delhi metallo-β-lactamase with increased carbapenemase activity under zinc-limited conditions, in Switzerland, *Diagnostic Microbiology and Infectious Disease*, 2019, **95**, 114851.

21 Z. Liu, J. Li, X. Wang, D. Liu, Y. Ke, Y. Wang and J. Shen, Novel Variant of New Delhi Metallo-β-lactamase, NDM-20, in *Escherichia coli*, *Front. Microbiol.*, 2018, **9**, 248.

22 L. Liu, Y. Feng, A. McNally and Z. Zong, bla NDM-21, a new variant of blaNDM in an Escherichia coli clinical isolate carrying blaCTX-M-55 and rmtB, *Journal of Antimicrobial Chemotherapy*, 2018, **73**, 2336–2339.

23 https://www.ncbi.nlm.nih.gov/protein/AWI33311

24 N. García-González, B. Beamud, B. Fuster, S. Giner, M. V. Domínguez, A. Sánchez, J. Sevilla, T. M. Coque, C. Gimeno, F. González-Candelas, N. García-González, F. González-Candelas, B. Fuster, N. Tormo, C. Salvador, C. Gimeno, V. Domínguez, S. Giner, J. Colomina, D. Navarro, L. Martínez, A. Burgos, O. Martínez, B. Gomila-Sard, R. Moreno, I. Vidal, A. Sánchez, J. C. Rodriguez and V. Sánchez-Hellín, Tracking the Emergence and Dissemination of a $bla_{\text{NDM-23}}$ Gene in a Multidrug Resistance Plasmid of *Klebsiella pneumoniae*, *Microbiol Spectr*, 2023, **11**, e02585-22.

25 Z. Liu, A. Piccirilli, D. Liu, W. Li, Y. Wang and J. Shen, Deciphering the Role of V88L Substitution in NDM-24 Metallo-β-Lactamase, *Catalysts*, 2019, **9**, 744.

26 M. F. El-Badawy, S. W. El-Far, S. S. Althobaiti, F. I. Abou- Elazm and M. M. Shohayeb, The First Egyptian Report Showing the Co-Existence of $\text{bla}_{\text{NDM-25}}$, $\text{bla}_{\text{OXA-23}}$, $\text{bla}_{\text{OXA-181}}$, and $\text{bla}_{\text{GES-1}}$ Among Carbapenem-Resistant *K. pneumoniae* Clinical Isolates Genotyped by BOX-PCR, *IDR*, 2020, **13**, 1237–1250.

27 https://www.ncbi.nlm.nih.gov/protein/AYN72942

28 https://www.ncbi.nlm.nih.gov/protein/AYP70146

29 https://www.ncbi.nlm.nih.gov/protein/QAT97614

30 Y. Zhu, X. Jia, P. Jia, X. Li and Q. Yang, Genetic and Phenotypic Characterization of the Novel Metallo-β-Lactamase NDM-29 From *Escherichia coli*, *Front. Microbiol.*, 2021, **12**, 743981.

31 J. Park, S.-J. Yun, E. Shin, J. S. Kim, S.-H. Park, H.-S. Pyeon, M.-K. Kim, S. Joo, H. J. Jeong, J. Chun, K. Hwang and J. Kim, First identification of novel variants of New Delhi metallo-β-lactamase, NDM-30 and NDM-31, in the Republic of Korea, *Journal of Global Antimicrobial Resistance*, 2022, **29**, 20–22.

32 T. Wang, Y. Zhou, C. Zou, Z. Zhu, J. Zhu, J. Lv, X. Xie, L. Chen, S. Niu and H. Du, Identification of a Novel bla_{NDM} Variant, $bla_{\text{NDM-33,}}$ in an *Escherichia coli* Isolate from Hospital Wastewater in China, *mSphere*, 2021, **6**, e00776-21.

33 H. Wang, J. R. Strich, S. K. Drake, Y. Chen, J.-H. Youn, A. Z. Rosenberg, M. Gucek, J. P. Dekker and A. F. Suffredini, Rapid Identification of New Delhi Metallo-β-Lactamase (NDM) Using Tryptic Peptides and LC-MS/MS, *Antimicrob Agents Chemother*, 2019, **63**, e00461-19.

34 L. Poirel, J. M. Ortiz De La Rosa, Z. Sakaoglu, A. Kusaksizoglu, M. Sadek and P. Nordmann, NDM-35-Producing ST167 *Escherichia coli* Highly Resistant to β-Lactams Including Cefiderocol, *Antimicrob Agents Chemother*, 2022, **66**, e00311-22.

35 W. Ma, B. Zhu, W. Wang, Q. Wang, X. Cui, Y. Wang, X. Dong, X. Li, J. Ma, F. Cheng, X. Shi, L. Chen, S. Niu and M. Hao, Genetic and enzymatic characterization of two novel blaNDM-36, -37 variants in *Escherichia coli* strains, *Eur J Clin Microbiol Infect Dis*, 2023, **42**, 471–480.

36 https://www.ncbi.nlm.nih.gov/protein/QWO25674

37 https://www.ncbi.nlm.nih.gov/protein/QYZ89892

38 https://www.ncbi.nlm.nih.gov/protein/QYZ89893

39 https://www.ncbi.nlm.nih.gov/protein/UZC76859

40 M. Karthik, N. K. Panchal, M. T, Y. D. Bakthavatchalam, A. Neeravi, B. Abirami, K. Walia and B. Veeraraghavan, Evolutionary insights into NDM variants: Identification and functional analysis of novel NDM-58 in *Pseudomonas aeruginosa*, *Microbial Pathogenesis*, 2025, **204**, 107574.

41 S. R. Boncompagni, A. Antonelli, B. Casciato, F. Pieralli, A. J. Vila, D. M. Moreno, T. Giani and G. M. Rossolini, NDM-63: a novel NDM metallo-β-lactamase variant in the L3 loop, from a *Klebsiella pneumoniae* clinical isolate, *Antimicrob Agents Chemother*, 2026, **70**, e01286-25.

42 D. King and N. Strynadka, Crystal structure of New Delhi metallo-β-lactamase reveals molecular basis for antibiotic resistance, *Protein Science*, 2011, **20**, 1484–1491.

43 X.-R. Wu, W.-Y. Chen, L. Liu and K.-W. Yang, Discovery of hydroxamate as a promising scaffold dually inhibiting metallo- and serine-β-lactamases, *European Journal of Medicinal Chemistry*, 2024, **265**, 116055.

44 T. Wang, K. Xu, L. Zhao, R. Tong, L. Xiong and J. Shi, Recent research and development of NDM-1 inhibitors, *European Journal of Medicinal Chemistry*, 2021, **223**, 113667.

45 X. Li, D. Zhao, W. Li, J. Sun and X. Zhang, *International Journal of Molecular Sciences*, 2022, 23, 197.

46 L. Sánchez-Peña, S. Rodríguez-Pallares, P. Aja-Macaya, T. Blanco-Martín, L. González-Pinto, G. Pérez-Rodríguez, C. Le Terrier, I. Portillo-Calderón, E. Recacha, C. Riazzo, J. C. Vázquez-Ucha, A. Beceiro, B. Aracil, J. Oteo-Iglesias, L. Martínez-Martínez, L. Poirel, G. Bou and J. Arca-Suárez, Broad spectrum of β-lactamase coverage and potent antimicrobial activity of xeruborbactam in combination with meropenem

against carbapenemase-producing Enterobacterales, including strains resistant to new β-lactam/β-lactamase inhibitor combinations, *Antimicrob Agents Chemother*, 2025, **69**, e00533-25.

47 S. S, H. N, A. Fasim, S. S. More and S. Das Mitra, Identification of a potential inhibitor for New Delhi metallo-β-lactamase 1 (NDM-1) from FDA approved chemical library- a drug repurposing approach to combat carbapenem resistance, *Journal of Biomolecular Structure and Dynamics*, 2023, **41**, 7700–7711.

48 M. Parnham and H. Sies, Ebselen: prospective therapy for cerebral ischaemia, *Expert Opinion on Investigational Drugs*, 2000, **9**, 607–619.

49 J. Chiou, S. Wan, K.-F. Chan, P.-K. So, D. He, E. W. Chan, T. Chan, K. Wong, J. Tao and S. Chen, Ebselen as a potent covalent inhibitor of New Delhi metallo-β-lactamase (NDM-1), *Chemical Communications*, 2015, **51**, 9543–9546.

50 C. Chen, K.-W. Yang, L.-Y. Wu, J.-Q. Li and L.-Y. Sun, Disulfiram as a potent metallo-β-lactamase inhibitor with dual functional mechanisms, *Chem. Commun.*, 2020, **56**, 2755–2758.

51 I. Tolbatov and A. Marrone, Auranofin Targeting the NDM-1 Beta-Lactamase: Computational Insights into the Electronic Configuration and Quasi-Tetrahedral Coordination of Gold Ions, *Pharmaceutics*, 2023, **15**, 985.

52 S. Liu, Y. Zhou, X. Niu, T. Wang, J. Li, Z. Liu, J. Wang, S. Tang, Y. Wang and X. Deng, Magnolol restores the activity of meropenem against NDM-1-producing Escherichia coli by inhibiting the activity of metallo-beta-lactamase, *Cell Death Discovery*, 2018, **4**, 28.

53 Y. Yang, Y. Guo, Y. Zhou, Y. Gao, X. Wang, J. Wang and X. Niu, Discovery of a Novel Natural Allosteric Inhibitor That Targets NDM-1 Against *Escherichia coli*, *Front. Pharmacol.*, 2020, **11**, 581001.

54 Q. Ren, B. Zhang, M. Wang, T. Wang, W. Zhang, L. Du, M. Yu, Y. Li and X. Li, Morin combined with meropenem is a potent inhibitor of NDM-1 against NDM-1-producing *E. coli*, *Sci Rep*, 2025, **15**, 24169.

55 C. Shi, J. Bao, Y. Sun, X. Kang, X. Lao and H. Zheng, Discovery of Baicalin as NDM-1 inhibitor: Virtual screening, biological evaluation and molecular simulation, *Bioorganic Chemistry*, 2019, **88**, 102953.

56 X. Li, Q. Wang, J. Zheng, Y. Guan, C. Liu, J. Han, S. Liu, T. Liu, C. Xiao, X. Wang and Y. Liu, PHT427 as an effective New Delhi metallo-β-lactamase-1 (NDM-1) inhibitor restored the susceptibility of meropenem against Enterobacteriaceae producing NDM-1, *Frontiers in Microbiology*, DOI:10.3389/fmicb.2023.1168052.

57 A. M. King, S. A. Reid-Yu, W. Wang, D. T. King, G. De Pascale, N. C. Strynadka, T. R. Walsh, B. K. Coombes and G. D. Wright, Aspergillomarasmine A overcomes metallo-β-lactamase antibiotic resistance, *Nature*, 2014, **510**, 503–506.

58 S. M. Rudresh, G. S. Ravi and Y. Raksha, In Vitro Efficacy of Biocompatible Zinc Ion Chelating Molecules as Metallo-β-Lactamase Inhibitor among NDM Producing Escherichia coli, *J Lab Physicians*, 2023, **15**, 062–068.

59 T. S. Shkuratova, V. G. Grigorenko, I. P. Andreeva, V. A. Litvinova, N. E. Grammatikova, A. S. Tikhomirov, A. M. Egorov and A. E. Shchekotikhin, New derivatives of dipicolinic acid as metallo-β-lactamase NDM-1 inhibitors, *Med Chem Res*, 2025, **34**, 219–227.

60 S. H. Haji, F. A. Ali and S. T. H. Aka, Synergistic antibacterial activity of silver nanoparticles biosynthesized by carbapenem-resistant Gram-negative bacilli, *Sci Rep*, 2022, **12**, 15254.

61 A. Piccirilli, M. Perilli, F. Brisdelli, D. Mastrippolito, G. D'Olimpio, S. Ershadrad, B. Sanyal and L. Ottaviano, Enzyme graphene oxide interaction: the case system of β-lactamases, *RSC Advances*, 2025, **15**, 21199–21211.
